# Genomic Snapshot of SARS-CoV-2 in Migrants Entering Through Mediterranean Sea Routes

**DOI:** 10.3389/fpubh.2022.846115

**Published:** 2022-03-03

**Authors:** Nicole Grandi, Bianca Paglietti, Roberto Cusano, Gabriele Ibba, Vincenzo Lai, Claudia Piu, Flavia Angioj, Caterina Serra, David J. Kelvin, Enzo Tramontano, Salvatore Rubino

**Affiliations:** ^1^Laboratory of Molecular Virology, Department of Life and Environmental Sciences, University of Cagliari, Cagliari, Italy; ^2^Department of Biomedical Sciences, University of Sassari, Sassari, Italy; ^3^SC Microbiologia e Virologia, Azienda Ospedaliero Universitaria Sassari, Sassari, Italy; ^4^Center for Advanced Studies, Research and Development in Sardinia (CRS4), Science and Technology Park Polaris, Cagliari, Italy; ^5^Department of Microbiology and Immunology, Canadian Center for Vaccinology, Faculty of Medicine, Dalhousie University, Halifax, NS, Canada; ^6^Division of Immunology, International Institute of Infection and Immunity, Shantou University Medical College, Shantou, China; ^7^Istituto di Ricerca Genetica e Biomedica, Consiglio Nazionale Delle Ricerche, Cagliari, Italy

**Keywords:** SARS-CoV-2, COVID-19, coronavirus pandemic, migrants, Libyan route

## Abstract

In December 2019, a novel coronavirus emerged in Wuhan, China, rapidly spreading into a global pandemic. Italy was the first European country to experience SARS-CoV-2 epidemic, and one of the most severely affected during the first wave of diffusion. In contrast to the general restriction of people movements in Europe, the number of migrants arriving at Italian borders via the Mediterranean Sea route in the summer of 2020 had increased dramatically, representing a possible, uncontrolled source for the introduction of novel SARS-CoV-2 variants. Importantly, most of the migrants came from African countries showing limited SARS-CoV-2 epidemiological surveillance. In this study, we characterized the SARS-CoV-2 genome isolated from an asymptomatic migrant arrived in Sardinia *via* the Mediterranean route in September 2020, in comparison with SARS-CoV-2 isolates arrived in Sicily through the Libyan migration route; with SARS-CoV-2 isolates circulating in Sardinia during 2020; and with viral genomes reported in African countries during the same summer. Results showed that our sequence is not phylogenetically related to isolates from migrants arriving in Sicily, nor to isolates circulating in Sardinia territory, having greater similarity to SARS-CoV-2 genomes reported in countries known for being sites of migrant embarkation to Italy. This is in line with the hypothesis that most SARS-CoV-2 infections among migrants have been acquired prior to embarking to Italy, possibly during the travel to or the stay in crowded Libyan immigrant camps. Overall, these observations underline the importance of dedicated SARS-CoV-2 surveillance of migrants arriving in Italy and in Europe through the Mediterranean routes.

## Introduction

At the end of December 2019, an outbreak of a novel coronavirus, SARS-CoV-2 originated in Wuhan, China, and rapidly expanded into a global pandemic causing COVID-19 disease (1). Since then, the pandemic has caused ~250 million confirmed cases including more than 5 million deaths worldwide according to WHO official data (2).

Italy was the first European country to experience the dramatic consequences of COVID-19 spread, and it was one of the most severely affected countries during the first wave of the pandemic (3). The rapidly evolving situation has prompted the Italian authorities to impose tight restrictions on border movements and clamp unprecedented migration measures to limit the spread of the virus, including a statement that Italian ports could not be regarded “safe places” for the disembarkation of people rescued by foreign-flagged vessels (April 2020) (4). The number of migrants/refugees landing on Italian borders via the Mediterranean route in September and October increased by 345% in 2020 compared to the same period in 2019 (4) underscoring the need for rapid surveillance of migrant infections.

This surge, together with COVID-19 risks, poses a significant impact on refugees, asylum-seekers and migrants, as they typically remain in closed or crowded settings (refugee camps and especially boats or rescue ships) where basic public health measures—such as social distancing, appropriate sanitation facilities and self-isolation - are extremely difficult or not impossible adhere to (5, 6). Few studies have genetically characterized SARS-CoV-2 among migrants and refugees, as they are considered a vulnerable population, which underscores the need for genomic surveillance that could lead to the introduction of novel SARS-CoV-2 variants (7). In this pilot study, phylogenetic and virological analyses were performed to investigate the genomic origin of the SARS-CoV-2 isolate obtained from an asymptomatic migrant who tested positive by RT-PCR at the time of arrival in Sardinia, Italy, via the Mediterranean route. In addition, the obtained sequence was compared to the SARS-CoV-2 genomes circulating in Sardinia as well as among African countries in the same period. This aided in the contextualization of the geographical area of arrival and origin, respectively. Furthermore, comparison with other SARS-CoV-2 isolates recently identified in immigrants from the Libyan migration route (8) allowed us to assess similarities with other viral genomes from comparable contexts.

## Methods

### Study Population

On September 19^th^ and 20^th^, 2020, a non-governmental organization (NGO) ship conducted three independent interventions off the coast of Libya and rescued 133 migrants that were embarked at the harbor of Olbia in Eastern Sardinia (Italy) on September 25^th^. Out of the 133 migrants, 116 were male and 17 were female. The age ranged from 5 months to 46 years, and 62 of them (46.6%) were minors. The migrants came from different parts of Africa and Asia, and most of them were native from Guinea, Mali, Libya, and Egypt (Supplementary Table 1).

### Ethical Approval

Samples were collected as part of clinical diagnostics following official procedure (ISS Working group Diagnostics and microbiological surveillance of COVID-19, https://www.iss.it/rapporti-covid-19//asset_publisher/btw1J82wtYzH/content/id/5329985). All samples and data are directly related to Italian pandemic control and were previously anonymized as required by the Italian Data Protection Code (Legislative Decree 196/2003). The general authorizations issued by the Data Protection Authority. Ethics Committee approval was deemed unnecessary because, under Italian law, all sensitive data were deleted and we collected only age, gender, sampling date and demographic information (Art. 6 and Art. 9 of Legislative Decree 211/2003).

### SARS-CoV-2 Detection and Whole Genome Sequencing

The 125 nasopharyngeal swabs were collected from the migrants and tested for SARS-CoV-2 at the regional reference center of Microbiology and Virology Division, Azienda Opedaliera Universitaria (AOU) in Sassari, Italy. Three SARS-CoV-2 specific targets (RdRp/S, E and N) were detected by real-time reverse transcription-polymerase chain reaction (rt-RT-PCR) by using Allplex COVID-19 assay (Seegene, Seoul, Korea) and CFX96™ Real-time PCR Detection System -IVD (BioRad Laboratories Inc, CA, USA).

RNA from 10 SARS-CoV-2 positive swab samples was extracted with STARMag 96 x 4 viral DNA/RNA 200 C kit^*^ (Seegene, Seoul, Korea) and included in the ARTIC Network suggested v2 protocol (1) for viral genome sequencing. For each sample, 5 μl of purified RNA has been used for first-strand cDNA synthesis using SuperScript IV reverse transcriptase (Invitrogen, CA, USA) with modifications according to (9). Briefly, after the denaturation (65°C for 5 min followed by 1 min in ice) of the first mix - containing Random Examer (Invitrogen), dNTPs (Invitrogen), and the RNA - a second mix containing RT Buffer, 100 mM DTT, SuperScript-IV RT Enzyme, RNaseOUT (Invitrogen), and DEPC-treated water (Invitrogen) was added in a final volume of 20 μl. The reaction was incubated at 23°C for 10 min, followed by 52°C for 10 min, and 80°C for 10 min.

The amplification reaction was performed using 5 μl cDNA for each of the two PCR for the primer pools A and B. The mix contained 12.5 μl Q5 high-fidelity DNA polymerase 2× (New England Biolabs), 3,7 μL of 10 μM primer pools A or B from the ARTIC nCoV-2019 V3 panel (IDT, Coralville, IA, USA) and nuclease-free water to a final volume of 25 μl. The first denaturation (98°C for 30 s) was followed by 35 cycles of amplification (98°C for 15 s, 65°C for 5 min). PCR products were then quantified using Qubit dsDNA Broad Range reagent (Invitrogen) on a Tecan Infinite F200Pro plate reader and combined at the same concentration in a single tube. Before library preparation, a cleaning step using 1× AMPure XP beads (Beckman Coulter, Brea, CA, USA) was performed, and the products were eluted in 20 μl of water, quantified by Tecan plate reader and Qubit reagent and diluted at the final concentration of 6 ng/μl.

NexteraFlex (Illumina) library preparation was carried out using 90 ng of purified amplicons. After tagmentation reaction with bead-linked transposome in 25 μl final volume, fragmented amplicons were cleaned-up and amplified by 5 cycles of PCR using specific index adapters for Illumina sequencing (IDT for Illumina Nexter DNA UD Indexes, Illumina). Indexed libraries were purified by 1× AMPure XP beads, eluted in 20 μl of Illumina Resuspension Buffer, and quantified to pool libraries at equal concentrations. Final library pool was assayed on Agilent Bioanalyzer with DNA1000 reagents and quantified with Qubit before sequencing. After denaturation, a final dilution to 9 pM was loaded on a MiSeq flow cell (Illumina) to generate 150-bp paired-end (150PE) reads.

The Illumina® DRAGEN COVID Lineage App has been used to perform Kmer-based detection of SARS-CoV-2, and to align the reads to the reference viral genome, call variants, and generate a consensus genome sequence for each sample.

### Nucleotide Alignments and Structural Characterization

**Figure 1:**
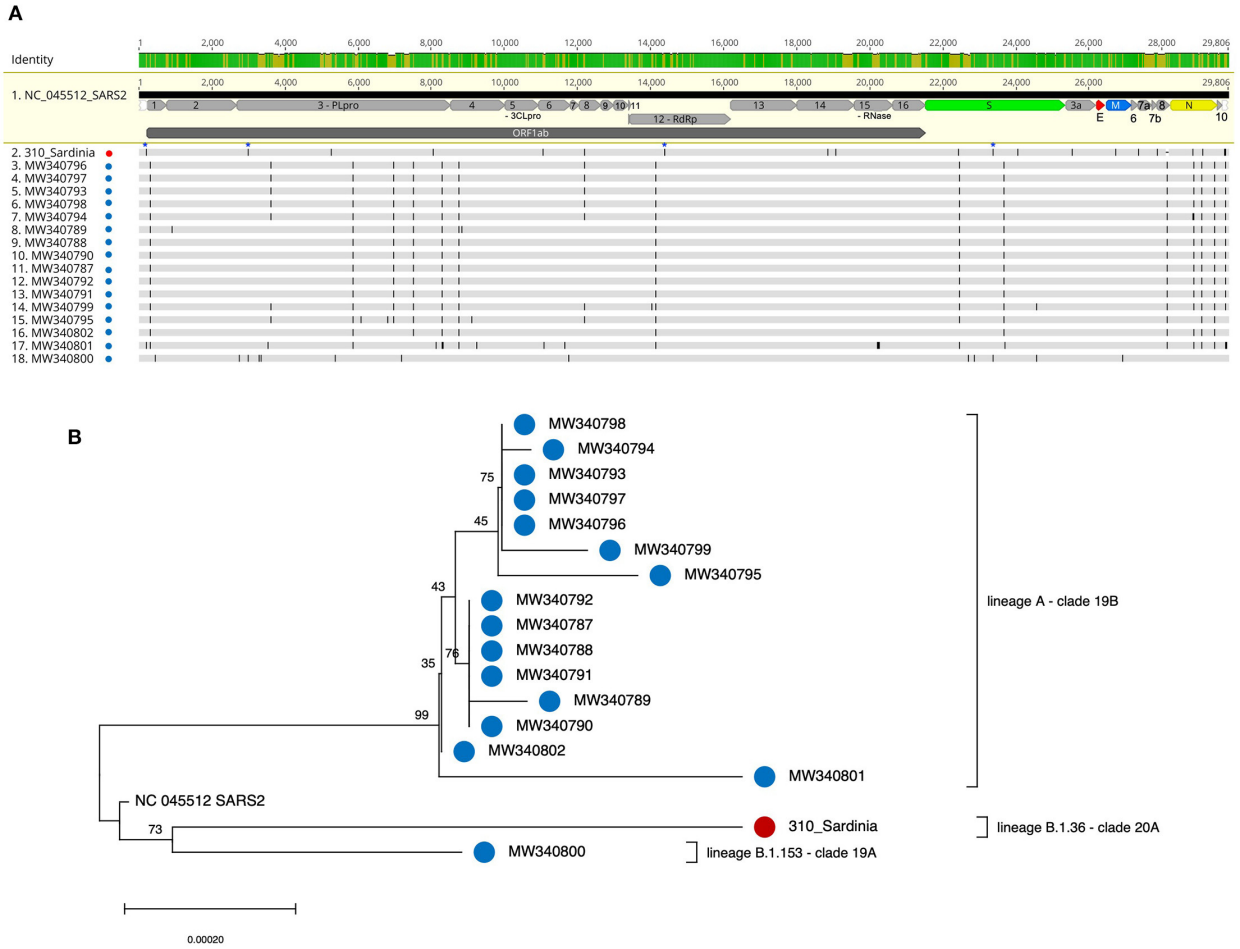
Multiple alignment and phylogenetic tree of SARS-CoV-2 genomes from migrants arrived in Italy through the Libyan route. Viral genomes sequenced from nasopharyngeal swabs of positive migrants arrived at the harbors of Olbia (310 Sardinia NC_OL944327, this study: red dots) and Porto Empedocle (Sicily, (8): blue dots) were used to build a multiple sequence alignment **(A)** that was analyzed with the NJ method **(B)**, applying the pairwise deletion option. Phylogenies were tested with 1000 bootstrap replicates. The SARS-CoV-2 reference sequence (NC_045512) was also included.

**Figure 2:**
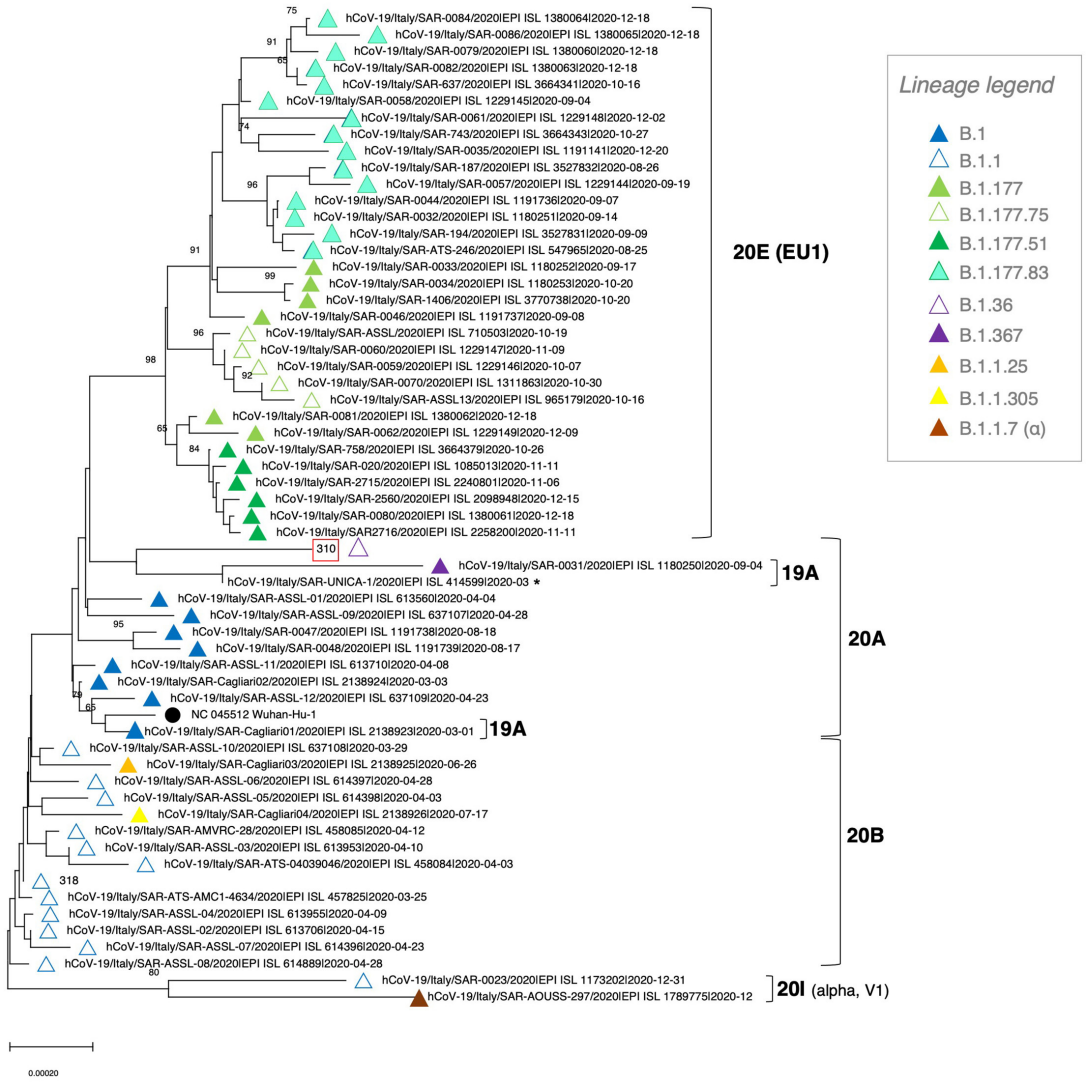
Phylogenetic tree of SARS-CoV-2 genomes circulating in Sardinia in 2020. A total of 57 viral genomes collected in Sardinia during the year were retrieved from GISAID and analyzed with the NJ method, applying the pairwise deletion option. Phylogenies were tested with 100 bootstrap replicates. The SARS-CoV-2 reference sequence (NC_045512) was also included. The color code for SARS-CoV-2 lineages is reported in the legend, while the corresponding viral clades are indicated with squared brackets.

The SARS-CoV-2 complete genomes included in the study have been aligned with respect to SARS-CoV-2 reference isolate Wuhan-Hu-1 (NCBI nucleotide collection, accession number: NC_045512), previously annotated with the positions of viral genes and corresponding proteins. The presence of nucleotide substitutions has been annotated. Viral clade and lineage have been inferred using Nextclade (https://clades.nextstrain.org/) and Pangolin (https://pangolin.cog-uk.io) assigners, respectively. This alignment as well as the ones for phylogenetic analyses have been performed using MAFFT aligner (10) and visualized on Geneious software (11). Particularly, multiple alignments of our sequence to (i) SARS-CoV-2 sequences from Tramuto et al. (Figure 1), and (ii) SARS-CoV-2 sequences circulating in Sardinia during 2020 based on GISAID repository (Figure 2) were performed with the default version of MAFFT aligner; while for the alignment with the SARS-CoV-2 sequences reported in African countries in summer 2020, the –add fragments option has been applied due to their large number.

### Phylogenetic Analyses

**Figure 3:**
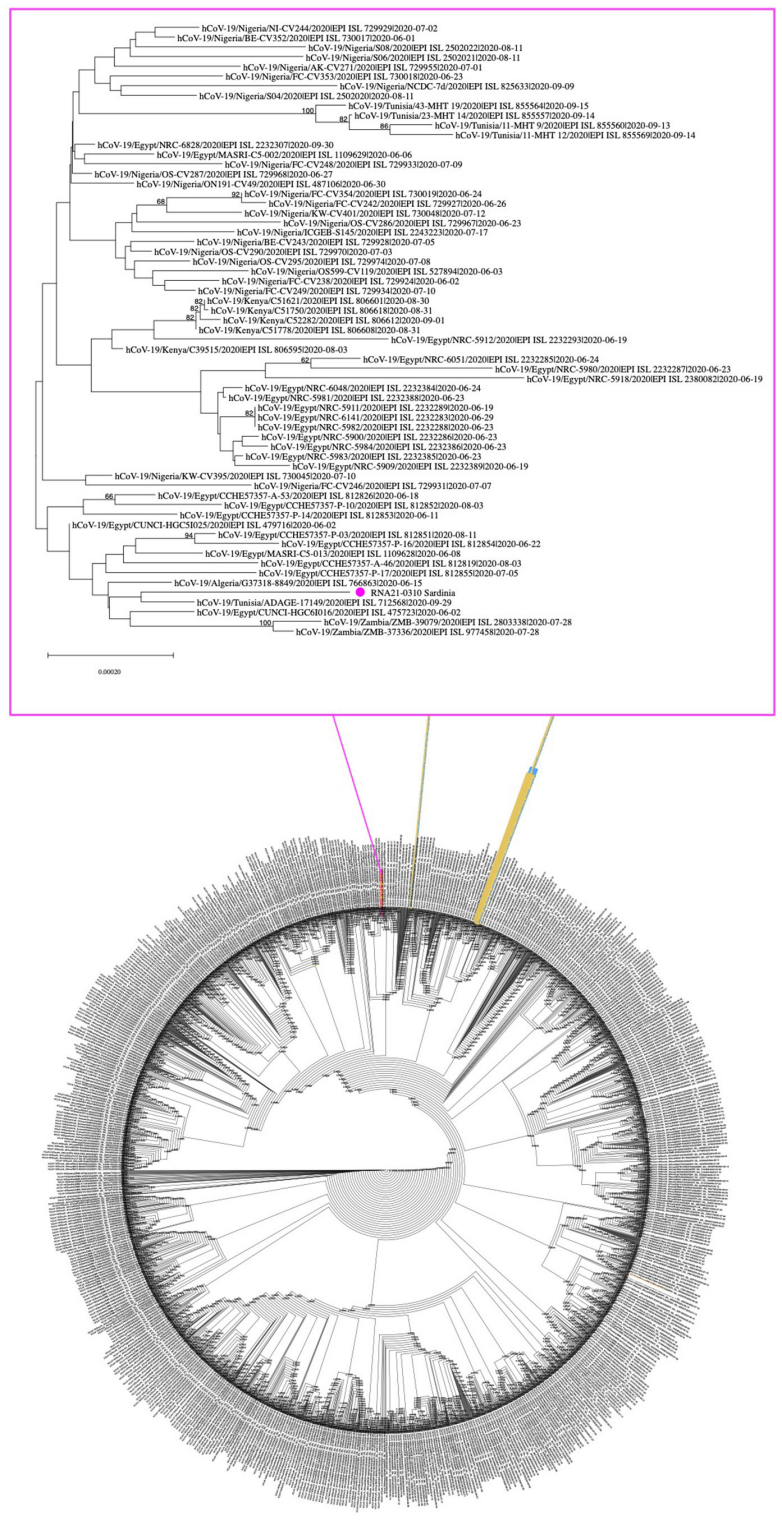
Phylogenetic tree of SARS-CoV-2 genomes circulating in Africa during summer 2020. A total of 5,201 viral genomes collected in African countries between the beginning of June and the end of September 2020 were retrieved from GISAID and analyzed with the NJ method, applying the pairwise deletion option. Phylogenies were tested with 50 bootstrap replicates. The SARS-CoV-2 reference sequence (NC_045512) was also included. The IDs of the 310 sequence (NC_OL944327) from migrant and from (8) are included in yellow tags and highlighted in purple and blue, respectively.

Phylogenetic trees have been built from the above multiple nucleotide alignments using Mega X Software (18) and applying both neighbor-joining and maximum likelihood statistical methods with the pairwise deletion option. Phylogenies were tested by the bootstrap method with either 1,000 (Figure 1), 100 (Figure 2) or 50 (Figure 3) replicates, respectively.

### Retrieval of Publicly Available SARS-CoV-2 Genomes

The 16 SARS-CoV-2 genomes collected from migrants that were rescued along the Libyan route and arrived in Sicily, were retrieved from the recent work by Tramuto and co-workers (8).

The 57 SARS-CoV-2 genomes collected within Sardinia territory in 2020 have been retrieved from GISAID repository (https://www.gisaid.org) applying the following filters: location = Europe/Italy/Sardinia or Europe/Italy/Sardegna, collection dates = from 01/01/2020 to 31/12/2020. Acknowledgments are included in Supplementary Data Sheet 1.

The 5201 SARS-CoV-2 genomes collected in African countries during summer 2020 have been retrieved from GISAID repository (https://www.gisaid.org) applying the following filters: location = Africa, collection dates = from 01/06/2020 to 30/09/2020. Acknowledgments are included in Supplementary Data Sheet 2.

## Results

### Screening for SARS-CoV-2 Infection

A total of 133 migrants of different origins (Supplementary Table 1A) were rescued off the coast of Libya and after a few days, embarked at the port of Olbia in Eastern Sardinia (Italy) in late summer of 2020. Of these, 8 passengers were immediately transferred to a hospital by the Italian authorities for medical reasons, while the remaining 125 migrants were screened for SARS-CoV-2 infection. Real-time reverse transcription-polymerase chain reaction (rt-RT-PCR) of nasopharyngeal swabs identified 10 positive asymptomatic cases out of the 125 migrants. The positive cases were aged from 16 to 37 and showed different geographical origins, arriving mainly from Sudan and Guinea (Supplementary Tables 1, 2). The 10 positive swab samples showing C_T_ values ranging from 26 to 39 cycles (Supplementary Table 2) were included in the ARTIC Network suggested v2 protocol (1) for SARS-CoV-2 genome sequencing.

### Sequencing and Structural Characterization of SARS-CoV-2 Genome Sequences

Next generation sequencing (NGS) of SARS-CoV-2 genome was unsuccessful for samples with C_T_ values >33. Case number 2, from Guinea, had indeed a CT value of 26 and yielded sequence 310 (Supplementary Table 2) which was deposited in Genbank under accession number OL944327.

The nucleotide structure of the above SARS-CoV-2 genome sequence has been characterized with respect to the reference isolate Wuhan-Hu-1 (NCBI nucleotide collection, accession number: NC_045512), previously annotated with the positions of each viral gene and the corresponding Open reading Frames (ORFs) (Table 1; Figure 1A). SARS-CoV-2 genomic sequence 310 showed a remarkable nucleotide diversity as compared to the Wuhan isolate, in line with its belonging to clade 20A - lineage B.1.36. Besides the key nucleotide mutations that have become prevalent in European isolates (241C>T in 5'UTR, 3037C>T synonymous mutation in nsp3, 14408C>T/P4715L in RdRp and 23403A>G/D614G in S, as indicated with a blue star in Figure 1A), sequence 310 had 10 non-synonymous mutations affecting nsp3 (2), nsp6 (1), nsp14 (1), S (1), ORF3a (1), ORF6 (1), ORF8 (1), and N (2) coding sequences; plus 5 synonymous substitution (nsp8, nsp14, S, M, ORF7b) and 2 substitutions in 3′UTR (Table 1; Figure 1A).

**Table 1:** Analysis of 310 SARS-CoV-2 genome substitutions as compared to SARS-CoV-2 reference.

nt position^*^	**Genic portion**	**nt substitution**	**Codon change**	**aa change**
241	5′UTR	C > T	-	-
3037	ORF1AB - nsp3	C > T	UUC > UUT	None
5301	ORF1AB - nsp3	C > T	GCA > GTA	A 1679 V
8097	ORF1AB - nsp3	C > T	ACA > ATA	T 2611 I
11083	ORF1AB - nsp6	G > T	UUG > UUT	L 3606 F
12223	ORF1AB - nsp8	G > T	GUG > GUT	None
14408	ORF1AB - nsp12	C > T	CCU > CTU	**P 314 L**
18877	ORF1AB - nsp14	C > T	CUA > TUA	None
19086	ORF1AB - nsp14	G > T	AAG > AAT	K 1873 N
22444	S	C > T	GAC > GAT	None
23403	S	A > G	GAU > GGU	**D 614 G**
24077	S	G > T	GAU > TAU	D 836 Y
25563	ORF3a	G > T	CAG > CAT	**Q 57 H**
26735	M	C > T	UAC > UAT	None
27382	ORF6	G > T	GAU > TAU	D 61 Y
27884	ORF7b	C > T	GCC > GCT	None
28150-28153	ORF8	- TTAC		Truncation
28854	N	C > T	UCA > UTA	**S 194 L**
29140	N	G > T	CAG > CAT	Q 289 H
29734	3′UTR - stem loop	G > T		-
29736	3′UTR - stem loop	G > C		-

* *Indicated nucleotide positions referred to isolate Wuhan-Hu-1 (NC_045512). For nucleotide substitutions leading to aa changes, the aa position is referred to the correspondent protein sequence. aa substitutions characteristics of the SARS-CoV-2 lineage of belonging of sequence 310 (NC_OL944327) are indicated in bold*.

### Phylogeny and Relationship With Other SARS-CoV-2 Genome Sequences From Migrants

We next compared our SARS-CoV-2 sequence 310 with the viral sequences isolated in a similar socio-sanitary context and reported in the recent work by Tramuto et al., which have been retrieved from 16 positive migrants out of the 210 that were rescued near the Libyan border and arrived at the Porto Empedocle, Sicily, on June 21^st^, 2020 (8). The multiple alignment of these sequences showed immediately that the viral genomes from migrants arriving in Sicily presented several nucleotide substitutions with respect to sequence 310 (Figure 1A). An intermediate situation was represented by isolate MW340800, which had some nucleotide changes in common with sequence 310, among which two of the above key mutations (3037C>T, 23403A>G) (Figure 1A). The NJ phylogenetic tree generated with the multiple alignment (Figure 1B) confirmed that sequence 310 shared a weak phylogenetic relation with isolate MW340800 (bootstrap = 73), even if the rather long branches of the two genomes are indicative of their relatively high nucleotide divergence (Figure 1). In line with this, sequence MW340800 was the only Sicilian isolate belonging to lineage B as well, even though it is of a different clade and lineage (lineage B.1.153 – clade 19A). All the other Sicilian sequences are from an unrelated phylogenetic cluster, in line with their belonging to lineage A (clade 19B) (8) (Figure 1). The same phylogenetic analysis conducted using the maximum likelihood methods gave comparable results (data not shown). Hence, overall, the 17 SARS-CoV-2 genomes analyzed in the tree include 3 different lineages/clades (A/19B, B.1.153/19A, and B.1.36/20A).

### Phylogeny and Relationship With SARS-CoV-2 Genome Sequences From Sardinia

Given that migrants arriving in Italy from the Libyan route come mostly from African countries, we asked whether the so-introduced viral genomes show a remarkable variability as compared to the local, circulating isolates. To this purpose, we retrieved from the GISAID database all SARS-CoV-2 genomes collected in Sardinia in 2020 (*n* = 57, corresponding to the first wave and the first half of the second wave of pandemics) and performed a NJ phylogenetic analysis (Figure 2). Interestingly, sequence 310 even though collected in the second half of the year (September 2020) did not cluster with the isolates circulating in Sardinia in the same period. In fact, these isolates collected between September and December 2020 formed a separate, well-supported phylogenetic group (bootstrap = 98), corresponding to four lineages belonging to 20E clade (B.1.177 and descendant lineages B.1.177.51, B.1.177.75 and B.1.177.83) (Figure 2). Indeed, the analyzed 310 SARS-CoV-2 genome formed an individual branch interspersed with other, unrelated isolates that circulated within the island during the first pandemic wave – i.e., collected in most cases between March and April 2020 – and having in common with them belonging to clade 20A (Figure 2). In more detail, sequence 310 (lineage B.1.36 - clade 20A) clustered near to two sequences from clade 19A: one from the related lineage B.1.367, and another very short of uncertain classification, without showing any phylogenetic relationship. At the bottom of the tree, a GISAID sequence classified as lineage B.1.1 was indeed assigned, together with the only sequence from lineage B.1.1.7 (UK variant of concern -VOC202012/01), to clade 20I. Accordingly, based on GISAID sample info, this sequence has been collected on December 31, 2020, during the first VOC202012/01 outbreak/cluster in Central East Sardinia.

### Phylogeny and Relationship With SARS-CoV-2 Genome Sequences From Africa

In the light of the above phylogenetic divergence of sequence 310 concerning the SARS-CoV-2 genomes circulating in Sardinia in 2020, we then compared them to the viral isolates circulating in Africa in summer 2020 (June to September, *n* = 5201) based on GISAID entries, including also the viral isolates reported in the already mentioned work by Tramuto et al. (8). Due to its dimension, the obtained NJ tree is provided in newik format, while Figure 3 highlights a selected clade extrapolated from the whole tree and including sequence 310, i.e., the genome from migrant N° 2 of Guinea origin (Supplementary Table 2). Sequence 310 clustered in a different part of the tree with respect to the SARS-CoV-2 genomes retrieved from migrants that arrived in Sicily during a similar period (8) (Figure 3). In more detail, sequence 310 was embedded by SARS-CoV-2 sequences from the North part of Africa (mainly Egypt plus some isolates from Algeria and Tunisia), (Figure 3).

## Discussion

The COVID-19 pandemic made it necessary to create an unprecedented block in the movement of people worldwide, in the attempt to limit the spread of the virus. In parallel, migratory movements from low income and/or politically unstable countries never stopped, representing a social phenomenon hard to control in terms of socio-sanitary dynamics and impact on the SARS-CoV-2 epidemiology, especially in the countries where immigrants arrived. In addition, the limited testing capacity and reporting systems in the different African countries greatly affected the availability of information on SARS-CoV-2 diffusion and genetic characteristics in the geographical areas of origin. In the present pilot study, we evaluated the genetic diversity of a SARS-CoV-2 genome (named here 310) that has been isolated from an asymptomatic migrant born in Guinea, who tested positive at the time of arrival in Sardinia via the Mediterranean route, comparing it – on the one side – to the SARS-CoV-2 isolates circulating in Sardinia in 2020 (*n* = 57) and – on the other side – to the viral isolates reported in African countries during the same summer (*n* = 5201).

The nucleotide sequence of the SARS-CoV-2 genome 310 showed a total of 21 nucleotide substitutions as compared to the reference isolate Wuhan-Hu-1 (NCBI nucleotide collection, accession number: NC_045512). Among these, 4 were in common to those shared by clade 20 members, which have become prevalent in most global isolates (Table 1), and of the other 17 mutations, 10 plus were synonymous and 5 were non-synonymous mutations in coding regions. Two substitutions were found in the 3'UTR.

SARS-CoV-2 genome 310 was then compared to the sequences isolated in a similar socio-sanitary context, i.e., the SARS-CoV-2 sequences recently reported by Tramuto et al. from 16 migrants that were rescued near the Libyan border and arrived in Sicily at the end of June 2020 (8) (Figure 1). Also in this case, in contrast to a common time and geographical route of arrival, all the SARS-CoV-2 sequences obtained from migrants that arrived in Sicily (except one) showed substantial nucleotide differences and were included in an independent phylogenetic cluster, in line with their belonging to a different lineage (A) and clade (19B) (Figure 1). Only one sequence showed a higher relationship to the Sardinian sequences and, accordingly, its lineage (B.1.1.53) is the one from which originated later on the descendant lineage of sequence 310 (B.1.36) (Figure 1). Overall, the fact that the 17 SARS-CoV-2 genomes from migrants analyzed in the tree included 3 different lineages (A, B.1.1.53, and B.1.36) and clades (19A, 19B, 20A) further confirms a remarkable nucleotide diversity among the viral isolates circulating along the same migration route, reflecting multiple factors that contribute to SARS-CoV-2 infections with diverse sources of virus preceding embarkation (e.g., country of origin, route of arrival to Libya).

Among the mutations observed in SARS-CoV-2 genome 310, some are known to affect proteins relevant to the virus infectivity and virulence, as the ones in spike and RNA-dependent RNA polymerase (12). In particular, some of the single nucleotide mutations present in the sequence from migrant 310, which are instead absent in Sicilian viral isolates, i.e., S D614G and RdRp/nps12 P314L, had a clinical relevance being associated with increased transmissibility and infectivity (13), and also ORF3a (Q57H) may have a role in the virus immune evasion with potential implications for acquired immunity (14). Other mutations identified only in sequence 310 but not in the Sicilian isolates were in nps6 and nucleocapsid proteins. Nsp6 L3606F mutation has been observed in different European countries and especially in Turkey, one of the countries in the route of migration (15). This substitution might also be relevant for the virulence of SARS-CoV-2, being implicated in limiting autophagosome expansion in different coronavirus, and thus potentially favoring viral infection by limiting the delivery of viral proteins for degradation (16). Q289H mutation in N, occurring in the dimerization domain of the nucleocapsid, has been reported worldwide without however any proved involvement in the virulence of SARS-CoV-2 (17).

We then asked whether the arrival of migrants accounted for the introduction of “new” variants within the Sardinian territory. The comparison with SARS-CoV-2 isolates circulating in Sardinia in 2020 revealed that sequence 310 belonged to clade 20A, which was already present in Sardinia until the first half of the year, in contrast to most local isolates circulating at the time of arrival of case 2, sequence 310. The local circulating sequences were mainly related to clade 20E (Figure 2). Of note, an additional clade (20I) was introduced on the island at the end of the year, including a sequence from the first reported VOC202012/01 outbreak in Central East Sardinia. At the lineage level, sequence 310 belongs to a lineage that, based on the available info, was not reported in Sardinia in 2020 (Figure 2). Accordingly, the B.1.36 report confirmed that this lineage was lowly represented in Italy, having been detected only in two cases from Lazio and Apulia since its first identification, and also worldwide (prevalence <0.5%) (B.1.36 Lineage Report, outbreak.info, accessed 4 October 2021).

According to the belonging to different lineages, sequence 310 shares with Sardinian isolates from this study only 2 of the above mutations (S D614G and RdRp/nps12 P314L) described having a clinical impact and to be stably present in European isolates (12, 13). Hence, in addition to the local evolution of variants, we must also consider a possible external introduction linked to the movement of people, including migration movement which is more difficult to track.

Finally, in the attempt to trace the route of infection until the arrival of the positive migrants in Sardinia, we analyzed sequence 310 in the context of the viral isolates known to be circulating in the different African countries during summer 2020 (Figure 3). Sequence 310 from a migrant from Guinea clustered in a part of the tree near other SARS-CoV-2 genomes from very different geographical areas of Africa (Figure 3) mainly Egypt plus a small number from Algeria and Tunisia, in line with the reported diffusion of lineage B.1.36 as limited to the North part of Africa (B.1.36 Lineage Report, outbreak.info, accessed 4 October 2021). It is hence possible to speculate that the infection could have been acquired after the departure of the migrant from the originating country, along the route to the sites of embarking to Italy, in the North part of Africa. In this regard, migrants' stay in overcrowded Libyan immigrant camps likely accounts for the remarkable viral diversity in COVID-19 clusters that arise in rescue boats. As already pointed out by Tramuto and coworkers, overall, these observations further support the importance of SARS-CoV-2 epidemiological surveillance among migrants and refugees arriving in Italy through the Libyan route, to evaluate the impact on local viral diversity and to gain further insights about the genetic characteristics of the virus in the correspondent countries of origin.

## Data Availability Statement

The datasets presented in this study can be found in online repositories. The names of the repository/repositories and accession number(s) can be found in the article/Supplementary Material.

## Ethics Statement

Ethical review and approval was not required for the study on human participants in accordance with the local legislation and institutional requirements. Written informed consent from the participants' legal guardian/next of kin was not required to participate in this study in accordance with the national legislation and the institutional requirements.

## Author Contributions

NG performed structural and phylogenetic analyses and had a major role in writing the manuscript. BP made a substantial contribution to research design and drafted the paper and revised it critically. RC assessed the RNA quality and performed the next generation sequencing of SARS-CoV-2 genomes. GI and FA contributed to the study design, conducted RT-PCR test, and supervised data analyses. VL and CP performed the initial screening of nasopharyngeal swabs for SARS-Cov-2 and collected information about the migrants that tested positive. ET, CS, SR, and DK coordinated the study and revised the manuscript. All authors participated in the manuscript editing and approved the final version.

## Funding

This work was supported by Sardegna Ricerche agency, grant CarGen4CoV, n. F24I20000190002; by the Li -Ka Shing Foundation, Shantou University Medical College; a Rapid Response award for COVID−19, Canadian Institutes of Health Research (DK and SR); Dalhousie Medical Research Foundation; and a Rapid Response Award from Research Nova Scotia (DK); and a SARS-CoV-2 Genome Canada/Atlantic Genome award (DK); and University of Sassari, grant FAR 2019 (Fondo di Ateneo per la Ricerca 2019) given to SR, grant FAR 2019 (Fondo di Ateneo per la Ricerca 2019) given to CS. DK is the recipient of a Canada Research Chair Award. BP is supported by Sardinian Region [POR-FSE 2014-2020-Asse Prioritario 3 Istruzione e Formazione-Obiettivo Tematico: 10, Priorità d'investimento: 10ii, Obiettivo Specifico: 10.5, Azione dell'Accordo di Partenariato 10.5.12-C.U.P. J86C18000270002].

## Conflict of Interest

The authors declare that the research was conducted in the absence of any commercial or financial relationships that could be construed as a potential conflict of interest.

## Publisher's Note

All claims expressed in this article are solely those of the authors and do not necessarily represent those of their affiliated organizations, or those of the publisher, the editors and the reviewers. Any product that may be evaluated in this article, or claim that may be made by its manufacturer, is not guaranteed or endorsed by the publisher.
