# Supplementary material for: Genomic Snapshot of SARS-CoV-2 in Migrants Entering Through Mediterranean Sea Routes
Source: Front Public Health. 2022 Mar 3;10:846115. doi: 10.3389/fpubh.2022.846115 (PMC8927662; doi:10.3389/fpubh.2022.846115)
Supplement: Supplementary file 1 [file Data_Sheet_1.PDF]

We gratefully acknowledge the following Authors from the Originating laboratories responsible for obtaining the specimens, as well as the Submitting laboratories where the genome data were generated and shared via GISAID, on which this research is based.

All Submitters of data may be contacted directly via www.gisaid.org

Authors are sorted alphabetically.

Accession ID	Originating Laboratory	Submitting Laboratory	Authors
EPI_ISL_733500	1-Laboratory of Microbiology, National Reference Lab, Charles Nicolle Hospital; 2-University of Tunis ElManar, Faculty of Medicine of Tunis, LR99ES09, Tunis, Tunisia	1-Clinical and Experimental Pharmacology Lab, LR16SP02, National Center of Pharmacovigilance, University of Tunis El Manar, Tunis, Tunisia. 2-Neurodegenerative diseases and psychiatric troubles, LR18SP03, Razi Hospital, University of Tunis El Manar, Tunis, Tunisia. 3- Ministry of Health, National Observatory of New and Emerging Diseases, 1006, Tunis, Tunisia	Alia Ben Kahla; Asma Ferjani; Gaies Emna; Guedi Ali Barreh; Habiba Ben Romdhane; Hanen El Jebari; Ilhem Boutiba-Ben Boubaker; Jalila Ben Khelil; Maher Kharrat; Mouna Ben Sassi; Mouna Safer; Nissaf Ben Alaya; Riadh Daghfous; Riadh Gouider.; Salma Abid; Sameh Trabelsi; Sana Ferjani; Sarra Chamman; Souissi Amira
EPI_ISL_640078, EPI_ISL_640107, EPI_ISL_640115, EPI_ISL_640117, EPI_ISL_640127, EPI_ISL_700446, EPI_ISL_700555			
see above	2 Military Hospital wc MAA	NHLS/UCT	Arash Iranzadeh; Bruna Galvao; Carolyn Williamson; Deelan Doolabh; Diana Hardie; Innocent Mudau; Kruger Marais; Lynn Tyers; Marvin Hsiao; Stephen Korsman
EPI_ISL_960151	2 Military Hospital wc MAA	National Health Laboratory Service/UCT	Arash Iranzadeh; Bruna Galvao; Carolyn Williamson; Deelan Doolabh; Diana Hardie; Innocent Mudau; Kruger Marais; Lynn Tyers; Marvin Hsiao; Stephen Korsman
EPI_ISL_602622, EPI_ISL_602623, EPI_ISL_602624, EPI_ISL_602625, EPI_ISL_602626, EPI_ISL_602627, EPI_ISL_602628, EPI_ISL_602629, EPI_ISL_602630, EPI_ISL_602631, EPI_ISL_605784			
see above	AHRI-Sigal	KRISP, KZN Research Innovation and Sequencing Platform	Cele S; Gazy I; Giandhari J; Karim F; Pillay S; Sigal A; Sigl A; Sigla; Tegally H; Wilkinson E; de Oliveira T
EPI_ISL_700521	Alma CDC wc AHC	NHLS/UCT	Arash Iranzadeh; Bruna Galvao; Carolyn Williamson; Deelan Doolabh; Diana Hardie; Innocent Mudau; Kruger Marais; Lynn Tyers; Marvin Hsiao; Stephen Korsman
EPI_ISL_2771497, EPI_ISL_2771498	Arizona State University	Arizona State University	Adam K. Khan; Efrem S. Lim; LaRinda A. Holland; Peter T. Skidmore; Rabia Maqsood
EPI_ISL_700553	Beaufort West CDC wc BWC	NHLS/UCT	Arash Iranzadeh; Bruna Galvao; Carolyn Williamson; Deelan Doolabh; Diana Hardie; Innocent Mudau; Kruger Marais; Lynn Tyers; Marvin Hsiao; Stephen Korsman
EPI_ISL_640031, EPI_ISL_640047, EPI_ISL_700453	Beaufort West Hospital wc BWH	NHLS/UCT	Arash Iranzadeh; Bruna Galvao; Carolyn Williamson; Deelan Doolabh; Diana Hardie; Innocent Mudau; Kruger Marais; Lynn Tyers; Marvin Hsiao; Stephen Korsman
EPI_ISL_527007	Biological Prevention, Army	Biological Prevention, Army	A.E.; A.F.; A.M.; Ageez; B.E.; Elhoseiny; Gad; Harty; M.D.; M.F.; M.G.; Seadawy; Shabaan; Shamel
EPI_ISL_526975, EPI_ISL_526976, EPI_ISL_526977, EPI_ISL_526978, EPI_ISL_526979, EPI_ISL_526980, EPI_ISL_526981, EPI_ISL_526982, EPI_ISL_526983, EPI_ISL_526984, EPI_ISL_526985, EPI_ISL_526986, EPI_ISL_526987, EPI_ISL_526988, EPI_ISL_526989, EPI_ISL_526990, EPI_ISL_526991, EPI_ISL_526992, EPI_ISL_526993, EPI_ISL_526994, EPI_ISL_526995, EPI_ISL_526996, EPI_ISL_526997, EPI_ISL_526998, EPI_ISL_526999, EPI_ISL_527000, EPI_ISL_527001, EPI_ISL_527002, EPI_ISL_527003, EPI_ISL_527004, EPI_ISL_527005, EPI_ISL_527006			
see above	Biological prevention, army	Biological prevention, army	A.E.; A.F.; A.M.; Ageez; B.E.; Elhoseiny; Elhosieny; Gad; Harty; M.D.; M.F.; M.G.; Seadawy; Shabaan; Shamel
EPI_ISL_510526, EPI_ISL_510532	Biological prevention, army	Biological prevention, army	A.A.; A.F.; A.M. and Soliman; Ali; Amer; B.E.; B.S.; ELnabrawy; ElGohary; Elhoseiny; Elhoseny; Elnagdy; Elnakeeb; Gad; H.A.; Harty; Hassan; Kandeil; Karam; M.A.; M.D.; M.F.; M.G.; M.M. and Gad; Raouf; Seadawy; Shamel; T.A.; W.A.; Y.A.; k.E.
EPI_ISL_4026291, EPI_ISL_4026292, EPI_ISL_4026293, EPI_ISL_4026294, EPI_ISL_4026295, EPI_ISL_4026297, EPI_ISL_4026299, EPI_ISL_4026300, EPI_ISL_4026301, EPI_ISL_4026302, EPI_ISL_4026303, EPI_ISL_4026304, EPI_ISL_4026305, EPI_ISL_4026306, EPI_ISL_4026307, EPI_ISL_4026308, EPI_ISL_4026309, EPI_ISL_4026310, EPI_ISL_4026311, EPI_ISL_4026312, EPI_ISL_4026313			
see above	Biorepository and Clinical Virology Laboratory	Northwestern University - Center for Pathogen Genomics and Microbial Evolution	Adeola A. Fowotade; Babafemi O. Taiwo; Egon A. Ozer; Ewean C. Omoruyi; Johnson A. Adeniji; Judd F. Hultquist; Lacy M. Simons; Olubusuyi M. Adewumi; Ramon Lorenzo-Redondo
EPI_ISL_2621085	Bongolethu Clinic wc BLC	NHLS/UCT	Arash Iranzadeh; Bruna Galvao; Carolyn Williamson; Deelan Doolabh; Diana Hardie; Gert Marais; Innocent Mudau; Lynn Tyers; Marvin Hsiao; Stephen Korsman
EPI_ISL_640075	Bothasig CDC wc BLD	NHLS/UCT	Arash Iranzadeh; Bruna Galvao; Carolyn Williamson; Deelan Doolabh; Diana Hardie; Innocent Mudau; Kruger Marais; Lynn Tyers; Marvin Hsiao; Stephen Korsman
EPI_ISL_1516855, EPI_ISL_1516857, EPI_ISL_1516865, EPI_ISL_1516866, EPI_ISL_1516867, EPI_ISL_1516868, EPI_ISL_1516869, EPI_ISL_1516870, EPI_ISL_1516871, EPI_ISL_1516873, EPI_ISL_1516876, EPI_ISL_1516877, EPI_ISL_1516879, EPI_ISL_1516881			
see above	Botswana Harvard HIV Reference Laboratory	Botswana Harvard HIV Reference Laboratory	Boitumelo Zuze; Botshelo Radibe; David Lawrence; Joseph Makhema; Legodile Kooepile; Mosepele Mosepele; Roger Shapiro; Shahin Lockman; Sikhulile Dorcas Maruapula; Simani Gaseitsiwe; Thongbotho Mphoyakgosi; Wonderful T. Choga
EPI_ISL_640036	Bridgeton CDC wc BTC	NHLS/UCT	Arash Iranzadeh; Bruna Galvao; Carolyn Williamson; Deelan Doolabh; Diana Hardie; Innocent Mudau; Kruger Marais; Lynn Tyers; Marvin Hsiao; Stephen Korsman
EPI_ISL_2779396, EPI_ISL_2779397	Busia County Referral Hospital	USAMRD-A, Basic Science Laboratory	Alan Lemtudo; Beth Mutai; Brian Andika; Carol Kifude; Clement Masakwe; Eric Muthanje; Esther Omuseni; Faith Sigei; Gathii Kimita; George Awinda; John Waitumbi; Josphat Nyataya; Rachel Githii; Rehema Liyai; Stephen Ochola
EPI_ISL_2955501, EPI_ISL_2955502, EPI_ISL_2955503, EPI_ISL_2955504, EPI_ISL_2955505	CAPRISA	KRISP, KZn Research Innovation and Sequencing Platform	Emmanuel SJ; Giandhari J; Lessells R; Naidoo Y; Ngcapu S; Pillay S; Ramphal U; Samsunder N; Sivro A; Tegally H; Wilkinson E; de Oliveira T
EPI_ISL_2086873, EPI_ISL_2086874, EPI_ISL_2086875, EPI_ISL_2086876, EPI_ISL_2086877, EPI_ISL_2086878, EPI_ISL_2086879, EPI_ISL_2285327, EPI_ISL_2285328, EPI_ISL_2285329, EPI_ISL_2285330, EPI_ISL_2285331, EPI_ISL_2285332, EPI_ISL_2285333, EPI_ISL_2285334			
see above	CHARLOTTE MAXEKE JOHANNESBURG ACADEMIC HOSPITAL	National Institute for Communicable Diseases of the National Health Laboratory Service	Amoako DG; Bhiman JN; Ismail A; Mahlangu B; Mohale T; Ntuli N; Scheepers C
EPI_ISL_683835	CICM	Malaria Research and Training Center (MRTC-Parasito)	Abdoulaye Djimde; Antoine Dara
EPI_ISL_2683873	CICM, Bamako	Malaria Research and Training Center-Bamako	Abdoul Karim Sangare; Abdoulaye Djimde; Amadou Daou; Antoine Dara; Bourema Kouriba
EPI_ISL_1299497	CMA Dano	Centre Muraz	Abdoul-Salam Ouedraogo; Ange Badjo; Armel Poda; Arsène Somé; Arsène Zongo; Essia Belarbi; Fabian Leendertz; Firmin Kaboré; Grit Schubert; Jasmin Schlotterbeck; Soumeya Ouangraoua; Thérèse Kagone; Yacouba Sawadogo
EPI_ISL_636980	CS Xai Xai	KRISP, KZN Research Innovation and Sequencing Platform	Giandhari J; Ismael N; Nadia Sitoe; Nedio Mabunda; Paulo Arnaldo; Pillay S; Tegally H; Wilkinson E; de Oliveira T
EPI_ISL_576371, EPI_ISL_576372, EPI_ISL_576373	Cancer Biology Department, National Cancer Institute	Cancer Biology Department, National Cancer Institute	A.N.; Abouelhoda, M.; Ahmed; H.K.; Hafez; Hamdy; M.M.; M.S.; O.S.; Soliman; Zekri
EPI_ISL_971451	Cell culture Unit at CV-MIT belonging to HIMMV	Functional Genomic Platform UATRS-biology, CNRST	Abderrazzak Rfak; Amine Idriss Lahlou; Elmostafa EL FAHIME; Hicham Elannaz; Khalid ENNIBI; Marouane MELLOUL; Mly Abdelaziz ELALAOUI; Mostafa ELOUENNASS; Mouhssine Hemlali; Nadia Touil; Sanaa ALAOUI-Amine
EPI_ISL_2232252, EPI_ISL_2232253, EPI_ISL_2232254, EPI_ISL_2232255, EPI_ISL_2232256, EPI_ISL_2232257, EPI_ISL_2232258, EPI_ISL_2232259, EPI_ISL_2232261, EPI_ISL_2232262, EPI_ISL_2232263, EPI_ISL_2232264, EPI_ISL_2232265, EPI_ISL_2232266, EPI_ISL_2232267, EPI_ISL_2232268, EPI_ISL_2232271, EPI_ISL_2232272, EPI_ISL_2232275, EPI_ISL_2232276, EPI_ISL_2232277, EPI_ISL_2232278, EPI_ISL_2232279, EPI_ISL_2232280, EPI_ISL_2232281, EPI_ISL_2232282, EPI_ISL_2232283, EPI_ISL_2232284, EPI_ISL_2232285, EPI_ISL_2232286, EPI_ISL_2232287, EPI_ISL_2232288, EPI_ISL_2232289, EPI_ISL_2232290, EPI_ISL_2232291, EPI_ISL_2232292, EPI_ISL_2232293, EPI_ISL_2232294, EPI_ISL_2232295, EPI_ISL_2232296, EPI_ISL_2232297, EPI_ISL_2232298, EPI_ISL_2232299, EPI_ISL_2232300, EPI_ISL_2232301, EPI_ISL_2232303, EPI_ISL_2232307, EPI_ISL_2232308, EPI_ISL_2232309, EPI_ISL_2232310, EPI_ISL_2232311, EPI_ISL_2232312, EPI_ISL_2232313, EPI_ISL_2232314, EPI_ISL_2232315, EPI_ISL_2232316, EPI_ISL_2232317, EPI_ISL_2232318, EPI_ISL_2232319, EPI_ISL_2232320, EPI_ISL_2232321, EPI_ISL_2232322, EPI_ISL_2232323, EPI_ISL_2232324, EPI_ISL_2232333, EPI_ISL_2232334, EPI_ISL_2232335, EPI_ISL_2232336, EPI_ISL_2232337, EPI_ISL_2232338, EPI_ISL_2232339, EPI_ISL_2232340, EPI_ISL_2232341, EPI_ISL_2232342, EPI_ISL_2232357, EPI_ISL_2232358, EPI_ISL_2232359, EPI_ISL_2232360, EPI_ISL_2232361, EPI_ISL_2232362, EPI_ISL_2232363, EPI_ISL_2232364, EPI_ISL_2232365, EPI_ISL_2232366, EPI_ISL_2232367, EPI_ISL_2232368, EPI_ISL_2232369, EPI_ISL_2232370, EPI_ISL_2232371, EPI_ISL_2232372, EPI_ISL_2232373, EPI_ISL_2232374, EPI_ISL_2232375, EPI_ISL_2232376, EPI_ISL_2232377, EPI_ISL_2232378, EPI_ISL_2232379, EPI_ISL_2232380, EPI_ISL_2232381, EPI_ISL_2232382, EPI_ISL_2232383, EPI_ISL_2232384, EPI_ISL_2232385, EPI_ISL_2232386, EPI_ISL_2232387, EPI_ISL_2232388, EPI_ISL_2232389, EPI_ISL_2232390, EPI_ISL_2232391, EPI_ISL_2232392, EPI_ISL_2232393, EPI_ISL_2232394, EPI_ISL_2232395, EPI_ISL_2232396, EPI_ISL_2232397, EPI_ISL_2232398, EPI_ISL_2232399, EPI_ISL_2232400, EPI_ISL_2232401, EPI_ISL_2232402, EPI_ISL_2232403, EPI_ISL_2232404, EPI_ISL_2380050, EPI_ISL_2380051, EPI_ISL_2380052, EPI_ISL_2380053, EPI_ISL_2380056, EPI_ISL_2380057, EPI_ISL_2380059, EPI_ISL_2380061, EPI_ISL_2380062, EPI_ISL_2380063, EPI_ISL_2380064, EPI_ISL_2380066, EPI_ISL_2380067, EPI_ISL_2380068, EPI_ISL_2380069, EPI_ISL_2380070, EPI_ISL_2380071, EPI_ISL_2380072, EPI_ISL_2380073, EPI_ISL_2380074, EPI_ISL_2380075, EPI_ISL_2380076, EPI_ISL_2380077, EPI_ISL_2380078, EPI_ISL_2380079, EPI_ISL_2380080, EPI_ISL_2380081, EPI_ISL_2380082, EPI_ISL_2380083, EPI_ISL_2380084, EPI_ISL_2380085, EPI_ISL_2380086, EPI_ISL_2380087, EPI_ISL_2380088, EPI_ISL_2380089, EPI_ISL_2380090, EPI_ISL_2380091, EPI_ISL_2380092, EPI_ISL_2380093, EPI_ISL_2380094, EPI_ISL_2380095, EPI_ISL_2380096, EPI_ISL_2380097, EPI_ISL_2380098			
see above	Center of Scientific Excellence for Influenza Viruses (CSEIV), National Research Centre	Center of Scientific Excellence for Influenza Viruses (CSEIV), National Research Centre	Ahmed E Kayed; Ahmed El-Taweel; Ahmed Kandeil; Ahmed Mostafa; Ghazi Kayali; Mahmoud Shehata; Mina Kamel; Mohamed Ahmed Ali; Mohamed El Sayes; Mokhtar Gomaa; Noura M Abo Shama; Omnia Kutkat; Rabeh El-Shesheny; Richard Webby; Sara Mahmoud; Yassmin Moatasim
EPI_ISL_815255	Centogene	Centogene	Krishna Kumar Kandaswamy; Peter Bauer; Vivi Hue-Trang Lieu
EPI_ISL_794597, EPI_ISL_794598, EPI_ISL_794601	Central Laboratories, Egyptian Ministry of Health and Population	Central Laboratories, Egyptian Ministry of Health and Population	A.E.; Ali; El Guindy; El Sayes; El Taweel, A.; El-Shesheny, R.; Gomaa, M.; Kamel; Kandeil, A.; Kayali, G.; Kayed; Khalifa; Kutkat, O.; M.A.; M.K.; M.N.; Mahmoud; Mahrous, N.; Moatasim, Y.; Mostafa, A.; N.M.; Naguib, A.; Roshdy; S.H.; Saleh, M.; Shawky, S.; Shehata, M.; W.H.
EPI_ISL_529032	Central Molecular Microbiology Laboratory and Next Generation Sequencing Reference Laboratory, Clinical and Chemical Pathology Department, Faculty of Medicine, CAIRO UNIVERSITY	Next Generation Sequencing Reference Laboratory, Faculty of Medicine, CAIRO UNIVERSITY and The Center for Genome and Microbiome Research, Faculty of Pharmacy, CAIRO UNIVERSITY	May Abdelfattah; May Sherif Soliman; Ramy Karam Aziz
EPI_ISL_1357603	Centre For Biotechnology Research And Development	THE AFRICA GENOMICS CENTRE AND CONSULTANCY	Adede Hawi; Cecilia Waruhiu; Damaris Matoke-Muhia; George Michuki; John Njuguna; Lilian Kanjau; Ravena Mubichi
EPI_ISL_1357597	Centre For Biotechnology Research And Development	THE AFRICA GENOMICS CENTRE AND CONSULTANCY LIMITED	Adede Hawi; Cecilia Waruhiu; Damaris Matoke-Muhia; George Michuki; John Njuguna; Lilian Kanjau; Ravena Mubichi
EPI_ISL_4255133, EPI_ISL_4255134, EPI_ISL_4255135, EPI_ISL_4255136, EPI_ISL_4255137, EPI_ISL_4255138, EPI_ISL_4255139			
see above	Centre Hospitalier Universitaire de Bogodogo	Africa Centre for Excellence for Genomics of Infectious Diseases (ACEGID), Redeemer's University	A.T.; Abechi; Ajogbasile; Akano; C.A.; C.T.; Eromon; F.V.; Folarin, O.; Happi; I.B.; J.N.; J.U.; K.O.; Kayode; Nosamiefan, I.; Oguzie; Olawoye; Olumade; Oluniyi; P.E.; P.S.; T.J.; Ugwu; Uwanibe
EPI_ISL_4255006, EPI_ISL_4255007, EPI_ISL_4255008, EPI_ISL_4255023, EPI_ISL_4255027, EPI_ISL_4255033	Centre Hospitalier Universitaire de Tengandogo	Africa Centre for Excellence for Genomics of Infectious Diseases (ACEGID), Redeemer's University	A.T.; Abechi; Ajogbasile; Akano; C.A.; C.T.; Eromon; F.V.; Folarin, O.; Happi; I.B.; J.N.; J.U.; K.O.; Kayode; Nosamiefan, I.; Oguzie; Olawoye; Olumade; Oluniyi; P.E.; P.S.; T.J.; Ugwu; Uwanibe
EPI_ISL_1299491, EPI_ISL_1299495	Centre Hospitalo Universitaire Souro Sanou	Centre Muraz	Abdoul-Salam Ouedraogo; Ange Badjo; Armel Poda; Arsène Somé; Arsène Zongo; Essia Belarbi; Fabian Leendertz; Firmin Kaboré; Grit Schubert; Jasmin Schlotterbeck; Soumeya Ouangraoua; Thérèse Kagone; Yacouba Sawadogo
EPI_ISL_788933, EPI_ISL_788934, EPI_ISL_788935, EPI_ISL_788936, EPI_ISL_788937, EPI_ISL_788938	Centre de Recherche et de Formation en Infectiologie Guinée	TransVIHMI, IRD/INSERM/Monpellier University	Abdoul Karim SOUMAH; Abdoulaye TOURE; Ahidjo AYOUBA; Alimou CAMARA; Alpha Kabinet KEITA; Bouna; Bouna Yatassaye; Christelle BUTEL; Eric DELAPORTE; Jean-louis MONEMOU; Joel KOIVOGUI; Kaba KOUROUMA; Laetitia SERRANO; Mamadou Bhoye KEITA; Mamadou Saliou BAH; Mamadou Saliou SOW; Mandiou DIAKITE; Martine PEETERS; Moriba POVOGUI; Penda Malhado DIALLO; Sakoba KEITA
EPI_ISL_2097223, EPI_ISL_2156822, EPI_ISL_2156825	Centre de Recherches Médicales de Lambaréné (CERMEL)	Centre de Recherches Médicales de Lambaréné (CERMEL)	Bertrand Lell and Ayola Akim Adegnika; Georgelin Nguema Ondo; Gédéon Prince Manouana; Jean Bernard Lekana-Douki; Joël-Fleury Djoba Siawaya; Moustapha Nzamba Maloum; Rodrigue Bikangui; Sam O'neilla Oye Bingono; Samira Zoa Assoumou; Srinivas reddy Pallerla; Steffen Borrmann; Thirumalaisamy P. Velavan
EPI_ISL_2968035, EPI_ISL_2968036, EPI_ISL_2968037, EPI_ISL_2968038, EPI_ISL_2968039, EPI_ISL_2968040, EPI_ISL_2968041, EPI_ISL_2968042, EPI_ISL_2968043, EPI_ISL_2968044, EPI_ISL_2968045, EPI_ISL_2968046, EPI_ISL_2968047, EPI_ISL_2968048, EPI_ISL_2968049, EPI_ISL_2968050, EPI_ISL_2968051, EPI_ISL_2968052, EPI_ISL_2968053			
see above	Centre de Virologie des Maladies infectueuses Tropicales	Functional Genomic Platform UATRS-biology, CNRST	; Ahmed REGGAD; ELOUENNASS; Elmostafa EL FAHIME; Hemlali Mouhssine; Hicham EL ANNAZ; Idriss-Amine LAHLOU; Khalid ENNIBI; Khalid ENNIBI Mostafa; Marouane MELLOUL; Mly Abdelaziz ELALAOUI; Mostafa ELOUENNASS; Nadia TOUIL; Rachid ABI; Rida TAGAJDID; Safae ELKOCHRI; Sanaa ALAOUI-Amine; Youssef AKHOUAD; Zohour

			KASMY
EPI_ISL_2351932, EPI_ISL_2398288, EPI_ISL_2398290, EPI_ISL_2501079, EPI_ISL_2501080, EPI_ISL_2501082, EPI_ISL_2501083, EPI_ISL_2501085, EPI_ISL_2501086, EPI_ISL_2502018, EPI_ISL_2502020, EPI_ISL_2502021, EPI_ISL_2502022, EPI_ISL_2502025			
see above	Centre for Human Virology and Genomics, Microbiology Department, Nigerian Institute of Medical Research	Central Research Laboratory, Nigerian Institute of Medical Research	Ayorinde Babatunde James; Azuka Patrick Okwuraiwe; Babatunde Lawal Salako; Bamidele Iwalokun; Chika Kingsley Onwuamah; Grace Oni; Joseph Ojonugwa Shaibu; Josiah Ayoola Isong; Joy Ayoola; Muinah Adenike Fowora; Ngozi Mirabel Otuonye; Nyam Itse Yusuf; Olufemi Samuel Amoo; Phasha-Muchemenye Mmatshepho; Rahaman A. Ahmed; Rosemary Ajuma Audu; Sharon Abimbola; Sola Ajibaye; Uyi Emokpae; Yusuf Jimoh
EPI_ISL_636981	City of Chimoio	KRISP, KZN Research Innovation and Sequencing Platform	Giandhari J; Ismael N; Nadia Sitoe; Nedio Mabunda; Paulo Arnaldo; Pillay S; Tegally H; Wilkinson E; de Oliveira T
EPI_ISL_640068	Clinic-in-Asla	NHLS/UCT	Arash Iranzadeh; Bruna Galvao; Carolyn Williamson; Deelan Doolabh; Diana Hardie; Innocent Mudau; Kruger Marais; Lynn Tyers; Marvin Hsiao; Stephen Korsman
EPI_ISL_640015, EPI_ISL_640118, EPI_ISL_700414, EPI_ISL_700506, EPI_ISL_3207510, EPI_ISL_3957789	Conville CDC wc CVC	NHLS/UCT	Arash Iranzadeh; Bruna Galvao; Carolyn Williamson; Deelan Doolabh; Diana Hardie; Gert Marais; Innocent Mudau; Kruger Marais; Lynn Tyers; Marvin Hsiao; Rageema Joseph; Stephen Korsman
EPI_ISL_640029, EPI_ISL_640048	D'Almeida Clinic wc DAL	NHLS/UCT	Arash Iranzadeh; Bruna Galvao; Carolyn Williamson; Deelan Doolabh; Diana Hardie; Innocent Mudau; Kruger Marais; Lynn Tyers; Marvin Hsiao; Stephen Korsman
EPI_ISL_2779352, EPI_ISL_2779449, EPI_ISL_2779474, EPI_ISL_2779483, EPI_ISL_2779484, EPI_ISL_2779485, EPI_ISL_2779487, EPI_ISL_2779524, EPI_ISL_2779542, EPI_ISL_2779543			
see above	DFMH	USAMRD-A, Basic Science Laboratory	Alan Lemtudo; Beth Mutai; Brian Andika; Carol Kifude; Clement Masakwe; Eric Muthanje; Esther Omuseni; Faith Sigei; Gathii Kimita; George Awinda; John Waitumbi; Josphat Nyataya; Rachel Githii; Rehema Liyai; Stephen Ochola
EPI_ISL_884826, EPI_ISL_884827, EPI_ISL_884828, EPI_ISL_884829, EPI_ISL_884830, EPI_ISL_884831, EPI_ISL_884832, EPI_ISL_884833, EPI_ISL_884834, EPI_ISL_884835, EPI_ISL_884836, EPI_ISL_884837, EPI_ISL_884838, EPI_ISL_884839, EPI_ISL_884840, EPI_ISL_884841, EPI_ISL_884842, EPI_ISL_884843, EPI_ISL_884844, EPI_ISL_884845, EPI_ISL_884846, EPI_ISL_884847, EPI_ISL_884848, EPI_ISL_884849, EPI_ISL_884850, EPI_ISL_884851, EPI_ISL_884852, EPI_ISL_884853, EPI_ISL_884854, EPI_ISL_884855, EPI_ISL_884856			
see above	Department of Biochemistry, Cell and Molecular Biology, West African Centre for Cell Biology of Infectious Pathogens (WACCBIP), University of Ghana	Department of Biochemistry, Cell and Molecular Biology, West African Centre for Cell Biology of Infectious Pathogens (WACCBIP), University of Ghana	A.-K.; A.B.; Abass; Akoriyea; Amenga-Etego; Amoako, E.; Amuzu; Awandare; Bediako, Y.; Boakye; C.M.; D.S.; Diallo; G.A.; J.M.; Kibinge, N.; Kumi-Ansah, F.; L.N.; Magnussen, V.; Mohammed, A.; Morang'a; Ngoi; O.D.; Odoom, T.; Quashie, P.; S.K.; Tapela, K.; Tei-Maya, F.
EPI_ISL_855557, EPI_ISL_855558, EPI_ISL_855559, EPI_ISL_855560, EPI_ISL_855561, EPI_ISL_855562, EPI_ISL_855563, EPI_ISL_855564, EPI_ISL_855565, EPI_ISL_855566, EPI_ISL_855567, EPI_ISL_855568, EPI_ISL_855569, EPI_ISL_855570, EPI_ISL_855571, EPI_ISL_855572			
see above	Department of Virology, Principal Military Hospital of Instruction of Tunis	Bundeswehr Institute of Microbiology	Habiba Naija; Kilian Stoecker; Malena Bestehorn-Willmann; Markus H. Antwerpen; Mathias C. Walter; Roman Wölfel & Mohamed Ben Moussa; Simone Eckstein; Susann Handrick
EPI_ISL_2876349, EPI_ISL_2876350, EPI_ISL_2876351	Division of Medical Virology, National Health Laboratory Service (NHLS), Tygerberg Hospital / Stellenbosch University	Division of Medical Virology, National Health Laboratory Service (NHLS), Tygerberg Hospital / Stellenbosch University	Bronwyn Kleinhans; Gert van Zyl; Susan Engelbrecht; Tania Stander; Tongai Maponga; Wolfgang Preiser
EPI_ISL_3066385, EPI_ISL_3066387, EPI_ISL_3066389, EPI_ISL_3066391, EPI_ISL_3066393	Division of Medical Virology, National Health Laboratory Service (NHLS), Tygerberg Hospital / Stellenbosch University	Division of Medical Virology, Stellenbosch University and NHLS Tygerberg Hospital	Bronwyn Kleinhans; Gert van Zyl; Menzi Nkosi; Susan Engelbrecht; Wolfgang Preiser
EPI_ISL_960160, EPI_ISL_960161	Dr Abdurahman CDC wc DAC	National Health Laboratory Service/UCT	Arash Iranzadeh; Bruna Galvao; Carolyn Williamson; Deelan Doolabh; Diana Hardie; Innocent Mudau; Kruger Marais; Lynn Tyers; Marvin Hsiao; Stephen Korsman
EPI_ISL_640024, EPI_ISL_2621084	Dysselsdorp Clinic wc DDC	NHLS/UCT	Arash Iranzadeh; Bruna Galvao; Carolyn Williamson; Deelan Doolabh; Diana Hardie; Gert Marais; Innocent Mudau; Kruger Marais; Lynn Tyers; Marvin Hsiao; Stephen Korsman
EPI_ISL_2447778, EPI_ISL_2447779, EPI_ISL_2447780, EPI_ISL_2447781, EPI_ISL_2447782, EPI_ISL_2447783, EPI_ISL_2447784, EPI_ISL_2447786			
see above	East London Laboratory	National Institute for Communicable Diseases of the National Health Laboratory Service	Amoako DG; Bhiman JN; Ismail A; Mahlangu B; Mohale T; Ntuli N; Scheepers C
EPI_ISL_712077, EPI_ISL_712078, EPI_ISL_712092	East London NHLS Laboratory, Eastern Cape, South Africa	National Institute for Communicable Diseases of the National Health Laboratory Service	Allam M; Bhiman JN; Ismail A; Mahlangu B; Mohale T; Ntuli N
EPI_ISL_2271972	Edendale Gateway Clinic	National Institute for Communicable Diseases of the National Health Laboratory Service	Amoako DG; Bhiman JN; Ismail A; Mahlangu B; Mohale T; Ntuli N; Scheepers C
EPI_ISL_475723, EPI_ISL_475724, EPI_ISL_477161, EPI_ISL_478672, EPI_ISL_479686, EPI_ISL_479687, EPI_ISL_479688, EPI_ISL_479689, EPI_ISL_479690, EPI_ISL_479691, EPI_ISL_479692, EPI_ISL_479693, EPI_ISL_479694, EPI_ISL_479695, EPI_ISL_479696, EPI_ISL_479697, EPI_ISL_479698, EPI_ISL_479699, EPI_ISL_479700, EPI_ISL_479701, EPI_ISL_479702, EPI_ISL_479703, EPI_ISL_479704, EPI_ISL_479705, EPI_ISL_479706, EPI_ISL_479707, EPI_ISL_479708, EPI_ISL_479709, EPI_ISL_479710, EPI_ISL_479711, EPI_ISL_479712, EPI_ISL_479713, EPI_ISL_479714, EPI_ISL_479715, EPI_ISL_479716, EPI_ISL_479717, EPI_ISL_479718, EPI_ISL_479719, EPI_ISL_479720, EPI_ISL_479721, EPI_ISL_479722, EPI_ISL_479723, EPI_ISL_479724, EPI_ISL_479725, EPI_ISL_479726, EPI_ISL_479727, EPI_ISL_479728, EPI_ISL_479729, EPI_ISL_479730, EPI_ISL_479731, EPI_ISL_479732, EPI_ISL_479733, EPI_ISL_479734, EPI_ISL_479735, EPI_ISL_524426, EPI_ISL_524427, EPI_ISL_529141, EPI_ISL_529142, EPI_ISL_529143, EPI_ISL_529144, EPI_ISL_529145			
see above	Egyptian National Cancer Institute (ENCI)	Egyptian National Cancer Institute (ENCI)	A.A.; A.N.; Abdel Rahman N; Abdel Rahman N.; Abdelhamid, W.; Abouelhoda; Abouelhoda, M.; Ahmed; Ali; Ali, M.; Amer; Bahnassy; Elkhateeb; Elsissy; Ezzelarab; Gad, A.; H.K.; Hafez; Hamdy; Hassan; Hassan, W.; K.E.; Khattab; M.A.; M.H.; M.M.; M.S.; Mahmoud; Mohamed; O.S.; Raouf, A.; S.M.; Samir, M.; Soliman; W.A.; Zekri
EPI_ISL_640064	Ethembeni Clinic [Prieska]	NHLS/UCT	Arash Iranzadeh; Bruna Galvao; Carolyn Williamson; Deelan Doolabh; Diana Hardie; Innocent Mudau; Kruger Marais; Lynn Tyers; Marvin Hsiao; Stephen Korsman
EPI_ISL_640033, EPI_ISL_640116, EPI_ISL_640121, EPI_ISL_640123, EPI_ISL_640124, EPI_ISL_640125	False Bay Hospital wc FBH	NHLS/UCT	Arash Iranzadeh; Bruna Galvao; Carolyn Williamson; Deelan Doolabh; Diana Hardie; Innocent Mudau; Kruger Marais; Lynn Tyers; Marvin Hsiao; Stephen Korsman
EPI_ISL_581455, EPI_ISL_581487, EPI_ISL_581489, EPI_ISL_581490, EPI_ISL_581491, EPI_ISL_581492, EPI_ISL_581493			
see above	Fondation Congolaise pour la recherche medicale (FCRM)	NGS Competence Center Tübingen, Institut für Medizinische Mikrobiologie und Hygiene, Universitätsklinikum Tübingen	Angel Angelov
EPI_ISL_2779422, EPI_ISL_2779423	GK Prison Busia (Matayo)Matayos Prison	USAMRD-A, Basic Science Laboratory	Alan Lemtudo; Beth Mutai; Brian Andika; Carol Kifude; Clement Masakwe; Eric Muthanje; Esther Omuseni; Faith Sigei; Gathii Kimita; George Awinda; John Waitumbi; Josphat Nyataya; Rachel Githii; Rehema Liyai; Stephen Ochola
EPI_ISL_812788, EPI_ISL_812795, EPI_ISL_812796, EPI_ISL_812797, EPI_ISL_812798, EPI_ISL_812802, EPI_ISL_812803, EPI_ISL_812804, EPI_ISL_812805, EPI_ISL_812806, EPI_ISL_812807, EPI_ISL_812808, EPI_ISL_812809, EPI_ISL_812810, EPI_ISL_812811, EPI_ISL_812812, EPI_ISL_812813, EPI_ISL_812814, EPI_ISL_812815, EPI_ISL_812816, EPI_ISL_812817, EPI_ISL_812818, EPI_ISL_812819, EPI_ISL_812820, EPI_ISL_812821, EPI_ISL_812822, EPI_ISL_812823, EPI_ISL_812824, EPI_ISL_812825, EPI_ISL_812826, EPI_ISL_812827, EPI_ISL_812828, EPI_ISL_812829, EPI_ISL_812830, EPI_ISL_812831, EPI_ISL_812832, EPI_ISL_812833, EPI_ISL_812834, EPI_ISL_812835, EPI_ISL_812836, EPI_ISL_812837, EPI_ISL_812838, EPI_ISL_812839, EPI_ISL_812840, EPI_ISL_812841, EPI_ISL_812842, EPI_ISL_812843, EPI_ISL_812844, EPI_ISL_812845, EPI_ISL_812846, EPI_ISL_812847, EPI_ISL_812848, EPI_ISL_812849, EPI_ISL_812850, EPI_ISL_812851, EPI_ISL_812852, EPI_ISL_812853, EPI_ISL_812854, EPI_ISL_812855, EPI_ISL_812856, EPI_ISL_812857, EPI_ISL_812858, EPI_ISL_812859, EPI_ISL_812860, EPI_ISL_812861, EPI_ISL_812862, EPI_ISL_812863, EPI_ISL_812864, EPI_ISL_812865, EPI_ISL_812866, EPI_ISL_812867, EPI_ISL_812868, EPI_ISL_812869, EPI_ISL_812870, EPI_ISL_812871, EPI_ISL_812872			
see above	Genomics Program, Children Cancer Hospital	Genomics Program, Children Cancer Hospital	Abdo, I.; Abouelnaga, S.; Amer, K.; Bakry, U.; Diab, A.; El-Shaqnqery, H.; El-Zayat, M.; Farawyla, H.; Gomaa, C.; Hadad, A.; Halafawy, A.; Hammad, M.; Hassan, R.; Hassan, W.; Hatem, A.; Hussein, S.; Jalal, D.; Magdeldin, S.; Mansour, T.; Monuir, G.; Salah, H.; Samir, O.; Sayed, A.; Soliman, M.; Soliman, S.; shalaby, L.
EPI_ISL_640016, EPI_ISL_640027, EPI_ISL_2802110	George Hospital wc GRH	NHLS/UCT	Arash Iranzadeh; Bruna Galvao; Carolyn Williamson; Deelan Doolabh; Diana Hardie; Gert Marais; Innocent Mudau; Kruger Marais; Lynn Tyers; Marvin Hsiao; Stephen Korsman
EPI_ISL_640049	George Road Sat Clinic wc GWM	NHLS/UCT	Arash Iranzadeh; Bruna Galvao; Carolyn Williamson; Deelan Doolabh; Diana Hardie; Innocent Mudau; Kruger Marais; Lynn Tyers; Marvin Hsiao; Stephen Korsman
EPI_ISL_2621056, EPI_ISL_2621086, EPI_ISL_3207529	Great Brak River Clinic wc GBC	NHLS/UCT	Arash Iranzadeh; Bruna Galvao; Carolyn Williamson; Deelan Doolabh; Diana Hardie; Gert Marais; Innocent Mudau; Lynn Tyers; Marvin Hsiao; Rageema Joseph; Stephen Korsman
EPI_ISL_960149	Great Brak River Clinic wc GBC	National Health Laboratory Service/UCT	Arash Iranzadeh; Bruna Galvao; Carolyn Williamson; Deelan Doolabh; Diana Hardie; Innocent Mudau; Kruger Marais; Lynn Tyers; Marvin Hsiao; Stephen Korsman
EPI_ISL_640066, EPI_ISL_640067, EPI_ISL_640084, EPI_ISL_640111, EPI_ISL_640112, EPI_ISL_640119, EPI_ISL_640120, EPI_ISL_640122, EPI_ISL_640128, EPI_ISL_640129, EPI_ISL_640130, EPI_ISL_640131, EPI_ISL_640132, EPI_ISL_700493, EPI_ISL_700507, EPI_ISL_700534, EPI_ISL_700577, EPI_ISL_1040801, EPI_ISL_1040802, EPI_ISL_1040804, EPI_ISL_1040807, EPI_ISL_1040808, EPI_ISL_1040811, EPI_ISL_1040812, EPI_ISL_1040813, EPI_ISL_1040815, EPI_ISL_1817754, EPI_ISL_2876939, EPI_ISL_3207531, EPI_ISL_3957788			
see above	Groote Schuur Hospital wc GSH	NHLS/UCT	Arash Iranzadeh; Bruna Galvao; Carolyn Williamson; Deelan Doolabh; Diana Hardie; Gert Marais; Innocent Mudau; Kruger Marais; Lynn Tyers; Marvin Hsiao; Rageema Joseph; Stephen Korsman
EPI_ISL_640069, EPI_ISL_640070, EPI_ISL_640071, EPI_ISL_1040805	Guguletu CHC wc GDH	NHLS/UCT	Arash Iranzadeh; Bruna Galvao; Carolyn Williamson; Deelan Doolabh; Diana Hardie; Innocent Mudau; Kruger Marais; Lynn Tyers; Marvin Hsiao; Stephen Korsman
EPI_ISL_960154, EPI_ISL_960155, EPI_ISL_960156, EPI_ISL_960157, EPI_ISL_960158, EPI_ISL_960163, EPI_ISL_960167			
see above	Guguletu CHC wc GDH	National Health Laboratory Service/UCT	Arash Iranzadeh; Bruna Galvao; Carolyn Williamson; Deelan Doolabh; Diana Hardie; Innocent Mudau; Kruger Marais; Lynn Tyers; Marvin Hsiao; Stephen Korsman
EPI_ISL_636975, EPI_ISL_636976, EPI_ISL_636977, EPI_ISL_636978, EPI_ISL_636979	HP Pemba	KRISP, KZN Research Innovation and Sequencing Platform	Giandhari J; Ismael N; Nadia Sitoe; Nedio Mabunda; Paulo Arnaldo; Pillay S; Tegally H; Wilkinson E; de Oliveira T
EPI_ISL_1040809	Hanover Park CHC wc HPH	NHLS/UCT	Arash Iranzadeh; Bruna Galvao; Carolyn Williamson; Deelan Doolabh; Diana Hardie; Innocent Mudau; Kruger Marais; Lynn Tyers; Marvin Hsiao; Stephen Korsman
EPI_ISL_960165	Hanover Park CHC wc HPH	National Health Laboratory Service/UCT	Arash Iranzadeh; Bruna Galvao; Carolyn Williamson; Deelan Doolabh; Diana Hardie; Innocent Mudau; Kruger Marais; Lynn Tyers; Marvin Hsiao; Stephen Korsman
EPI_ISL_640018	Heidelberg Clinic wc HBC	NHLS/UCT	Arash Iranzadeh; Bruna Galvao; Carolyn Williamson; Deelan Doolabh; Diana Hardie; Innocent Mudau; Kruger Marais; Lynn Tyers; Marvin Hsiao; Stephen Korsman
EPI_ISL_960164, EPI_ISL_960168, EPI_ISL_960169, EPI_ISL_960170	Heideveld CDC wc HVP	National Health Laboratory Service/UCT	Arash Iranzadeh; Bruna Galvao; Carolyn Williamson; Deelan Doolabh; Diana Hardie; Innocent Mudau; Kruger Marais; Lynn Tyers; Marvin Hsiao; Stephen Korsman
EPI_ISL_700559, EPI_ISL_1040803, EPI_ISL_1040806, EPI_ISL_1040810, EPI_ISL_3207530	Heideveld Emergency Centre	NHLS/UCT	Arash Iranzadeh; Bruna Galvao; Carolyn Williamson; Deelan Doolabh; Diana Hardie; Gert Marais; Innocent Mudau; Kruger Marais; Lynn Tyers; Marvin Hsiao; Rageema Joseph; Stephen Korsman
EPI_ISL_960153, EPI_ISL_960162, EPI_ISL_960166	Heideveld Emergency Centre	National Health Laboratory Service/UCT	Arash Iranzadeh; Bruna Galvao; Carolyn Williamson; Deelan Doolabh; Diana Hardie; Innocent Mudau; Kruger Marais; Lynn Tyers; Marvin Hsiao; Stephen Korsman
EPI_ISL_2271973, EPI_ISL_2271974	Helen Joseph	National Institute for Communicable Diseases of the National Health Laboratory Service	Amoako DG; Bhiman JN; Ismail A; Mahlangu B; Mohale T; Ntuli N; Scheepers C
EPI_ISL_700565, EPI_ISL_700571	Herbertsdale Sat Clinic wc HBD	NHLS/UCT	Arash Iranzadeh; Bruna Galvao; Carolyn Williamson; Deelan Doolabh; Diana Hardie; Innocent Mudau; Kruger Marais; Lynn Tyers; Marvin Hsiao; Stephen Korsman
EPI_ISL_700558	Hornlee Clinic wc HLC	NHLS/UCT	Arash Iranzadeh; Bruna Galvao; Carolyn Williamson; Deelan Doolabh; Diana Hardie; Innocent Mudau; Kruger Marais; Lynn Tyers; Marvin Hsiao; Stephen Korsman
EPI_ISL_537728, EPI_ISL_561372, EPI_ISL_1716630, EPI_ISL_1716631, EPI_ISL_1716632, EPI_ISL_1716633, EPI_ISL_1716634, EPI_ISL_1716635, EPI_ISL_1716636, EPI_ISL_1716637, EPI_ISL_1716638, EPI_ISL_1716639, EPI_ISL_1716640, EPI_ISL_1716641, EPI_ISL_1716642, EPI_ISL_1716643, EPI_ISL_1841385, EPI_ISL_1841386, EPI_ISL_1841387, EPI_ISL_1841388, EPI_ISL_1841389, EPI_ISL_1841390, EPI_ISL_1841391, EPI_ISL_1939341, EPI_ISL_1939342, EPI_ISL_1939343, EPI_ISL_1939344, EPI_ISL_1939345, EPI_ISL_1939346, EPI_ISL_1939347, EPI_ISL_1939348, EPI_ISL_1939349, EPI_ISL_1939350, EPI_ISL_1939351, EPI_ISL_1939352, EPI_ISL_1939353, EPI_ISL_1939354, EPI_ISL_1939355, EPI_ISL_1939356, EPI_ISL_1939357, EPI_ISL_1939358, EPI_ISL_1939359, EPI_ISL_1939360, EPI_ISL_1939361, EPI_ISL_1939362, EPI_ISL_1939363, EPI_ISL_1939364, EPI_ISL_1939365, EPI_ISL_1939366, EPI_ISL_1939367, EPI_ISL_1939368, EPI_ISL_1939369, EPI_ISL_1939370, EPI_ISL_1939371, EPI_ISL_1939372, EPI_ISL_1939373, EPI_ISL_1939374, EPI_ISL_1939375, EPI_ISL_1939376, EPI_ISL_1939377, EPI_ISL_1939378, EPI_ISL_1939379, EPI_ISL_1939380, EPI_ISL_1939381, EPI_ISL_1939382, EPI_ISL_1939383			
see above	Hospital Universitario de Gran Canaria Dr. Negrín	SeqCOVID-SPAIN consortium/IBV(CSIC)	Ana Bordes Benítez and SeqCOVID-SPAIN consortium; Francisco J. Chamizo López; M. Carmen Pérez González

EPI_ISL_710532	Hôpital Fattouma-Bourguiba de Monastir	Laboratoire des Procédés de Criblage Moléculaire et Cellulaire-Centre de Biotechnologie de Sfax	A. and Masmoudi, S.; Abdelmoulah, F.; Abid, N.; Ajili, F.; Aouni, M.; Ben Ayed, I.; Bensaid, M.; Chtourou, A.; Elargoubi, A.; Fki-berrajah, L.; Gaaloul, I.; Gargouri, S.; Hammami, A.; Kamoun, S.; Karray Hakim, H.; Kharat, N.; Mastouri, M.; Mhalla, S.; Nabli, A.; Rebai; Smeti, I.; Souissi, A.; Stambouli, N.; Turki, M.
EPI_ISL_1630267, EPI_ISL_1630268, EPI_ISL_1630269	IRESSEF	Abbott	Adbou Padane; Ambroise Ahouidi; Aminata Dia; Aminata Mboup; Ana Olivo; Anna julienne selbe Ndiaye; Barbara Harris; Cyrille Diedhiou; Gavin Cloherty; Mary Rodgers; Moustapha Mbow; Nafissatou Leye; Ndeye Diabou Diagne; Papa Alassane Diaw; Souleymane Mboup; Todd Meyer
EPI_ISL_1434443, EPI_ISL_1435763, EPI_ISL_1436817, EPI_ISL_1437749, EPI_ISL_1438551	Institut National d'Hygiène	Unité Mixte Internationale TransVIHMI (UMI 233 IRD – U1175 INSERM - Université de Montpellier) IRD (Institut de recherche pour le développement)	Abla A. KONOU; Adodo SADJI; Ahidjo AYOUBA; Akoélé SILIADIN; Alassane OURO-MEDELI; Amivi EHLAN; Améyo DORKENOO; Anoumou DAGNRA; Christelle BUTEL; Déléma MABA; Eric DELAPORTE; Issaka Maman; Kokou TEGUENI; Laetitia SERRANO; Martine PEETERS; Messanh DOUFFAN; Mireille PRINCE-DAVID; Mounerou SALOU; Sidonie A.M.KAGNISSODE; Sika DOSSIM; Wembo A. HALATOKO
EPI_ISL_1443002, EPI_ISL_1443003	Institut National d'hygiène	"Unité Mixte Internationale TransVIHMI (UMI 233 IRD – U1175 INSERM - Université de Montpellier) IRD (Institut de recherche pour le développement)"	Abla A. KONOU; Adodo SADJI; Ahidjo AYOUBA; Akoélé SILIADIN; Alassane OURO-MEDELI; Amivi EHLAN; Améyo DORKENOO; Anoumou DAGNRA; Christelle BUTEL; Déléma MABA; Eric DELAPORTE; Issaka Maman; Kokou TEGUENI; Laetitia SERRANO; Martine PEETERS; Messanh DOUFFAN; Mireille PRINCE-DAVID; Mounerou SALOU; Sidonie A.M.KAGNISSODE; Sika DOSSIM; Wembo A. HALATOKO
EPI_ISL_1445275, EPI_ISL_2279498	Institut National d’Hygiène (INH)	Unité Mixte Internationale TransVIHMI (UMI 233 IRD – U1175 INSERM - Université de Montpellier)IRD (Institut de recherche pour le développement)	Abla A. KONOU; Adodo SADJI; Ahidjo AYOUBA; Akoélé SILIADIN; Alassane OURO-MEDELI; Amivi EHLAN; Améyo DORKENOO; Anoumou DAGNRA; Christelle BUTEL; Déléma MABA; Eric DELAPORTE; Issaka Maman; Kokou TEGUENI; Laetitia SERRANO; Martine PEETERS; Messanh DOUFFAN; Mireille PRINCE-DAVID; Mounerou SALOU; Sidonie A.M.KAGNISSODE; Sika DOSSIM; Wembo A. HALATOKO
EPI_ISL_485712	Institut Pasteur	Institut Pasteur de Dakar	Amadou Alpha Sall.; Mamadou Malado Jallow; Mamadou diop; Marie Henriette Dior Ndione; Moussa Moise Diagne; Ndongo Dia; Ousmane Faye; Safietou Sanke
EPI_ISL_476148, EPI_ISL_476149, EPI_ISL_476150, EPI_ISL_476151, EPI_ISL_476491, EPI_ISL_476492, EPI_ISL_476493, EPI_ISL_476494, EPI_ISL_476495, EPI_ISL_476497, EPI_ISL_476514, EPI_ISL_476515, EPI_ISL_476516, EPI_ISL_476558, EPI_ISL_476560, EPI_ISL_476562, EPI_ISL_476564, EPI_ISL_476569, EPI_ISL_476570, EPI_ISL_476572, EPI_ISL_476574, EPI_ISL_477141, EPI_ISL_477142, EPI_ISL_477143, EPI_ISL_477144, EPI_ISL_477145, EPI_ISL_477146, EPI_ISL_477147, EPI_ISL_477148, EPI_ISL_477149, EPI_ISL_477150, EPI_ISL_477151, EPI_ISL_477152, EPI_ISL_477153, EPI_ISL_477154, EPI_ISL_477155, EPI_ISL_477156, EPI_ISL_477157, EPI_ISL_477158, EPI_ISL_477159, EPI_ISL_482874, EPI_ISL_482875, EPI_ISL_482876, EPI_ISL_482877, EPI_ISL_482878, EPI_ISL_485635, EPI_ISL_485708, EPI_ISL_485710, EPI_ISL_485711, EPI_ISL_485713, EPI_ISL_485715, EPI_ISL_485716, EPI_ISL_485717			
see above	Institut Pasteur Dakar	Institut Pasteur de Dakar	Amadou Alpha Sall; Amadou Alpha Sall.; Amadou alpha Sall; Mamadou Diop; Mamadou Malado Jallow; Mamadou diop; Mamadou malado Jallow; Marie Henriette Dior Ndione; Moussa Moise; Moussa Moise Diagne; Ndongo Dia; Ousmane Faye; Ousmane faye; Safietou Sanke; Safietou Sankhe; mamadou Diop
EPI_ISL_498240, EPI_ISL_498241, EPI_ISL_498242, EPI_ISL_498243, EPI_ISL_498244, EPI_ISL_498245, EPI_ISL_498246, EPI_ISL_498247, EPI_ISL_498248, EPI_ISL_498249, EPI_ISL_498250, EPI_ISL_498251, EPI_ISL_498252			
see above	Institut Pasteur de Dakar	Institut Pasteur de Dakar	Amadou Alpha Sall.; Mamadou Diop; Mamadou Malado Jallow; Marie Henriette Dior Ndione; Moussa Moise Diagne; Ndongo Dia; Ousmane Faye; Safietou Sankhe Mbengue
EPI_ISL_2245805, EPI_ISL_2245806, EPI_ISL_2245807, EPI_ISL_2245808, EPI_ISL_2245809, EPI_ISL_2245810, EPI_ISL_2245811, EPI_ISL_2245812, EPI_ISL_2245813, EPI_ISL_2245815, EPI_ISL_2245816, EPI_ISL_2245817, EPI_ISL_2245818, EPI_ISL_2245819, EPI_ISL_2245820, EPI_ISL_2245821, EPI_ISL_2245822, EPI_ISL_2245823, EPI_ISL_2245824, EPI_ISL_2245825, EPI_ISL_2245826, EPI_ISL_2245827, EPI_ISL_2245828, EPI_ISL_2245829, EPI_ISL_2245830, EPI_ISL_2245832, EPI_ISL_2245833, EPI_ISL_2245926, EPI_ISL_2245928, EPI_ISL_2245929, EPI_ISL_2245931, EPI_ISL_2245932, EPI_ISL_2245933, EPI_ISL_2245936, EPI_ISL_2245938, EPI_ISL_2245939, EPI_ISL_2245940, EPI_ISL_2245941, EPI_ISL_2245942, EPI_ISL_2245943, EPI_ISL_2245945, EPI_ISL_2245946, EPI_ISL_2245947, EPI_ISL_2245950, EPI_ISL_2245951, EPI_ISL_2245952, EPI_ISL_2245953, EPI_ISL_2245954, EPI_ISL_2245955, EPI_ISL_2245957, EPI_ISL_2245958, EPI_ISL_2245959, EPI_ISL_2245960, EPI_ISL_2245961, EPI_ISL_2245963, EPI_ISL_2245964, EPI_ISL_2245965			
see above	Institut Pasteur de Guinée	Institut Pateur de Dakar	Dia Ndongo; Diagne Moussa Moïse; Diallo Amadou; Diop Mamadou; Faye Ousmane; Grayo Solene; Loucoubar Cheikh; Maimouna Mbanne; Mbengue Safietou Sankhe; Mohamed Kane; Ndiaye Ndack; Sall Amadou Alpha; Tordo Noel
EPI_ISL_476566	Institut pasteur Dakar	Institut Pasteur de Dakar	Amadou Alpha Sall; Mamadou Diop; Moussa Moise Diagne; Ndongo Dia; Ousmane Faye
EPI_ISL_1827861, EPI_ISL_1827864, EPI_ISL_1827865, EPI_ISL_1827866, EPI_ISL_1827867, EPI_ISL_1827868, EPI_ISL_1827869, EPI_ISL_1827870, EPI_ISL_1827874, EPI_ISL_1827875, EPI_ISL_1827876, EPI_ISL_1827877, EPI_ISL_1827878, EPI_ISL_1827879, EPI_ISL_1827881, EPI_ISL_1827882, EPI_ISL_1827883, EPI_ISL_1827884, EPI_ISL_1827889, EPI_ISL_1827890, EPI_ISL_1827894, EPI_ISL_1827898, EPI_ISL_1827899, EPI_ISL_1827900, EPI_ISL_1827901, EPI_ISL_1827902, EPI_ISL_1827903, EPI_ISL_1827906, EPI_ISL_1827907, EPI_ISL_1827910, EPI_ISL_1827911, EPI_ISL_1827912, EPI_ISL_1827914, EPI_ISL_1827915, EPI_ISL_1827916, EPI_ISL_1827918, EPI_ISL_1827919, EPI_ISL_1827928, EPI_ISL_1827929, EPI_ISL_1827930, EPI_ISL_1827931, EPI_ISL_1827932, EPI_ISL_1827934, EPI_ISL_1827936, EPI_ISL_1827938, EPI_ISL_1827939, EPI_ISL_1827942, EPI_ISL_1827946, EPI_ISL_1827948			
see above	Institute for Health Research, Epidemiological Surveillance and Training (IRESSEF)	Abbott Laboratories	Adbou Padane; Ambroise Ahouidi; Aminata Dia; Aminata Mboup; Ana Olivo; Anna julienne selbe Ndiaye; Barbara Harris; Cyrille Diedhiou; Gavin Cloherty; Mary Rodgers; Moustapha Mbow; Nafissatou Leye; Ndeye Diabou Diagne; Papa Alassane Diaw; Souleymane Mboup; Todd Meyer
EPI_ISL_2492649, EPI_ISL_2492777, EPI_ISL_2492827, EPI_ISL_2492846, EPI_ISL_2492921, EPI_ISL_2492942, EPI_ISL_2492951, EPI_ISL_2493013, EPI_ISL_2493018, EPI_ISL_2493020, EPI_ISL_2685956, EPI_ISL_2685958, EPI_ISL_2685959, EPI_ISL_2685960, EPI_ISL_2685961, EPI_ISL_2685962, EPI_ISL_2685982, EPI_ISL_2685983			
see above	Instituto Nacional de Investigação em Saúde	CERI, Centre for Epidemic Response and Innvoation, Stellenbosch University and KRISP, KZN Research Innovation and Sequencing Platform, UKZN.	Afonso P; David K; Emmanuel SJ; Freitas RH; Giandhari J; Inglês L; Lutucuta S; Miranda J; Morais J; Mufinda M; Naidoo Y; Neto Z; Paulo A Carralero RR Paixão JP; Pereira A; Pillay S; Tegally H; Wilkinson E; de Oliveira T
EPI_ISL_1347896, EPI_ISL_1347897, EPI_ISL_1347898	Instituto Nacional de Investigação em Saúde	KRISP, KZN Research Innovation and Sequencing Platform	Afonso P; David K; Emmanuel SJ; Freitas RH; Giandhari J; Inglês L; Lutucuta S; Miranda J; Morais J; Mufinda M; Naidoo Y; Neto Z; Paulo A Carralero RR Paixão JP; Pereira A; Pillay S; Tegally H; Wilkinson E; de Oliveira T
EPI_ISL_887420, EPI_ISL_887421, EPI_ISL_887426, EPI_ISL_887429, EPI_ISL_887431, EPI_ISL_887434, EPI_ISL_887500, EPI_ISL_887501, EPI_ISL_887502, EPI_ISL_887503, EPI_ISL_2396890, EPI_ISL_2396891, EPI_ISL_2396892, EPI_ISL_2396893, EPI_ISL_2396894, EPI_ISL_2396895, EPI_ISL_2396896, EPI_ISL_2396900, EPI_ISL_2396901, EPI_ISL_2396902, EPI_ISL_2396903, EPI_ISL_2396904, EPI_ISL_2396913			
see above	Instituto Nacional de Saude (INS), Mozambique	KRISP, KZN Research Innovation and Sequencing Platform	Emmanuel S; Giandhari J; Nadia Sitoe; Nalia Ismael; Nedio Mabunda; Paulo Arnaldo; Pillay S; Tegally H; Wilkinson E; de Oliveira T
EPI_ISL_1652053, EPI_ISL_1652054	Integrated Biorepository of H3Africa Uganda – IBRH3AU	Molecular Biology Laboratory	Ashaba Fred Katabazi; Bernard Ssentalo Bagaya; David Patrick Kateete; Edgar Kigozi; Emmanuel Nasinghe; Eric Katagirya; Gerald Mboowa; Lwanga Newton; Misaki Wayengera; Moses Joloba; Moses Luutu; Nsubuga Gideon; Rogers Kamulegeya; Samuel Kirimunda; Sarah Stanley; Savannah Mwesigwa
EPI_ISL_4251927, EPI_ISL_4251928, EPI_ISL_4251929, EPI_ISL_4251930, EPI_ISL_4251931, EPI_ISL_4251932, EPI_ISL_4251933, EPI_ISL_4251934, EPI_ISL_4251935, EPI_ISL_4251936, EPI_ISL_4251937, EPI_ISL_4251938, EPI_ISL_4251939, EPI_ISL_4251940, EPI_ISL_4251941, EPI_ISL_4252008, EPI_ISL_4252070, EPI_ISL_4252083, EPI_ISL_4252094, EPI_ISL_4252099, EPI_ISL_4252100, EPI_ISL_4252109, EPI_ISL_4252110, EPI_ISL_4252115, EPI_ISL_4252116, EPI_ISL_4252141, EPI_ISL_4252144, EPI_ISL_4252146, EPI_ISL_4252156, EPI_ISL_4252201, EPI_ISL_4252257, EPI_ISL_4252260, EPI_ISL_4252271, EPI_ISL_4252275, EPI_ISL_4252317, EPI_ISL_4252319, EPI_ISL_4252328, EPI_ISL_4252329, EPI_ISL_4252331, EPI_ISL_4252340, EPI_ISL_4252352, EPI_ISL_4252358, EPI_ISL_4252370, EPI_ISL_4252374, EPI_ISL_4252441, EPI_ISL_4252446, EPI_ISL_4252452, EPI_ISL_4252456, EPI_ISL_4252493, EPI_ISL_4252495, EPI_ISL_4252500, EPI_ISL_4252507, EPI_ISL_4252516, EPI_ISL_4252522, EPI_ISL_4252527, EPI_ISL_4252546			
see above	International Livestock Research Institute	International Livestock Research Institute	Collins Muli; Daniel Ouso; Edward Kiritu; Edward O. Abworo; Gilbert Kibet; Patrick Amoth; Paul Dobi; Samuel O. Oyola; Shebbar Osiany; Sonal P. Henson; Vishvanath Nene
EPI_ISL_1697387, EPI_ISL_1697388, EPI_ISL_1697390, EPI_ISL_1697392, EPI_ISL_1697393, EPI_ISL_1697394, EPI_ISL_1697395, EPI_ISL_1697396			
see above	Iressef / LBV	Iressef Genomics lab	Abdou PADANE; Adjiratou Aissatou BA; Alioune TINE; Ambroise AHOUIDI; Aminata DIA; Aminata MBOUP; Anna Julienne Selbe NDIAYE; Assane DIENG; Astou Gaye GAYE; Barada CISSE; Birahim Piere NDIAYE; Gora LO; Khadim GUEYE; Makhtar Camara; Mengue FALL; Moustapha MBOW; Nafisatou LEYE; Ndeye Coumba Toure KANE; OUMY DIOP; Papa Alassane DIAW; Pauline SENE; SADA DIALLO; Serigne Mbaye Lo NDIAYE; Souleymane MBOUP; Yacine DIA
EPI_ISL_2779373	Isebania Sub County Hospital	USAMRD-A, Basic Science Laboratory	Alan Lemtudo; Beth Mutai; Brian Andika; Carol Kifude; Clement Masakwe; Eric Muthanje; Esther Omuseni; Faith Sigei; Gathii Kimita; George Awinda; John Waitumbi; Josphat Nyataya; Rachel Githii; Rehema Liyai; Stephen Ochola
EPI_ISL_2271952, EPI_ISL_2271953, EPI_ISL_2271955, EPI_ISL_2271956, EPI_ISL_2271957, EPI_ISL_2271964, EPI_ISL_2271965, EPI_ISL_2271971, EPI_ISL_2271977, EPI_ISL_2271978			
see above	Joubert Clinic	National Institute for Communicable Diseases of the National Health Laboratory Service	Amoako DG; Bhiman JN; Ismail A; Mahlangu B; Mohale T; Ntuli N; Scheepers C
EPI_ISL_2779455, EPI_ISL_2779456	KCRH KEMRI/DEID Surveillance Site	USAMRD-A, Basic Science Laboratory	Alan Lemtudo; Beth Mutai; Brian Andika; Carol Kifude; Clement Masakwe; Eric Muthanje; Esther Omuseni; Faith Sigei; Gathii Kimita; George Awinda; John Waitumbi; Josphat Nyataya; Rachel Githii; Rehema Liyai; Stephen Ochola
EPI_ISL_2348630, EPI_ISL_2348631, EPI_ISL_2361365	KEMRI	KEMRI & ICGEB	Alessandro Marcello; Emanuele Orsini; Eric A Lelo; Janet Majanja; Samwel L Symekher; Silvanos M Opanda; Wadegu Meshack; Wallace D Bulimo
EPI_ISL_2602908, EPI_ISL_2602909, EPI_ISL_2602910, EPI_ISL_2602911, EPI_ISL_2602912, EPI_ISL_2602913, EPI_ISL_2602914, EPI_ISL_2602915, EPI_ISL_2602916, EPI_ISL_2602917, EPI_ISL_2602918, EPI_ISL_2602919, EPI_ISL_2602920, EPI_ISL_2602921, EPI_ISL_2602922, EPI_ISL_2602923, EPI_ISL_2602924, EPI_ISL_2602925, EPI_ISL_2602926, EPI_ISL_2602927, EPI_ISL_2602928, EPI_ISL_2602929, EPI_ISL_2602931, EPI_ISL_2602933, EPI_ISL_2602934, EPI_ISL_2602935, EPI_ISL_2602936, EPI_ISL_2602937, EPI_ISL_2602938, EPI_ISL_2602939, EPI_ISL_2602941, EPI_ISL_2602942, EPI_ISL_2602943, EPI_ISL_2602946, EPI_ISL_2602947, EPI_ISL_2602948, EPI_ISL_2602949, EPI_ISL_2602950, EPI_ISL_2602951, EPI_ISL_2602952			
see above	KEMRI-CGHR	KEMRI-Wellcome Trust Research Programme,Kilifi	; Githinji G.; Matoke D.; Mburu M.W.; Mohamed K.S.; Onyango C.; de Laurent Z.
EPI_ISL_3049376, EPI_ISL_3049377, EPI_ISL_3049378, EPI_ISL_3049379, EPI_ISL_3049380, EPI_ISL_3049381, EPI_ISL_3049382, EPI_ISL_3049383, EPI_ISL_3049384, EPI_ISL_3049385, EPI_ISL_3049386, EPI_ISL_3049387, EPI_ISL_3049388, EPI_ISL_3049389, EPI_ISL_3049390, EPI_ISL_3049391, EPI_ISL_3049392, EPI_ISL_3049393, EPI_ISL_3049394, EPI_ISL_3049395, EPI_ISL_3049396, EPI_ISL_3049397, EPI_ISL_3049398, EPI_ISL_3049399, EPI_ISL_3049400, EPI_ISL_3049401, EPI_ISL_3049402, EPI_ISL_3049403, EPI_ISL_3049404, EPI_ISL_3049405, EPI_ISL_3049406, EPI_ISL_3049408, EPI_ISL_3049409, EPI_ISL_3049410, EPI_ISL_3049411, EPI_ISL_3049412, EPI_ISL_3049413, EPI_ISL_3049414, EPI_ISL_3049415, EPI_ISL_3049416, EPI_ISL_3049417, EPI_ISL_3049418, EPI_ISL_3049419, EPI_ISL_3049442, EPI_ISL_3049443, EPI_ISL_3049444, EPI_ISL_3049445, EPI_ISL_3049446, EPI_ISL_3049447, EPI_ISL_3049448, EPI_ISL_3049449, EPI_ISL_3049450, EPI_ISL_3049451, EPI_ISL_3049452, EPI_ISL_3049453, EPI_ISL_3049454, EPI_ISL_3049553, EPI_ISL_3049651, EPI_ISL_3049652, EPI_ISL_3049724, EPI_ISL_3049725			
see above	KEMRI-Wellcome Trust Research Programme,Kilifi	KEMRI-Wellcome Trust Research Programme,Kilifi	Githinji G.; Matoke D.; Mburu M.W.; Mohamed K.S.; Thiongo K.; de Laurent Z.
EPI_ISL_568722, EPI_ISL_568723, EPI_ISL_568725, EPI_ISL_568871, EPI_ISL_568872, EPI_ISL_806548, EPI_ISL_806549, EPI_ISL_806550, EPI_ISL_806551, EPI_ISL_806552, EPI_ISL_806553, EPI_ISL_806554, EPI_ISL_806555, EPI_ISL_806556, EPI_ISL_806557, EPI_ISL_806558, EPI_ISL_806559, EPI_ISL_806560, EPI_ISL_806561, EPI_ISL_806562, EPI_ISL_806563, EPI_ISL_806564, EPI_ISL_806565, EPI_ISL_806566, EPI_ISL_806567, EPI_ISL_806568, EPI_ISL_806569, EPI_ISL_806570, EPI_ISL_806571, EPI_ISL_806572, EPI_ISL_806573, EPI_ISL_806574, EPI_ISL_806575, EPI_ISL_806576, EPI_ISL_806577, EPI_ISL_806578, EPI_ISL_806579, EPI_ISL_806580, EPI_ISL_806581, EPI_ISL_806582, EPI_ISL_806583, EPI_ISL_806584, EPI_ISL_806585, EPI_ISL_806586, EPI_ISL_806587, EPI_ISL_806588, EPI_ISL_806589, EPI_ISL_806590, EPI_ISL_806591, EPI_ISL_806592, EPI_ISL_806593, EPI_ISL_806594, EPI_ISL_806595, EPI_ISL_806596, EPI_ISL_806597, EPI_ISL_806598, EPI_ISL_806599, EPI_ISL_806600, EPI_ISL_806601, EPI_ISL_806602, EPI_ISL_806603, EPI_ISL_806604, EPI_ISL_806605, EPI_ISL_806606, EPI_ISL_806607, EPI_ISL_806608, EPI_ISL_806609, EPI_ISL_806610, EPI_ISL_806611, EPI_ISL_806612, EPI_ISL_806613, EPI_ISL_806614, EPI_ISL_806615, EPI_ISL_806616, EPI_ISL_806617, EPI_ISL_806618, EPI_ISL_806619, EPI_ISL_806620, EPI_ISL_806621, EPI_ISL_806623, EPI_ISL_806624, EPI_ISL_806625, EPI_ISL_806626, EPI_ISL_806627, EPI_ISL_806628, EPI_ISL_806629, EPI_ISL_806630, EPI_ISL_806631, EPI_ISL_806632, EPI_ISL_806633, EPI_ISL_806634, EPI_ISL_806635, EPI_ISL_806636, EPI_ISL_806637, EPI_ISL_806638, EPI_ISL_806639, EPI_ISL_806640, EPI_ISL_806641, EPI_ISL_806642, EPI_ISL_806643, EPI_ISL_806644, EPI_ISL_806645, EPI_ISL_806646, EPI_ISL_806647, EPI_ISL_806648, EPI_ISL_806649, EPI_ISL_806650, EPI_ISL_806651, EPI_ISL_806652, EPI_ISL_806653, EPI_ISL_806654, EPI_ISL_806655, EPI_ISL_806656, EPI_ISL_806657, EPI_ISL_806658, EPI_ISL_806659, EPI_ISL_806660, EPI_ISL_806661, EPI_ISL_806662, EPI_ISL_806663, EPI_ISL_806664, EPI_ISL_806665, EPI_ISL_806666, EPI_ISL_806667, EPI_ISL_806668, EPI_ISL_806669, EPI_ISL_806670, EPI_ISL_806671, EPI_ISL_806672, EPI_ISL_806673, EPI_ISL_806674, EPI_ISL_806675, EPI_ISL_806676, EPI_ISL_806677, EPI_ISL_806678, EPI_ISL_806679, EPI_ISL_806680, EPI_ISL_806681, EPI_ISL_806682, EPI_ISL_806683, EPI_ISL_806684, EPI_ISL_806685, EPI_ISL_806686, EPI_ISL_806687, EPI_ISL_806688, EPI_ISL_806689, EPI_ISL_806690, EPI_ISL_806691, EPI_ISL_806692, EPI_ISL_806693, EPI_ISL_806694, EPI_ISL_806695, EPI_ISL_806696, EPI_ISL_806697, EPI_ISL_806698, EPI_ISL_806699, EPI_ISL_806700, EPI_ISL_806701, EPI_ISL_806702, EPI_ISL_806703, EPI_ISL_806704, EPI_ISL_806705, EPI_ISL_806706, EPI_ISL_806707, EPI_ISL_806708, EPI_ISL_806709, EPI_ISL_806710, EPI_ISL_806711, EPI_ISL_806712, EPI_ISL_806713, EPI_ISL_806714, EPI_ISL_806715, EPI_ISL_806716, EPI_ISL_806717			
see above	KEMRI-Wellcome Trust Research Programme/KEMRI-CGMR-C Kilifi	KEMRI-Wellcome Trust Research Programme/KEMRI-CGMR-C Kilifi	Githinji et al; Githinji et al 2020
EPI_ISL_2779376	KEMRI/DEID Surveillance Site (Mtongwe Navy Base Hospital)	USAMRD-A, Basic Science Laboratory	Alan Lemtudo; Beth Mutai; Brian Andika; Carol Kifude; Clement Masakwe; Eric Muthanje; Esther Omuseni; Faith Sigei; Gathii Kimita; George Awinda; John Waitumbi; Josphat Nyataya; Rachel Githii; Rehema Liyai; Stephen Ochola
EPI_ISL_660664	KRISP, KZN Research Innovation and Sequencing Platform	KRISP, KZN Research Innovation and Sequencing Platform	Giandhari J; Khan S; Lessells R; Mdlalose K; Pillay S; Tegally H; Wilkinson E; York D; de Oliveira T
EPI_ISL_700526	Knysna CDC wc WLC	NHLS/UCT	Arash Iranzadeh; Bruna Galvao; Carolyn Williamson; Deelan Doolabh; Diana Hardie; Innocent Mudau; Kruger Marais; Lynn Tyers; Marvin Hsiao; Stephen Korsman
EPI_ISL_640022, EPI_ISL_640076, EPI_ISL_700501, EPI_ISL_700509	Knysna Hospital wc KNY	NHLS/UCT	Arash Iranzadeh; Bruna Galvao; Carolyn Williamson; Deelan Doolabh; Diana Hardie; Innocent Mudau; Kruger Marais; Lynn Tyers; Marvin Hsiao; Stephen Korsman
EPI_ISL_640030, EPI_ISL_700532	Kwamandlenkosi Clinic wc KMN	NHLS/UCT	Arash Iranzadeh; Bruna Galvao; Carolyn Williamson; Deelan Doolabh; Diana Hardie; Innocent Mudau; Kruger Marais; Lynn Tyers; Marvin Hsiao; Stephen Korsman
EPI_ISL_700495, EPI_ISL_700537	Kwanokuthula CDC wc KWA	NHLS/UCT	Arash Iranzadeh; Bruna Galvao; Carolyn Williamson; Deelan Doolabh; Diana Hardie; Innocent Mudau; Kruger Marais; Lynn Tyers; Marvin Hsiao; Stephen Korsman
EPI_ISL_2779525	LAB-BMC	USAMRD-A, Basic Science Laboratory	Alan Lemtudo; Beth Mutai; Brian Andika; Carol Kifude; Clement Masakwe; Eric Muthanje; Esther Omuseni; Faith Sigei; Gathii Kimita; George Awinda; John Waitumbi; Josphat Nyataya; Rachel Githii; Rehema Liyai; Stephen Ochola
EPI_ISL_2433705, EPI_ISL_2433706, EPI_ISL_2433707, EPI_ISL_2433708, EPI_ISL_2433709, EPI_ISL_2433710, EPI_ISL_2433711, EPI_ISL_2433712, EPI_ISL_2433713, EPI_ISL_2433714, EPI_ISL_2433715, EPI_ISL_2433716, EPI_ISL_2433717, EPI_ISL_2433718, EPI_ISL_2433719, EPI_ISL_2433720, EPI_ISL_2433721, EPI_ISL_2433722, EPI_ISL_2433723, EPI_ISL_2433724, EPI_ISL_2433777, EPI_ISL_2433778, EPI_ISL_2433779, EPI_ISL_2433780, EPI_ISL_2433781, EPI_ISL_2433782, EPI_ISL_2433783, EPI_ISL_2433784, EPI_ISL_2433785, EPI_ISL_2433786, EPI_ISL_2433787, EPI_ISL_2433788, EPI_ISL_2433789, EPI_ISL_2433790, EPI_ISL_2433791, EPI_ISL_2433792, EPI_ISL_2433793, EPI_ISL_2433794, EPI_ISL_2433795, EPI_ISL_2433850, EPI_ISL_2433851, EPI_ISL_2433852, EPI_ISL_2433853, EPI_ISL_2433854, EPI_ISL_2433855, EPI_ISL_2433856, EPI_ISL_2433857, EPI_ISL_2433858, EPI_ISL_2433859, EPI_ISL_2433860, EPI_ISL_2433861, EPI_ISL_2433862, EPI_ISL_2433863, EPI_ISL_2433864, EPI_ISL_2433865, EPI_ISL_2433866, EPI_ISL_2433867, EPI_ISL_2433868, EPI_ISL_2433869, EPI_ISL_2433870, EPI_ISL_2433871, EPI_ISL_2433872, EPI_ISL_2433873, EPI_ISL_2433874, EPI_ISL_2433875, EPI_ISL_2433876, EPI_ISL_2433877, EPI_ISL_2433878, EPI_ISL_2433879, EPI_ISL_2433880, EPI_ISL_2433881, EPI_ISL_2433882, EPI_ISL_2433883, EPI_ISL_2433884, EPI_ISL_2433885, EPI_ISL_2433886, EPI_ISL_2433887, EPI_ISL_2433888, EPI_ISL_2433889, EPI_ISL_2433890, EPI_ISL_2433891, EPI_ISL_2433892, EPI_ISL_2433893, EPI_ISL_2433895, EPI_ISL_2433896, EPI_ISL_2443995, EPI_ISL_2443996			
see above	LANCET, LABORATORIES	National Institute for Communicable Diseases of the National Health Laboratory Service	Amoako DG; Bhiman JN; Ismail A; Mahlangu B; Mohale T; Ntuli N; Scheepers C
EPI_ISL_2318037	Laboratoire Central de Virologie	Laboratoire de Biotechnologie	Abdelomunim Essabbar; Amal Zouaki; Ghizlane EL Amin; Hakima Kabbaj; Lahcen Belyamani and Azeddine Ibrahimi; Mouna Ouadghiri; Myriam Seffar; Saaïd Amzazi; Tarik

			Aanniz
EPI_ISL_2820547, EPI_ISL_2820621, EPI_ISL_2820626	Laboratoire de Diagnostic, Centre de Soins 1, Caisse Nationale de Sécurité Sociale (CNSS) de Djibouti.	IHU Méditerranée Infection.	Anthony Levasseur; Christian A. Devaux.; Didier Raoult; Idil Salah abdillahi; Ikram Omar Osman; Iman Abdillahi Hassan; Jeremy Delerce; Linda Houhamdi; Ludivine Brechar; Mohamed Houmed Aboubaker; Philippe Colson; Pierre-Edouard Fournier; Zeinab Ali Waberi
EPI_ISL_660445, EPI_ISL_660446, EPI_ISL_660447, EPI_ISL_660448, EPI_ISL_660449, EPI_ISL_660453, EPI_ISL_660454, EPI_ISL_660455, EPI_ISL_660456, EPI_ISL_660457, EPI_ISL_660458, EPI_ISL_660459, EPI_ISL_660460, EPI_ISL_660461, EPI_ISL_660462, EPI_ISL_660463, EPI_ISL_660464, EPI_ISL_660465, EPI_ISL_660466, EPI_ISL_660467, EPI_ISL_660469, EPI_ISL_660474, EPI_ISL_660475, EPI_ISL_660476, EPI_ISL_660477, EPI_ISL_660478, EPI_ISL_660479, EPI_ISL_660480, EPI_ISL_660481, EPI_ISL_660482, EPI_ISL_660483, EPI_ISL_660484, EPI_ISL_660485, EPI_ISL_660486, EPI_ISL_660487, EPI_ISL_660490, EPI_ISL_660491, EPI_ISL_660492, EPI_ISL_660493, EPI_ISL_660494, EPI_ISL_660496, EPI_ISL_660498, EPI_ISL_660499, EPI_ISL_660500, EPI_ISL_660501, EPI_ISL_660502, EPI_ISL_660504, EPI_ISL_660505, EPI_ISL_660506, EPI_ISL_660508, EPI_ISL_660509, EPI_ISL_660510, EPI_ISL_660512, EPI_ISL_660513, EPI_ISL_660514, EPI_ISL_660515, EPI_ISL_660516, EPI_ISL_660517, EPI_ISL_660518, EPI_ISL_660520, EPI_ISL_660522, EPI_ISL_660523, EPI_ISL_660524, EPI_ISL_660525, EPI_ISL_660526, EPI_ISL_660527, EPI_ISL_660528			
see above	Laboratoire de Microbiologie CHU Souro Sanou	Centre Muraz	Abdoul-Salam Ouedraogo; Arsène Zongo; Essia Belarbi; Fabian Leendertz; Grit Schubert; Halidou Tinto; Lassana Sangaré; Soumeya Ouangraoua; Yacouba Sawadogo; Zekiba Tarnagda
EPI_ISL_1116468, EPI_ISL_1118675	Laboratoire de Microbiologie- CHU Habib Bourguiba – Sfax	Laboratoire des Procédés de Criblage Moléculaire et Cellulaire-Centre de Biotechnologie de Sfax	A. and Masmoudi, S.; Abdelmoulah, F.; Abid, N.; Ajili, F.; Aouni, M.; Ben Ayed, I.; Bensaid, M.; Chtourou, A.; Elargoubi, A.; Fki-berrajah, L.; Gaaloul, I.; Gargouri, S.; Hammami, A.; Kamoun, S.; Karray Hakim, H.; Kharat, N.; Mastouri, M.; Mhalla, S.; Nabli, A.; Rebai; Smeti, I.; Souissi, A.; Stambouli, N.; Turki, M.
EPI_ISL_712068, EPI_ISL_712568	Laboratoire de Microbiologie- CHU Habib Bourguiba – Sfax adresse	Laboratoire des Procédés de Criblage Moléculaire et Cellulaire-Centre de Biotechnologie de Sfax	A. and Masmoudi, S.; Abdelmoulah, F.; Abid, N.; Ajili, F.; Aouni, M.; Ben Ayed, I.; Bensaid, M.; Chtourou, A.; Elargoubi, A.; Fki-berrajah, L.; Gaaloul, I.; Gargouri, S.; Hammami, A.; Kamoun, S.; Karray Hakim, H.; Kharat, N.; Mastouri, M.; Mhalla, S.; Nabli, A.; Rebai; Smeti, I.; Souissi, A.; Stambouli, N.; Turki, M.
EPI_ISL_933715, EPI_ISL_933719, EPI_ISL_4299860	Laboratoire de Recherche et d'Analyses Medicales de la Gendarmerie Royale	Laboratoire de Recherche et d'Analyses Medicales de la Gendarmerie Royale	Amal SOUIRI; Amal Souiri; Hajar LEMRISS; Mohamed LABIOUI; Mohammed Labioui; Momammed Labioui; Nabil LEMZAOUI; Nabil Lemzaoui; S. El kabbaj; Sanaâ LEMRISS; Sanaâ Lemriss; Saâd EL KABBAJ; Saâd El kabbaj; elmostafa El Fahime
EPI_ISL_3250687	Laboratoire de Recherche et d'Analyses Médicales de la Gendarmerie Royale	Laboratoire de Recherche et d'Analyses Médicales de la Gendarmerie Royale	Amal SOUIRI; Hajar LEMRISS; Sanaâ LEMRISS; Saâd EL KABBAJ
EPI_ISL_953404, EPI_ISL_953405, EPI_ISL_953408, EPI_ISL_953411, EPI_ISL_953412, EPI_ISL_953413, EPI_ISL_953414, EPI_ISL_953415, EPI_ISL_953417, EPI_ISL_953420, EPI_ISL_953422			
see above	Laboratorio de Investigaciones de Baney	"Swiss Tropical and Public Health Institute"	"Carlos Cortes; Bonifacio Manguire Nlavo; Claudia Daubenberger; Diosdado Odjama Nseng Ada; Elizabeth Nyakarungu; Guillermo Garcia; Maximilian Mpina; Mitoha Ondo O Ayekaba; Philip Wonder Phiri"; Salome Hosch; Tobias Schindler
EPI_ISL_1672552, EPI_ISL_1672553, EPI_ISL_1672554, EPI_ISL_1672555, EPI_ISL_1672556, EPI_ISL_1672557	Laboratorio de Investigaciones de Baney	Swiss Tropical and Public Health Institute	Bonifacio Manguire Nlavo; Carlos Cortes; Claudia Daubenberger; Diosdado Odjama Nseng Ada; Elizabeth Nyakarungu; Guillermo Garcia; Maximilian Mpina; Mitoha Ondo O Ayekaba; Philip Wonder Phiri; Salome Hosch; Tobias Schindler
EPI_ISL_648320, EPI_ISL_648322, EPI_ISL_648324, EPI_ISL_648327, EPI_ISL_648328, EPI_ISL_648329, EPI_ISL_648330, EPI_ISL_648337, EPI_ISL_648338, EPI_ISL_648339, EPI_ISL_648356, EPI_ISL_648357, EPI_ISL_648358, EPI_ISL_648359, EPI_ISL_648360, EPI_ISL_648361, EPI_ISL_648362, EPI_ISL_648363, EPI_ISL_648364, EPI_ISL_648365, EPI_ISL_648366, EPI_ISL_648367, EPI_ISL_648368, EPI_ISL_648369, EPI_ISL_648370, EPI_ISL_648371, EPI_ISL_648372, EPI_ISL_648373, EPI_ISL_648374, EPI_ISL_648375, EPI_ISL_648376, EPI_ISL_648377, EPI_ISL_648378, EPI_ISL_648379, EPI_ISL_649157, EPI_ISL_649158, EPI_ISL_649159, EPI_ISL_649160, EPI_ISL_649161, EPI_ISL_649162, EPI_ISL_649163, EPI_ISL_649164, EPI_ISL_649165, EPI_ISL_649166, EPI_ISL_649167, EPI_ISL_649168, EPI_ISL_649169, EPI_ISL_649170, EPI_ISL_649171, EPI_ISL_649172			
see above	Laboratorio de Investigaciones de Baney	University Hospital Basel, Clinical Bacteriology	Adrian Egli; Alfredo Mari; Bonifacio Manguire Nlavo; Carlos Cortes; Claudia Daubenberger; Diosdado Odjama Nseng Ada; Elizabeth Nyakarungu; Guillermo Garcia; Helena Seth-Smith; Madlen Stange; Maximilian Mpina; Mitoha Ondo O Ayekaba; Philip Wonder Phiri; Salome Hosch; Tim Roloff; Tobias Schindler
EPI_ISL_700466	Ladismith (Nissenville) Clinic wc LAF	NHLS/UCT	Arash Iranzadeh; Bruna Galvao; Carolyn Williamson; Deelan Doolabh; Diana Hardie; Innocent Mudau; Kruger Marais; Lynn Tyers; Marvin Hsiao; Stephen Korsman
EPI_ISL_622943, EPI_ISL_622946, EPI_ISL_622947, EPI_ISL_622948, EPI_ISL_622949, EPI_ISL_622950, EPI_ISL_622951, EPI_ISL_622952, EPI_ISL_622953, EPI_ISL_622955, EPI_ISL_622957, EPI_ISL_622959, EPI_ISL_622960, EPI_ISL_622961, EPI_ISL_622962, EPI_ISL_622963, EPI_ISL_622964, EPI_ISL_622965, EPI_ISL_622966, EPI_ISL_622967, EPI_ISL_622968, EPI_ISL_622969, EPI_ISL_622978, EPI_ISL_622983, EPI_ISL_622985, EPI_ISL_622986, EPI_ISL_622988, EPI_ISL_622990, EPI_ISL_622994, EPI_ISL_623064, EPI_ISL_623068, EPI_ISL_623073			
see above	Lancet Laboratories	National Institute for Communicable Diseases of the National Health Laboratory Service	Allam M; Bhiman JN; Ismail A; Khumalo Z; Kwenda S; Mnyameni F; Mohale T; Mtshali P; Subramoney K
EPI_ISL_2779450, EPI_ISL_2779451, EPI_ISL_2779452, EPI_ISL_2779481, EPI_ISL_2779482, EPI_ISL_2779486, EPI_ISL_2779536, EPI_ISL_2779537, EPI_ISL_2779538, EPI_ISL_2779539, EPI_ISL_2779540, EPI_ISL_2779541			
see above	MOH-HQ	USAMRD-A, Basic Science Laboratory	Alan Lemtudo; Beth Mutai; Brian Andika; Carol Kifude; Clement Masakwe; Eric Muthanje; Esther Omuseni; Faith Sigei; Gathii Kimita; George Awinda; John Waitumbi; Josphat Nyataya; Rachel Githii; Rehema Liyai; Stephen Ochola
EPI_ISL_2779522, EPI_ISL_2779523	MOH-HQS	USAMRD-A, Basic Science Laboratory	Alan Lemtudo; Beth Mutai; Brian Andika; Carol Kifude; Clement Masakwe; Eric Muthanje; Esther Omuseni; Faith Sigei; Gathii Kimita; George Awinda; John Waitumbi; Josphat Nyataya; Rachel Githii; Rehema Liyai; Stephen Ochola
EPI_ISL_2779350, EPI_ISL_2779351, EPI_ISL_2779398, EPI_ISL_2779399, EPI_ISL_2779434, EPI_ISL_2779435, EPI_ISL_2779436, EPI_ISL_2779437, EPI_ISL_2779438, EPI_ISL_2779439, EPI_ISL_2779440, EPI_ISL_2779441, EPI_ISL_2779442, EPI_ISL_2779443, EPI_ISL_2779444, EPI_ISL_2779445, EPI_ISL_2779446, EPI_ISL_2779447, EPI_ISL_2779448, EPI_ISL_2779453, EPI_ISL_2779454, EPI_ISL_2779467, EPI_ISL_2779468, EPI_ISL_2779469, EPI_ISL_2779470, EPI_ISL_2779471, EPI_ISL_2779472, EPI_ISL_2779473, EPI_ISL_2779475, EPI_ISL_2779476, EPI_ISL_2779549, EPI_ISL_2779550			
see above	MOH-K	USAMRD-A, Basic Science Laboratory	Alan Lemtudo; Beth Mutai; Brian Andika; Carol Kifude; Clement Masakwe; Eric Muthanje; Esther Omuseni; Faith Sigei; Gathii Kimita; George Awinda; John Waitumbi; Josphat Nyataya; Rachel Githii; Rehema Liyai; Stephen Ochola
EPI_ISL_2346389, EPI_ISL_2346390, EPI_ISL_2346391, EPI_ISL_2346392, EPI_ISL_2346393, EPI_ISL_2346394, EPI_ISL_2346395, EPI_ISL_2346396, EPI_ISL_2346397, EPI_ISL_2346398			
see above	MRC/UVRI & LSHTM Uganda Research Unit, Central Public Health Laboratories	MRC/UVRI & LSHTM Uganda Research Unit, Central Public Health Laboratories	Dan Lule Bugembe; Isaac Sseeewanyana; Matthew Cotten; My V.T. Phan; Patrick Semanda; Pontiano Kaleebu; Susan Nabadda
EPI_ISL_954228, EPI_ISL_954231, EPI_ISL_954232, EPI_ISL_954233, EPI_ISL_954234, EPI_ISL_954235, EPI_ISL_954236, EPI_ISL_954237, EPI_ISL_954238, EPI_ISL_954239, EPI_ISL_954240, EPI_ISL_954241, EPI_ISL_954242, EPI_ISL_954243, EPI_ISL_954244, EPI_ISL_954245, EPI_ISL_954246, EPI_ISL_954247, EPI_ISL_954248, EPI_ISL_954249, EPI_ISL_954250, EPI_ISL_954251, EPI_ISL_954252, EPI_ISL_954253, EPI_ISL_954254, EPI_ISL_1469326, EPI_ISL_1469327, EPI_ISL_1469328, EPI_ISL_1469329, EPI_ISL_1469330, EPI_ISL_1469331, EPI_ISL_1469348, EPI_ISL_1469349, EPI_ISL_1469350, EPI_ISL_1469351, EPI_ISL_1469352			
see above	MRC/UVRI & LSHTM Uganda Research Unit	Where sequence data have been generated and submitted to GISAID	Dan Lule Bugembe; Isaac Sseeewanyana; Matthew Cotten; My V.T. Phan; Patrick Semanda; Pontiano Kaleebu; Susan Nabadda
EPI_ISL_2348511	MRC/UVRI & LSHTM Uganda Research Unit, Central Public Health Laboratories	MRC/UVRI & LSHTM Uganda Research Unit, Central Public Health Laboratories	Dan Lule Bugembe; Isaac Sseeewanyana; Matthew Cotten; My V.T. Phan; Patrick Semanda; Pontiano Kaleebu; Susan Nabadda
EPI_ISL_3152078, EPI_ISL_3152079, EPI_ISL_3152080, EPI_ISL_3152081, EPI_ISL_3152083, EPI_ISL_3152084, EPI_ISL_3152086, EPI_ISL_3152087			
see above	MRCG	LBV Le Dantec	; Abdoulie Kante; Abdul Karim Cesay; Adjiratou Aissatou BA; Aminata Sileymane Thiam; Anna julienne Selbe NDiaye; Assane Dieng; Awa Ba-Diallo; Dianke Samaté; Gora Lo; Halimatou Diop Ndiaye; Jarra Manneh; Khadim Gueye; Makhtar Camara; Mbengué Fall; Moustapha Sakho; Oumy DIOP; Pasacaline Manga; Pauline Yacine Sene; Sada Diallo; Serigne Saliou Niane; ousseynou Gueye
EPI_ISL_560992, EPI_ISL_560993, EPI_ISL_560994, EPI_ISL_560995, EPI_ISL_560996, EPI_ISL_560997, EPI_ISL_560998, EPI_ISL_560999, EPI_ISL_561000, EPI_ISL_561001, EPI_ISL_561002, EPI_ISL_561003, EPI_ISL_561004, EPI_ISL_561005, EPI_ISL_561006, EPI_ISL_561007, EPI_ISL_561008, EPI_ISL_561009, EPI_ISL_561010, EPI_ISL_561011, EPI_ISL_561012, EPI_ISL_561013, EPI_ISL_561025, EPI_ISL_561036, EPI_ISL_561037, EPI_ISL_561038, EPI_ISL_561039, EPI_ISL_561040, EPI_ISL_561041, EPI_ISL_561042, EPI_ISL_561043, EPI_ISL_561044, EPI_ISL_561045, EPI_ISL_561046, EPI_ISL_561047, EPI_ISL_561048, EPI_ISL_561049, EPI_ISL_561050, EPI_ISL_561051, EPI_ISL_561052, EPI_ISL_561053, EPI_ISL_561054, EPI_ISL_561055, EPI_ISL_561056, EPI_ISL_561057, EPI_ISL_561058, EPI_ISL_561059, EPI_ISL_561060, EPI_ISL_561061, EPI_ISL_561062, EPI_ISL_561063, EPI_ISL_561064, EPI_ISL_561065, EPI_ISL_561066, EPI_ISL_561067, EPI_ISL_561068, EPI_ISL_561069, EPI_ISL_561070, EPI_ISL_561071, EPI_ISL_561072, EPI_ISL_561073, EPI_ISL_561074, EPI_ISL_561075, EPI_ISL_561076, EPI_ISL_561077, EPI_ISL_561078, EPI_ISL_561079, EPI_ISL_561080, EPI_ISL_561081, EPI_ISL_561082, EPI_ISL_561083, EPI_ISL_561084, EPI_ISL_561085, EPI_ISL_561086, EPI_ISL_561087, EPI_ISL_561088, EPI_ISL_561089, EPI_ISL_561090, EPI_ISL_561091, EPI_ISL_561092, EPI_ISL_561093, EPI_ISL_561094, EPI_ISL_561095, EPI_ISL_561096, EPI_ISL_561097, EPI_ISL_561098, EPI_ISL_561099, EPI_ISL_561100, EPI_ISL_561101, EPI_ISL_561102, EPI_ISL_561103, EPI_ISL_561104, EPI_ISL_561105, EPI_ISL_561106, EPI_ISL_561107, EPI_ISL_561108, EPI_ISL_561109, EPI_ISL_561110, EPI_ISL_561111, EPI_ISL_561112, EPI_ISL_561113, EPI_ISL_561114, EPI_ISL_561115, EPI_ISL_561116, EPI_ISL_561117, EPI_ISL_561118, EPI_ISL_561119, EPI_ISL_561120, EPI_ISL_561121, EPI_ISL_561122, EPI_ISL_561123, EPI_ISL_561124, EPI_ISL_561125, EPI_ISL_561126, EPI_ISL_561127, EPI_ISL_561128, EPI_ISL_561129, EPI_ISL_561130, EPI_ISL_561131, EPI_ISL_561132, EPI_ISL_561133, EPI_ISL_561134, EPI_ISL_561135, EPI_ISL_561136, EPI_ISL_561137, EPI_ISL_561138, EPI_ISL_561139, EPI_ISL_561140, EPI_ISL_561141, EPI_ISL_561142, EPI_ISL_561143, EPI_ISL_561144, EPI_ISL_561145, EPI_ISL_561146, EPI_ISL_561147, EPI_ISL_561148, EPI_ISL_561149, EPI_ISL_561150, EPI_ISL_561151, EPI_ISL_561152, EPI_ISL_561153, EPI_ISL_561154, EPI_ISL_561155, EPI_ISL_561156, EPI_ISL_561157, EPI_ISL_561158, EPI_ISL_561159, EPI_ISL_561160, EPI_ISL_561161, EPI_ISL_561162, EPI_ISL_561163, EPI_ISL_561164, EPI_ISL_561165, EPI_ISL_561166, EPI_ISL_561167, EPI_ISL_561168, EPI_ISL_561169, EPI_ISL_561170, EPI_ISL_561171, EPI_ISL_561172, EPI_ISL_561173, EPI_ISL_561174, EPI_ISL_561175, EPI_ISL_561176, EPI_ISL_561177, EPI_ISL_561178, EPI_ISL_561179, EPI_ISL_561180, EPI_ISL_561181, EPI_ISL_561182, EPI_ISL_561183, EPI_ISL_561184, EPI_ISL_561185, EPI_ISL_561186, EPI_ISL_561187, EPI_ISL_561188, EPI_ISL_561189, EPI_ISL_561190, EPI_ISL_561191, EPI_ISL_561192, EPI_ISL_561193, EPI_ISL_561194, EPI_ISL_561195, EPI_ISL_561196, EPI_ISL_561197, EPI_ISL_561198, EPI_ISL_561199, EPI_ISL_561200, EPI_ISL_561201, EPI_ISL_561202, EPI_ISL_561203, EPI_ISL_561204, EPI_ISL_561207, EPI_ISL_561209, EPI_ISL_561210, EPI_ISL_561211, EPI_ISL_561212, EPI_ISL_561213, EPI_ISL_561214, EPI_ISL_561215, EPI_ISL_561216, EPI_ISL_561217, EPI_ISL_561218, EPI_ISL_561219, EPI_ISL_561220, EPI_ISL_561221, EPI_ISL_561222, EPI_ISL_561223, EPI_ISL_561224, EPI_ISL_561225, EPI_ISL_561226, EPI_ISL_561227, EPI_ISL_561228, EPI_ISL_561229, EPI_ISL_561230, EPI_ISL_561234, EPI_ISL_561235, EPI_ISL_561236, EPI_ISL_561237, EPI_ISL_561238, EPI_ISL_561240, EPI_ISL_561241, EPI_ISL_561249, EPI_ISL_561252, EPI_ISL_561253, EPI_ISL_561255, EPI_ISL_561256, EPI_ISL_561258, EPI_ISL_561259, EPI_ISL_561260, EPI_ISL_561261, EPI_ISL_561262, EPI_ISL_561263, EPI_ISL_561264, EPI_ISL_561265, EPI_ISL_561266, EPI_ISL_561267, EPI_ISL_561268, EPI_ISL_561269, EPI_ISL_561272, EPI_ISL_561274, EPI_ISL_561276, EPI_ISL_561281, EPI_ISL_561283, EPI_ISL_561285, EPI_ISL_561288, EPI_ISL_561289, EPI_ISL_561290, EPI_ISL_561291, EPI_ISL_561292, EPI_ISL_561293, EPI_ISL_561294, EPI_ISL_561295, EPI_ISL_561297, EPI_ISL_561302, EPI_ISL_561304, EPI_ISL_561309, EPI_ISL_561310, EPI_ISL_561312, EPI_ISL_561315, EPI_ISL_561316, EPI_ISL_561319, EPI_ISL_561322, EPI_ISL_561325, EPI_ISL_561328, EPI_ISL_810971, EPI_ISL_810972, EPI_ISL_810973, EPI_ISL_810974, EPI_ISL_810975, EPI_ISL_810976, EPI_ISL_810977, EPI_ISL_810978, EPI_ISL_810979, EPI_ISL_810980, EPI_ISL_810983, EPI_ISL_810984, EPI_ISL_810985, EPI_ISL_810987, EPI_ISL_810988, EPI_ISL_810989, EPI_ISL_810990, EPI_ISL_810991, EPI_ISL_810992, EPI_ISL_810993, EPI_ISL_810994, EPI_ISL_810995, EPI_ISL_810996, EPI_ISL_810997, EPI_ISL_810998, EPI_ISL_810999, EPI_ISL_811000, EPI_ISL_811001, EPI_ISL_811002, EPI_ISL_811003, EPI_ISL_811004, EPI_ISL_811005, EPI_ISL_811006, EPI_ISL_811007, EPI_ISL_811008, EPI_ISL_811009, EPI_ISL_811010, EPI_ISL_811012, EPI_ISL_811013, EPI_ISL_811014, EPI_ISL_811015, EPI_ISL_811016, EPI_ISL_811017, EPI_ISL_811018, EPI_ISL_811019, EPI_ISL_811020, EPI_ISL_811021, EPI_ISL_811022, EPI_ISL_811023, EPI_ISL_811024, EPI_ISL_811025, EPI_ISL_811026, EPI_ISL_811027, EPI_ISL_811028, EPI_ISL_811029, EPI_ISL_811030, EPI_ISL_811031, EPI_ISL_811032, EPI_ISL_811033, EPI_ISL_811034, EPI_ISL_811035, EPI_ISL_811036, EPI_ISL_1216081, EPI_ISL_1216083, EPI_ISL_1216085, EPI_ISL_1216087, EPI_ISL_1216088, EPI_ISL_1216090, EPI_ISL_1216092, EPI_ISL_1216093, EPI_ISL_1216095, EPI_ISL_1216097, EPI_ISL_1216099, EPI_ISL_1216100, EPI_ISL_1216104, EPI_ISL_1216106, EPI_ISL_1216107, EPI_ISL_1216109, EPI_ISL_1216110, EPI_ISL_1216112, EPI_ISL_1216114, EPI_ISL_1216116, EPI_ISL_1216118, EPI_ISL_1216119, EPI_ISL_1216121, EPI_ISL_1216123, EPI_ISL_1216124, EPI_ISL_1216127, EPI_ISL_1216128, EPI_ISL_1216130, EPI_ISL_1216132, EPI_ISL_1216133, EPI_ISL_1234527, EPI_ISL_1234532, EPI_ISL_1234534, EPI_ISL_1234535, EPI_ISL_1234536, EPI_ISL_1731545, EPI_ISL_1731548, EPI_ISL_1731551, EPI_ISL_1731554, EPI_ISL_1731560, EPI_ISL_2958637, EPI_ISL_2958648, EPI_ISL_2958649, EPI_ISL_2958650			
see above	MRCG at LSHTM Genomics lab	MRCG at LSHTM Genomics lab	Abdoulie Kanteh; Abdul Karim sesay; Bakary Sanyang; Dabiri Damilari; Jarra Manneh; Mariama Kujabi
EPI_ISL_2709478, EPI_ISL_2709479, EPI_ISL_2709480, EPI_ISL_2709481, EPI_ISL_2709482, EPI_ISL_2709483, EPI_ISL_2709484, EPI_ISL_2709485, EPI_ISL_2709486, EPI_ISL_2709487, EPI_ISL_2709488			
see above	Malawi Liverpool Wellcome Trust CLinical Research Program	Malawi Liverpool Wellcome Trust CLinical Research Program	Catherine Anscombe; Jonathan Rigby; Kayla Barnes; Nicholas Feasey; Oscar Kanjerwa; Phillip Ashton
EPI_ISL_3770646, EPI_ISL_3770647	Malawi Liverpool Wellcome Trust Clinical Research Program	Malawi Liverpool Wellcome Trust CLinical Research Program	Ben Morton; Catherine Anscombe; Kondwani Jambo; Philip Ashton; Sam Lissauer
EPI_ISL_640074	Mamre CDC wc MRC	NHLS/UCT	Arash Iranzadeh; Bruna Galvao; Carolyn Williamson; Deelan Doolabh; Diana Hardie; Innocent Mudau; Kruger Marais; Lynn Tyers; Marvin Hsiao; Stephen Korsman
EPI_ISL_2271966, EPI_ISL_2271967, EPI_ISL_2271968, EPI_ISL_2271979, EPI_ISL_2271980	Matikwana Hospital	National Institute for Communicable Diseases of the National Health Laboratory Service	Amoako DG; Bhiman JN; Ismail A; Mahlangu B; Mohale T; Ntuli N; Scheepers C
EPI_ISL_1098839, EPI_ISL_1109624, EPI_ISL_1109625, EPI_ISL_1109626, EPI_ISL_1109627, EPI_ISL_1109628, EPI_ISL_1109629, EPI_ISL_1109630, EPI_ISL_1141525, EPI_ISL_1165078, EPI_ISL_1165084, EPI_ISL_1165085, EPI_ISL_1165086, EPI_ISL_1165087, EPI_ISL_1167187, EPI_ISL_1167188, EPI_ISL_1167189, EPI_ISL_1167190, EPI_ISL_1167192, EPI_ISL_1167193, EPI_ISL_1167194, EPI_ISL_1167195, EPI_ISL_1167196, EPI_ISL_1167197			
see above	Medical Ain Shams Research Institute (MASRI), Ain Shams University	Medical Ain Shams Research Institute (MASRI), Ain Shams University	Aya Mohamed; Fatma Ebied; Hagar Elshora; Hala Hafez; Hesham Elghazaly; Hoda Ezz Elarab; Iman Foda; Manal Hamdy Elsaid; Mohamed Elhadidi; Osama Mansour.; Reham Kassab; Samia Abdou Girgis; Sara Hassan Agwa; Shimaa Moustafa
EPI_ISL_495535, EPI_ISL_495536, EPI_ISL_495537, EPI_ISL_495538, EPI_ISL_495539, EPI_ISL_495540, EPI_ISL_495541, EPI_ISL_495542, EPI_ISL_515800, EPI_ISL_515801, EPI_ISL_515802, EPI_ISL_515803, EPI_ISL_515804, EPI_ISL_515805, EPI_ISL_515806, EPI_ISL_515807, EPI_ISL_515808, EPI_ISL_515809, EPI_ISL_515810, EPI_ISL_515811, EPI_ISL_515812, EPI_ISL_515813, EPI_ISL_515814, EPI_ISL_515815, EPI_ISL_515816, EPI_ISL_515817, EPI_ISL_515818, EPI_ISL_515819, EPI_ISL_515820, EPI_ISL_515821, EPI_ISL_515822, EPI_ISL_515823, EPI_ISL_515824, EPI_ISL_515825, EPI_ISL_515826, EPI_ISL_515827, EPI_ISL_515828, EPI_ISL_515829, EPI_ISL_515830, EPI_ISL_515831, EPI_ISL_515832, EPI_ISL_515833, EPI_ISL_515834, EPI_ISL_515835, EPI_ISL_515836, EPI_ISL_515837, EPI_ISL_515838, EPI_ISL_515839, EPI_ISL_515840, EPI_ISL_515841, EPI_ISL_515842, EPI_ISL_515843, EPI_ISL_515844, EPI_ISL_515845, EPI_ISL_515846, EPI_ISL_515847, EPI_ISL_515848, EPI_ISL_515849, EPI_ISL_515850, EPI_ISL_515851, EPI_ISL_515852, EPI_ISL_515853, EPI_ISL_515854, EPI_ISL_515855, EPI_ISL_515856, EPI_ISL_515857, EPI_ISL_515858, EPI_ISL_515859, EPI_ISL_515860, EPI_ISL_515861, EPI_ISL_515862, EPI_ISL_515863, EPI_ISL_515864, EPI_ISL_515865, EPI_ISL_515866, EPI_ISL_515867, EPI_ISL_515868, EPI_ISL_515869, EPI_ISL_515870, EPI_ISL_515871, EPI_ISL_515872, EPI_ISL_515873, EPI_ISL_515874, EPI_ISL_515875, EPI_ISL_515876, EPI_ISL_515877, EPI_ISL_515878, EPI_ISL_515879, EPI_ISL_515880, EPI_ISL_515881, EPI_ISL_515882, EPI_ISL_515883, EPI_ISL_515884, EPI_ISL_515885, EPI_ISL_515886, EPI_ISL_515887, EPI_ISL_515888, EPI_ISL_515889, EPI_ISL_515890, EPI_ISL_515891, EPI_ISL_515892, EPI_ISL_515893			
see above	Medical Disagnostics Services (MDS)	KRISP, KZN Research Innovation and Sequencing Platform	Chimukangara B; ChimukangaraB; Giandhari J; Khan S; Lessells R; Mdlalose K; Pillay S; Tegally H; Wilkinson E; York D; de Oliveira T
EPI_ISL_3462061	Microbiology, Russian Research Anti-plague Institute 'Microbe'	Microbiology, Russian Research Anti-plague Institute 'Microbe'	A.A.; A.D.; A.V.; E.A.; E.V.; Fedorov; Katyshev; Kazorina; Keita, M.; Keita, S.; Krasnov; Kritsky; Kutyrev; Naryshkina; S.A.; Shcherbakova; Sosedova; V.V.; Y.M.
EPI_ISL_640025	Mitchells Plain CHC wc MHC	NHLS/UCT	Arash Iranzadeh; Bruna Galvao; Carolyn Williamson; Deelan Doolabh; Diana Hardie; Innocent Mudau; Kruger Marais; Lynn Tyers; Marvin Hsiao; Stephen Korsman
EPI_ISL_2271954	Mitchells Plain Hospital	National Institute for Communicable Diseases of the National Health Laboratory Service	Amoako DG; Bhiman JN; Ismail A; Mahlangu B; Mohale T; Ntuli N; Scheepers C
EPI_ISL_640020, EPI_ISL_640028, EPI_ISL_640065, EPI_ISL_700489, EPI_ISL_700572, EPI_ISL_700597	Mitchells Plain Hospital wc MPH	NHLS/UCT	Arash Iranzadeh; Bruna Galvao; Carolyn Williamson; Deelan Doolabh; Diana Hardie; Innocent Mudau; Kruger Marais; Lynn Tyers; Marvin Hsiao; Stephen Korsman

EPI_ISL_960148	Mitchells Plain Hospital wc MPH	National Health Laboratory Service/UCT	Arash Iranzadeh; Bruna Galvao; Carolyn Williamson; Deelan Doolabh; Diana Hardie; Innocent Mudau; Kruger Marais; Lynn Tyers; Marvin Hsiao; Stephen Korsman
EPI_ISL_467501, EPI_ISL_467502, EPI_ISL_467503, EPI_ISL_467504, EPI_ISL_467505, EPI_ISL_467506, EPI_ISL_482702, EPI_ISL_482703, EPI_ISL_482704, EPI_ISL_482705, EPI_ISL_482706, EPI_ISL_482707, EPI_ISL_482708, EPI_ISL_482709, EPI_ISL_482714, EPI_ISL_482715, EPI_ISL_482716, EPI_ISL_482717, EPI_ISL_482718, EPI_ISL_482719, EPI_ISL_482720, EPI_ISL_482721, EPI_ISL_482722, EPI_ISL_482723, EPI_ISL_482851, EPI_ISL_482852, EPI_ISL_482853, EPI_ISL_482854, EPI_ISL_482855, EPI_ISL_482856, EPI_ISL_482857, EPI_ISL_482858, EPI_ISL_482859, EPI_ISL_482860, EPI_ISL_482861, EPI_ISL_482862, EPI_ISL_482863, EPI_ISL_482864, EPI_ISL_482865, EPI_ISL_482866, EPI_ISL_482867, EPI_ISL_482868, EPI_ISL_482869, EPI_ISL_482870, EPI_ISL_482871, EPI_ISL_482872, EPI_ISL_487329, EPI_ISL_487330, EPI_ISL_487331, EPI_ISL_487332, EPI_ISL_487333, EPI_ISL_487334, EPI_ISL_487335, EPI_ISL_487336, EPI_ISL_487337, EPI_ISL_487338, EPI_ISL_487339, EPI_ISL_487340, EPI_ISL_487341			
see above	Molecular Diagnostics Services (MDS)	KRISP, KZN Research Innovation and Sequencing Platform	Chimukangara B; Giandhari J; Khan S; Lessells R; Mdlalose K; Pillay S; Tegally H; Wilkinson E; York D; de Oliveira T
EPI_ISL_614347, EPI_ISL_614351, EPI_ISL_614352, EPI_ISL_614353, EPI_ISL_614354, EPI_ISL_614355, EPI_ISL_614356, EPI_ISL_614357, EPI_ISL_614358, EPI_ISL_614359, EPI_ISL_614360, EPI_ISL_614361, EPI_ISL_614362, EPI_ISL_614363, EPI_ISL_614364, EPI_ISL_614365, EPI_ISL_614366, EPI_ISL_614367, EPI_ISL_614368, EPI_ISL_614369, EPI_ISL_614370, EPI_ISL_614371, EPI_ISL_614372, EPI_ISL_614373, EPI_ISL_614374, EPI_ISL_614375, EPI_ISL_614376, EPI_ISL_614377, EPI_ISL_614378, EPI_ISL_614379, EPI_ISL_614380, EPI_ISL_614381, EPI_ISL_614382, EPI_ISL_614383, EPI_ISL_614384, EPI_ISL_614385, EPI_ISL_614386, EPI_ISL_614387, EPI_ISL_614388, EPI_ISL_614389, EPI_ISL_614390, EPI_ISL_614391, EPI_ISL_614392, EPI_ISL_614393, EPI_ISL_614394, EPI_ISL_614395, EPI_ISL_681829, EPI_ISL_681830, EPI_ISL_681831, EPI_ISL_681832, EPI_ISL_681833, EPI_ISL_681834, EPI_ISL_681835, EPI_ISL_681836, EPI_ISL_681837, EPI_ISL_681838, EPI_ISL_681839, EPI_ISL_681840, EPI_ISL_681841, EPI_ISL_681842, EPI_ISL_682058, EPI_ISL_682059, EPI_ISL_1662578, EPI_ISL_1662579, EPI_ISL_1662580, EPI_ISL_1662581, EPI_ISL_1662582, EPI_ISL_1662583, EPI_ISL_1662584, EPI_ISL_1662585, EPI_ISL_1662586, EPI_ISL_1662587, EPI_ISL_1662588, EPI_ISL_1662589, EPI_ISL_1662590, EPI_ISL_1662591, EPI_ISL_1662592			
see above	Molecular diagnostic unit for viral haemorrhagic fevers and emerging viruses, Bouaké CHU Laboratory	Project group Epidemology of Highly Pathogenic Microorganisms, Robert Koch-Institute	Adjaratou Traoré; Bamba Fatoumata Touré; Chantal Akoua-Koffi; Coulibaly Mbegnan; Diané Bamourou; Essia Belarbi; Etilé Anoh; Fabian Leendertz; Grit Schubert; Kra Ouffoué; Monemo Pacome; Safiatou Karidioula; Soundélé Maïté
EPI_ISL_700479	Mowbray Maternity Hospital wc MMH	NHLS/UCT	Arash Iranzadeh; Bruna Galvao; Carolyn Williamson; Deelan Doolabh; Diana Hardie; Innocent Mudau; Kruger Marais; Lynn Tyers; Marvin Hsiao; Stephen Korsman
EPI_ISL_682330, EPI_ISL_682332, EPI_ISL_682334, EPI_ISL_682335, EPI_ISL_682337, EPI_ISL_682338, EPI_ISL_682342, EPI_ISL_682344, EPI_ISL_912537, EPI_ISL_949630			
see above	NHLS Universitas Academic	UFS Virology	D Goedhals; Emmanuel Ogunbayo; MM Nyaga; MT Mogotsi; P Nthiga; PA Bester; T de Oliveira
EPI_ISL_467509, EPI_ISL_467510, EPI_ISL_467511, EPI_ISL_467512, EPI_ISL_467513, EPI_ISL_467514, EPI_ISL_467517, EPI_ISL_467521, EPI_ISL_467522, EPI_ISL_467523, EPI_ISL_467524, EPI_ISL_482710, EPI_ISL_482711, EPI_ISL_482712, EPI_ISL_482713, EPI_ISL_482724, EPI_ISL_482725, EPI_ISL_482726, EPI_ISL_482727, EPI_ISL_482728, EPI_ISL_482729, EPI_ISL_482730, EPI_ISL_482731, EPI_ISL_482848, EPI_ISL_482849, EPI_ISL_482850, EPI_ISL_487277, EPI_ISL_487278, EPI_ISL_487279, EPI_ISL_487280, EPI_ISL_487281, EPI_ISL_487282, EPI_ISL_487283, EPI_ISL_487284, EPI_ISL_487285, EPI_ISL_487286, EPI_ISL_487287, EPI_ISL_487288, EPI_ISL_487289, EPI_ISL_487290, EPI_ISL_487291, EPI_ISL_487292, EPI_ISL_487293, EPI_ISL_487294, EPI_ISL_487295, EPI_ISL_487296, EPI_ISL_487297, EPI_ISL_487298, EPI_ISL_487299, EPI_ISL_487300, EPI_ISL_487301, EPI_ISL_487302, EPI_ISL_487303, EPI_ISL_487304, EPI_ISL_487305, EPI_ISL_487306, EPI_ISL_487307, EPI_ISL_487308, EPI_ISL_487309, EPI_ISL_487310, EPI_ISL_487311, EPI_ISL_487312, EPI_ISL_487313, EPI_ISL_487314, EPI_ISL_487315, EPI_ISL_487316, EPI_ISL_487317, EPI_ISL_487318, EPI_ISL_487319, EPI_ISL_487320, EPI_ISL_487321, EPI_ISL_487322, EPI_ISL_487323, EPI_ISL_487324, EPI_ISL_487325, EPI_ISL_487326, EPI_ISL_487327, EPI_ISL_487328, EPI_ISL_487348, EPI_ISL_495516, EPI_ISL_495517, EPI_ISL_495518, EPI_ISL_495519, EPI_ISL_495520, EPI_ISL_495521, EPI_ISL_495522, EPI_ISL_495523, EPI_ISL_495524, EPI_ISL_495525, EPI_ISL_495526, EPI_ISL_495527, EPI_ISL_495528, EPI_ISL_495529, EPI_ISL_495530, EPI_ISL_495531, EPI_ISL_495532, EPI_ISL_495533, EPI_ISL_495534, EPI_ISL_495543, EPI_ISL_495544, EPI_ISL_495545, EPI_ISL_495546, EPI_ISL_495547, EPI_ISL_495548, EPI_ISL_495549, EPI_ISL_495550, EPI_ISL_495551, EPI_ISL_495552, EPI_ISL_495553, EPI_ISL_495554, EPI_ISL_495555, EPI_ISL_495556, EPI_ISL_495557, EPI_ISL_495558, EPI_ISL_495559, EPI_ISL_495560, EPI_ISL_495561, EPI_ISL_495562, EPI_ISL_498054, EPI_ISL_498055, EPI_ISL_498056, EPI_ISL_498057, EPI_ISL_498058, EPI_ISL_498059, EPI_ISL_498060, EPI_ISL_498061, EPI_ISL_498062, EPI_ISL_498063, EPI_ISL_498064, EPI_ISL_498065, EPI_ISL_498066, EPI_ISL_498067, EPI_ISL_498068, EPI_ISL_498069, EPI_ISL_498070, EPI_ISL_498071, EPI_ISL_498072, EPI_ISL_498073, EPI_ISL_498074, EPI_ISL_498075, EPI_ISL_498076, EPI_ISL_498077, EPI_ISL_498078, EPI_ISL_498079, EPI_ISL_498080, EPI_ISL_498081, EPI_ISL_498082, EPI_ISL_498083, EPI_ISL_498084, EPI_ISL_498085, EPI_ISL_498086, EPI_ISL_498087, EPI_ISL_498088, EPI_ISL_498089, EPI_ISL_498090, EPI_ISL_498091, EPI_ISL_498092, EPI_ISL_498093, EPI_ISL_498094, EPI_ISL_498095, EPI_ISL_498096, EPI_ISL_498097, EPI_ISL_498098, EPI_ISL_498099, EPI_ISL_498100, EPI_ISL_498101, EPI_ISL_498102, EPI_ISL_498103, EPI_ISL_498104, EPI_ISL_498105, EPI_ISL_498106, EPI_ISL_498107, EPI_ISL_498108, EPI_ISL_498109, EPI_ISL_498110, EPI_ISL_498111, EPI_ISL_498112, EPI_ISL_498113, EPI_ISL_498114, EPI_ISL_498115, EPI_ISL_498116, EPI_ISL_498117, EPI_ISL_498118, EPI_ISL_498119, EPI_ISL_498120, EPI_ISL_498121, EPI_ISL_498122, EPI_ISL_498123, EPI_ISL_498124, EPI_ISL_498125, EPI_ISL_498126, EPI_ISL_509223, EPI_ISL_509224, EPI_ISL_509225, EPI_ISL_509226, EPI_ISL_509227, EPI_ISL_509228, EPI_ISL_509229, EPI_ISL_509230, EPI_ISL_509231, EPI_ISL_509232, EPI_ISL_509233, EPI_ISL_509234, EPI_ISL_509235, EPI_ISL_509236, EPI_ISL_509237, EPI_ISL_509238, EPI_ISL_509239, EPI_ISL_509240, EPI_ISL_509241, EPI_ISL_509242, EPI_ISL_509243, EPI_ISL_509244, EPI_ISL_509245, EPI_ISL_509246, EPI_ISL_509247, EPI_ISL_509248, EPI_ISL_509249, EPI_ISL_509250, EPI_ISL_509251, EPI_ISL_509252, EPI_ISL_509253, EPI_ISL_509254, EPI_ISL_509255, EPI_ISL_509256, EPI_ISL_509257, EPI_ISL_509258, EPI_ISL_509259, EPI_ISL_509260, EPI_ISL_509261, EPI_ISL_509262, EPI_ISL_509263, EPI_ISL_509264, EPI_ISL_509265, EPI_ISL_509266, EPI_ISL_509267, EPI_ISL_509268, EPI_ISL_509269, EPI_ISL_509270, EPI_ISL_509271, EPI_ISL_509272, EPI_ISL_509273, EPI_ISL_509274, EPI_ISL_509275, EPI_ISL_509276, EPI_ISL_509277, EPI_ISL_509278, EPI_ISL_509279, EPI_ISL_509280, EPI_ISL_509281, EPI_ISL_509282, EPI_ISL_509283, EPI_ISL_509284, EPI_ISL_509285, EPI_ISL_509286, EPI_ISL_509287, EPI_ISL_509288, EPI_ISL_509289, EPI_ISL_509290, EPI_ISL_509291, EPI_ISL_509292, EPI_ISL_509293, EPI_ISL_509294, EPI_ISL_509295, EPI_ISL_509296, EPI_ISL_509297, EPI_ISL_509298, EPI_ISL_509299, EPI_ISL_509300, EPI_ISL_509301, EPI_ISL_509302, EPI_ISL_509303, EPI_ISL_509304, EPI_ISL_509305, EPI_ISL_509306, EPI_ISL_509307, EPI_ISL_509308, EPI_ISL_509309, EPI_ISL_509310, EPI_ISL_509311, EPI_ISL_509312, EPI_ISL_509313, EPI_ISL_509314, EPI_ISL_509315, EPI_ISL_509316, EPI_ISL_509317, EPI_ISL_509318, EPI_ISL_509319, EPI_ISL_509320, EPI_ISL_509321, EPI_ISL_509322, EPI_ISL_509323, EPI_ISL_509324, EPI_ISL_509325, EPI_ISL_509326, EPI_ISL_509327, EPI_ISL_509328, EPI_ISL_509329, EPI_ISL_509330, EPI_ISL_509331, EPI_ISL_509332, EPI_ISL_509333, EPI_ISL_509334, EPI_ISL_509335, EPI_ISL_509336, EPI_ISL_509337, EPI_ISL_509338, EPI_ISL_509339, EPI_ISL_509340, EPI_ISL_509341, EPI_ISL_509342, EPI_ISL_509343, EPI_ISL_509344, EPI_ISL_509345, EPI_ISL_509346, EPI_ISL_509347, EPI_ISL_509348, EPI_ISL_509349, EPI_ISL_509350, EPI_ISL_509351, EPI_ISL_509352, EPI_ISL_509353, EPI_ISL_509354, EPI_ISL_509355, EPI_ISL_509356, EPI_ISL_509357, EPI_ISL_509358, EPI_ISL_509359, EPI_ISL_509360, EPI_ISL_509361, EPI_ISL_509362, EPI_ISL_509363, EPI_ISL_509364, EPI_ISL_509365, EPI_ISL_509366, EPI_ISL_509367, EPI_ISL_509368, EPI_ISL_509369, EPI_ISL_509370, EPI_ISL_509371, EPI_ISL_515568, EPI_ISL_515569, EPI_ISL_515570, EPI_ISL_515571, EPI_ISL_515572, EPI_ISL_515573, EPI_ISL_515574, EPI_ISL_515575, EPI_ISL_515576, EPI_ISL_515577, EPI_ISL_515578, EPI_ISL_515579, EPI_ISL_515580, EPI_ISL_515581, EPI_ISL_515582, EPI_ISL_515583, EPI_ISL_515584, EPI_ISL_515585, EPI_ISL_515586, EPI_ISL_515587, EPI_ISL_515588, EPI_ISL_515589, EPI_ISL_515590, EPI_ISL_515591, EPI_ISL_515592, EPI_ISL_515593, EPI_ISL_515594, EPI_ISL_515595, EPI_ISL_515596, EPI_ISL_515597, EPI_ISL_515598, EPI_ISL_515599, EPI_ISL_515600, EPI_ISL_515601, EPI_ISL_515602, EPI_ISL_515603, EPI_ISL_515604, EPI_ISL_515605, EPI_ISL_515606, EPI_ISL_515607, EPI_ISL_515608, EPI_ISL_515609, EPI_ISL_515610, EPI_ISL_515611, EPI_ISL_515612, EPI_ISL_515613, EPI_ISL_515614, EPI_ISL_515615, EPI_ISL_515616, EPI_ISL_515617, EPI_ISL_515618, EPI_ISL_515619, EPI_ISL_515620, EPI_ISL_515621, EPI_ISL_515622, EPI_ISL_515623, EPI_ISL_515624, EPI_ISL_515625, EPI_ISL_515626, EPI_ISL_515627, EPI_ISL_515628, EPI_ISL_515629, EPI_ISL_515630, EPI_ISL_515631, EPI_ISL_515632, EPI_ISL_515633, EPI_ISL_515634, EPI_ISL_515635, EPI_ISL_515636, EPI_ISL_515637, EPI_ISL_515638, EPI_ISL_515639, EPI_ISL_515640, EPI_ISL_515641, EPI_ISL_515642, EPI_ISL_515643, EPI_ISL_515644, EPI_ISL_515645, EPI_ISL_515646, EPI_ISL_515647, EPI_ISL_515648, EPI_ISL_515649, EPI_ISL_515650, EPI_ISL_515651, EPI_ISL_515652, EPI_ISL_515653, EPI_ISL_515654, EPI_ISL_515655, EPI_ISL_515656, EPI_ISL_515657, EPI_ISL_515658, EPI_ISL_515659, EPI_ISL_515660, EPI_ISL_515661, EPI_ISL_515662, EPI_ISL_515663, EPI_ISL_515664, EPI_ISL_515665, EPI_ISL_515666, EPI_ISL_515667, EPI_ISL_515668, EPI_ISL_515669, EPI_ISL_515670, EPI_ISL_515671, EPI_ISL_515672, EPI_ISL_515673, EPI_ISL_515674, EPI_ISL_515675, EPI_ISL_515676, EPI_ISL_515677, EPI_ISL_515678, EPI_ISL_515679, EPI_ISL_515680, EPI_ISL_515681, EPI_ISL_515682, EPI_ISL_515683, EPI_ISL_515684, EPI_ISL_515685, EPI_ISL_515686, EPI_ISL_515687, EPI_ISL_515688, EPI_ISL_515689, EPI_ISL_515690, EPI_ISL_515691, EPI_ISL_515692, EPI_ISL_515693, EPI_ISL_515694, EPI_ISL_515695, EPI_ISL_515696, EPI_ISL_515697, EPI_ISL_515698, EPI_ISL_515699, EPI_ISL_515700, EPI_ISL_515701, EPI_ISL_515702, EPI_ISL_515703, EPI_ISL_515704, EPI_ISL_515705, EPI_ISL_515706, EPI_ISL_515707, EPI_ISL_515708, EPI_ISL_515709, EPI_ISL_515710, EPI_ISL_515711, EPI_ISL_515712, EPI_ISL_515713, EPI_ISL_515714, EPI_ISL_515715, EPI_ISL_515716, EPI_ISL_515717, EPI_ISL_515718, EPI_ISL_515719, EPI_ISL_515720, EPI_ISL_515721, EPI_ISL_515722, EPI_ISL_515723, EPI_ISL_515724, EPI_ISL_515725, EPI_ISL_515726, EPI_ISL_515727, EPI_ISL_515728, EPI_ISL_515729, EPI_ISL_515730, EPI_ISL_515731, EPI_ISL_515732, EPI_ISL_515733, EPI_ISL_515734, EPI_ISL_515735, EPI_ISL_515736, EPI_ISL_515737, EPI_ISL_515738, EPI_ISL_515739, EPI_ISL_515740, EPI_ISL_515741, EPI_ISL_515742, EPI_ISL_515743, EPI_ISL_515744, EPI_ISL_515745, EPI_ISL_515746, EPI_ISL_515747, EPI_ISL_515748, EPI_ISL_515749, EPI_ISL_515750, EPI_ISL_515751, EPI_ISL_515752, EPI_ISL_515753, EPI_ISL_515754, EPI_ISL_515755, EPI_ISL_515756, EPI_ISL_515757, EPI_ISL_515758, EPI_ISL_515759, EPI_ISL_515760, EPI_ISL_515761, EPI_ISL_515762, EPI_ISL_515763, EPI_ISL_515764, EPI_ISL_515765, EPI_ISL_515766, EPI_ISL_515767, EPI_ISL_515768, EPI_ISL_515769, EPI_ISL_515770, EPI_ISL_515771, EPI_ISL_515772, EPI_ISL_515773, EPI_ISL_515774, EPI_ISL_515775, EPI_ISL_515776, EPI_ISL_515777, EPI_ISL_515778, EPI_ISL_515779, EPI_ISL_515780, EPI_ISL_515781, EPI_ISL_515782, EPI_ISL_515783, EPI_ISL_515784, EPI_ISL_515785, EPI_ISL_515786, EPI_ISL_515787, EPI_ISL_515788, EPI_ISL_515789, EPI_ISL_515790, EPI_ISL_515791, EPI_ISL_515792, EPI_ISL_515793, EPI_ISL_515794, EPI_ISL_515795, EPI_ISL_515796, EPI_ISL_515797, EPI_ISL_515798, EPI_ISL_515799, EPI_ISL_518033, EPI_ISL_518034, EPI_ISL_518035, EPI_ISL_518036, EPI_ISL_518037, EPI_ISL_518038, EPI_ISL_518039, EPI_ISL_518040, EPI_ISL_518041, EPI_ISL_518042, EPI_ISL_518043, EPI_ISL_518044, EPI_ISL_518045, EPI_ISL_518046, EPI_ISL_518047, EPI_ISL_518048, EPI_ISL_518049, EPI_ISL_518050, EPI_ISL_518051, EPI_ISL_518052, EPI_ISL_518053, EPI_ISL_529720, EPI_ISL_529721, EPI_ISL_529722, EPI_ISL_529723, EPI_ISL_529724, EPI_ISL_529725, EPI_ISL_529726, EPI_ISL_529727, EPI_ISL_529728, EPI_ISL_529729, EPI_ISL_529730, EPI_ISL_529731, EPI_ISL_529732, EPI_ISL_529733, EPI_ISL_529734, EPI_ISL_529735, EPI_ISL_529736, EPI_ISL_529737, EPI_ISL_529738, EPI_ISL_529739, EPI_ISL_529740, EPI_ISL_529741, EPI_ISL_529742, EPI_ISL_529743, EPI_ISL_529744, EPI_ISL_529745, EPI_ISL_529746, EPI_ISL_529747, EPI_ISL_529748, EPI_ISL_529749, EPI_ISL_529750, EPI_ISL_529751, EPI_ISL_529752, EPI_ISL_529753, EPI_ISL_529754, EPI_ISL_529755, EPI_ISL_529756, EPI_ISL_529757, EPI_ISL_529758, EPI_ISL_529759, EPI_ISL_529760, EPI_ISL_529761, EPI_ISL_529762, EPI_ISL_529763, EPI_ISL_529764, EPI_ISL_529765, EPI_ISL_529766, EPI_ISL_529767, EPI_ISL_529768, EPI_ISL_529769, EPI_ISL_529770, EPI_ISL_529771, EPI_ISL_529772, EPI_ISL_529773, EPI_ISL_529774, EPI_ISL_529775, EPI_ISL_529776, EPI_ISL_529777, EPI_ISL_529778, EPI_ISL_529779, EPI_ISL_529780, EPI_ISL_529781, EPI_ISL_529782, EPI_ISL_529783, EPI_ISL_529784, EPI_ISL_529785, EPI_ISL_529786, EPI_ISL_529787, EPI_ISL_529788, EPI_ISL_529789, EPI_ISL_529790, EPI_ISL_529791, EPI_ISL_529792, EPI_ISL_529793, EPI_ISL_529794, EPI_ISL_529795, EPI_ISL_529796, EPI_ISL_529797, EPI_ISL_529798, EPI_ISL_529799, EPI_ISL_529800, EPI_ISL_529801, EPI_ISL_529802, EPI_ISL_529803, EPI_ISL_535390, EPI_ISL_535391, EPI_ISL_535392, EPI_ISL_535393, EPI_ISL_535394, EPI_ISL_535395, EPI_ISL_535396, EPI_ISL_535397, EPI_ISL_535398, EPI_ISL_535399, EPI_ISL_535400, EPI_ISL_535401, EPI_ISL_535402, EPI_ISL_535403, EPI_ISL_535404, EPI_ISL_535405, EPI_ISL_535406, EPI_ISL_535407, EPI_ISL_535408, EPI_ISL_535409, EPI_ISL_535410, EPI_ISL_535411, EPI_ISL_535412, EPI_ISL_535413, EPI_ISL_535414, EPI_ISL_535415, EPI_ISL_535416, EPI_ISL_535417, EPI_ISL_535418, EPI_ISL_535419, EPI_ISL_535420, EPI_ISL_535421, EPI_ISL_535422, EPI_ISL_535423, EPI_ISL_535424, EPI_ISL_535425, EPI_ISL_535426, EPI_ISL_535427, EPI_ISL_535428, EPI_ISL_535429, EPI_ISL_535430, EPI_ISL_535431, EPI_ISL_535432, EPI_ISL_535433, EPI_ISL_535434, EPI_ISL_535435, EPI_ISL_535436, EPI_ISL_535437, EPI_ISL_535438, EPI_ISL_535439, EPI_ISL_535440, EPI_ISL_535441, EPI_ISL_535442, EPI_ISL_535443, EPI_ISL_535444, EPI_ISL_535445, EPI_ISL_535446, EPI_ISL_535447, EPI_ISL_535448, EPI_ISL_535449, EPI_ISL_535450, EPI_ISL_535451, EPI_ISL_535452, EPI_ISL_535453, EPI_ISL_535454, EPI_ISL_535455, EPI_ISL_535456, EPI_ISL_535457, EPI_ISL_535458, EPI_ISL_535459, EPI_ISL_535460, EPI_ISL_535461, EPI_ISL_535462, EPI_ISL_535463, EPI_ISL_535464, EPI_ISL_535465, EPI_ISL_535466, EPI_ISL_535467, EPI_ISL_535468, EPI_ISL_535469, EPI_ISL_535470, EPI_ISL_535471, EPI_ISL_535472, EPI_ISL_535473, EPI_ISL_535474, EPI_ISL_535475, EPI_ISL_535476, EPI_ISL_535477, EPI_ISL_535478, EPI_ISL_535479, EPI_ISL_535480, EPI_ISL_535481, EPI_ISL_535482, EPI_ISL_535483, EPI_ISL_535484, EPI_ISL_535485, EPI_ISL_535486, EPI_ISL_535487, EPI_ISL_535488, EPI_ISL_535496, EPI_ISL_535497, EPI_ISL_535498, EPI_ISL_535499, EPI_ISL_535500, EPI_ISL_535501, EPI_ISL_535502, EPI_ISL_535503, EPI_ISL_535504, EPI_ISL_535505, EPI_ISL_535506, EPI_ISL_535507, EPI_ISL_535508, EPI_ISL_535509, EPI_ISL_535510, EPI_ISL_535511, EPI_ISL_535512, EPI_ISL_535513, EPI_ISL_535514, EPI_ISL_535515, EPI_ISL_535516, EPI_ISL_535517, EPI_ISL_535518, EPI_ISL_535519, EPI_ISL_535520, EPI_ISL_535521, EPI_ISL_535522, EPI_ISL_535523, EPI_ISL_535524, EPI_ISL_535525, EPI_ISL_535526, EPI_ISL_535527, EPI_ISL_535528, EPI_ISL_535529, EPI_ISL_535530, EPI_ISL_535531, EPI_ISL_535532, EPI_ISL_535533, EPI_ISL_535534, EPI_ISL_535535, EPI_ISL_535536, EPI_ISL_535537, EPI_ISL_535538, EPI_ISL_535539, EPI_ISL_535540, EPI_ISL_535541, EPI_ISL_535542, EPI_ISL_535543, EPI_ISL_535544, EPI_ISL_535545, EPI_ISL_535546, EPI_ISL_535547, EPI_ISL_535548, EPI_ISL_535549, EPI_ISL_535550, EPI_ISL_535551, EPI_ISL_535552, EPI_ISL_535553, EPI_ISL_535554, EPI_ISL_535555, EPI_ISL_535556, EPI_ISL_535557, EPI_ISL_535558, EPI_ISL_535559, EPI_ISL_535560, EPI_ISL_535561, EPI_ISL_535562, EPI_ISL_535563, EPI_ISL_535564, EPI_ISL_535565, EPI_ISL_535566, EPI_ISL_535567, EPI_ISL_535568, EPI_ISL_535569, EPI_ISL_535570, EPI_ISL_535571, EPI_ISL_535572, EPI_ISL_602632, EPI_ISL_602633, EPI_ISL_602634, EPI_ISL_602635, EPI_ISL_602636, EPI_ISL_602637, EPI_ISL_602638, EPI_ISL_602639, EPI_ISL_602640, EPI_ISL_602641, EPI_ISL_602642, EPI_ISL_602643, EPI_ISL_602644, EPI_ISL_602645, EPI_ISL_602646, EPI_ISL_602647, EPI_ISL_602648, EPI_ISL_602649, EPI_ISL_602650, EPI_ISL_602651, EPI_ISL_602652, EPI_ISL_602653, EPI_ISL_602654, EPI_ISL_602655, EPI_ISL_602656, EPI_ISL_602657, EPI_ISL_602658, EPI_ISL_602659, EPI_ISL_602660, EPI_ISL_602661, EPI_ISL_602662, EPI_ISL_602663, EPI_ISL_602664, EPI_ISL_602665, EPI_ISL_602666, EPI_ISL_602667, EPI_ISL_602668, EPI_ISL_602669, EPI_ISL_602670, EPI_ISL_602671, EPI_ISL_602672, EPI_ISL_602673, EPI_ISL_602674, EPI_ISL_602675, EPI_ISL_602676, EPI_ISL_602677, EPI_ISL_602678, EPI_ISL_602679, EPI_ISL_602680, EPI_ISL_602681, EPI_ISL_602682, EPI_ISL_602683, EPI_ISL_602684, EPI_ISL_602685, EPI_ISL_602686, EPI_ISL_602687, EPI_ISL_602688, EPI_ISL_602689, EPI_ISL_602690, EPI_ISL_602691, EPI_ISL_602692, EPI_ISL_602693, EPI_ISL_602694, EPI_ISL_602695, EPI_ISL_602696, EPI_ISL_602697, EPI_ISL_602698, EPI_ISL_602699, EPI_ISL_602700, EPI_ISL_602701, EPI_ISL_602702, EPI_ISL_602703, EPI_ISL_602704, EPI_ISL_602705, EPI_ISL_602706, EPI_ISL_602707, EPI_ISL_602708, EPI_ISL_602709, EPI_ISL_602710, EPI_ISL_602711, EPI_ISL_602712, EPI_ISL_602713, EPI_ISL_602714, EPI_ISL_602715, EPI_ISL_602716, EPI_ISL_602717, EPI_ISL_602718, EPI_ISL_602719, EPI_ISL_602720, EPI_ISL_602721, EPI_ISL_602722, EPI_ISL_602723, EPI_ISL_602724, EPI_ISL_602725, EPI_ISL_602726, EPI_ISL_602727, EPI_ISL_602728, EPI_ISL_602729, EPI_ISL_602730, EPI_ISL_602731, EPI_ISL_602732, EPI_ISL_602733, EPI_ISL_602734, EPI_ISL_602735, EPI_ISL_602736, EPI_ISL_602737, EPI_ISL_602738, EPI_ISL_602739, EPI_ISL_602740, EPI_ISL_602741, EPI_ISL_602742, EPI_ISL_602743, EPI_ISL_602744, EPI_ISL_602745, EPI_ISL_602746, EPI_ISL_602747, EPI_ISL_602748, EPI_ISL_602749, EPI_ISL_602750, EPI_ISL_602751, EPI_ISL_602752, EPI_ISL_602753, EPI_ISL_602754, EPI_ISL_602755, EPI_ISL_602756, EPI_ISL_602757, EPI_ISL_602758, EPI_ISL_602759, EPI_ISL_602760, EPI_ISL_602761, EPI_ISL_602762, EPI_ISL_602763, EPI_ISL_602764, EPI_ISL_602765, EPI_ISL_602766, EPI_ISL_602767, EPI_ISL_602768, EPI_ISL_602769, EPI_ISL_602770, EPI_ISL_602771, EPI_ISL_602772, EPI_ISL_602773, EPI_ISL_602774, EPI_ISL_602775, EPI_ISL_602776, EPI_ISL_602777, EPI_ISL_602778, EPI_ISL_602779, EPI_ISL_602780, EPI_ISL_602781, EPI_ISL_602782, EPI_ISL_602783, EPI_ISL_602784, EPI_ISL_602785, EPI_ISL_602786, EPI_ISL_602787, EPI_ISL_602788, EPI_ISL_602789, EPI_ISL_602790, EPI_ISL_602791, EPI_ISL_602792, EPI_ISL_602793, EPI_ISL_602794, EPI_ISL_602795, EPI_ISL_602796, EPI_ISL_602797, EPI_ISL_602798, EPI_ISL_602799, EPI_ISL_602800, EPI_ISL_602801, EPI_ISL_602802, EPI_ISL_602803, EPI_ISL_602804, EPI_ISL_602805, EPI_ISL_602806, EPI_ISL_602807, EPI_ISL_602808, EPI_ISL_602809, EPI_ISL_602810, EPI_ISL_602811, EPI_ISL_602812, EPI_ISL_602813, EPI_ISL_602814, EPI_ISL_602815, EPI_ISL_602816, EPI_ISL_602817, EPI_ISL_602818, EPI_ISL_602819, EPI_ISL_602820, EPI_ISL_602821, EPI_ISL_602822, EPI_ISL_602823, EPI_ISL_602824, EPI_ISL_602825, EPI_ISL_602826, EPI_ISL_602827, EPI_ISL_602828, EPI_ISL_602829, EPI_ISL_602830, EPI_ISL_602831, EPI_ISL_602832, EPI_ISL_602833, EPI_ISL_602834, EPI_ISL_602835, EPI_ISL_602836, EPI_ISL_602837, EPI_ISL_602838, EPI_ISL_602839, EPI_ISL_602840, EPI_ISL_602841, EPI_ISL_602842, EPI_ISL_602843, EPI_ISL_602844, EPI_ISL_602845, EPI_ISL_602846, EPI_ISL_602847, EPI_ISL_602848, EPI_ISL_602849, EPI_ISL_602850, EPI_ISL_602851, EPI_ISL_602852, EPI_ISL_602853, EPI_ISL_602854, EPI_ISL_602855, EPI_ISL_602856, EPI_ISL_602857, EPI_ISL_602858, EPI_ISL_602859, EPI_ISL_602860, EPI_ISL_602861, EPI_ISL_602862, EPI_ISL_602863, EPI_ISL_602864, EPI_ISL_602865, EPI_ISL_602866, EPI_ISL_602912, EPI_ISL_602913, EPI_ISL_602914, EPI_ISL_602915, EPI_ISL_602916, EPI_ISL_602917, EPI_ISL_602918, EPI_ISL_602919, EPI_ISL_602920, EPI_ISL_602921, EPI_ISL_602922, EPI_ISL_602923, EPI_ISL_602924, EPI_ISL_602925, EPI_ISL_602926, EPI_ISL_602927, EPI_ISL_602928, EPI_ISL_602929, EPI_ISL_602930, EPI_ISL_605785, EPI_ISL_605786, EPI_ISL_605789, EPI_ISL_660165, EPI_ISL_660166, EPI_ISL_660167, EPI_ISL_660168, EPI_ISL_660169, EPI_ISL_660170, EPI_ISL_660171, EPI_ISL_660172, EPI_ISL_660173, EPI_ISL_660174, EPI_ISL_660175, EPI_ISL_660176, EPI_ISL_660177, EPI_ISL_660178, EPI_ISL_660179, EPI_ISL_660180, EPI_ISL_660181, EPI_ISL_660182, EPI_ISL_660183, EPI_ISL_2397307			
see above	NHLS-IALCH	KRISP, KZN Research Innovation and Sequencing Platform	Bernstein M; COMMIT-KZN Team; Cele S; Chimukangara B; Gazy I; Giandhari J; Gosnell BI; Hanekom W; Karim F; Khan K; Khan S; Lessells R; Lessells RJ; Manickchund N; Mdlalose K; Mlisana K; Moosa MYS; Msomi N; Pillay S; Ramphal U; San JE; Sigal A; Singh L; Tegally H; Wilkinson E; York D; de Oliveira T
EPI_ISL_766861, EPI_ISL_766862, EPI_ISL_766863, EPI_ISL_766864, EPI_ISL_766865, EPI_ISL_766866, EPI_ISL_766867, EPI_ISL_766869, EPI_ISL_766870, EPI_ISL_766871, EPI_ISL_766872, EPI_ISL_766873, EPI_ISL_766874, EPI_ISL_766875			
see above	NIC Viral Respiratory Unit - Institut Pasteur of Algeria	National Reference Center for Viruses of Respiratory Infections, Institut Pasteur, Paris	Angela Brisebarre; Etienne Simon-Lorière; Fawzi Derrar; Flora Donati; Marion Barbet; Maud Vanpeene; Mélanie Albert; Méline Bizard; Sylvie Behillil; Sylvie van der Werf; Vincent Enouf
EPI_ISL_1208976, EPI_ISL_1208986, EPI_ISL_1208987, EPI_ISL_1208988, EPI_ISL_1208989, EPI_ISL_1208994, EPI_ISL_1208996			
see above	Namibia Institute of Pathology (NIP)	National Institute for Communicable Diseases of the National Health Laboratory Service	Amoako DG; Bhiman JN; Ismail A; Konstantinus I; Mahlangu B; Mohale T; Ntuli N; Scheepers C; Van Rooyen G
EPI_ISL_622942, EPI_ISL_622944, EPI_ISL_622945, EPI_ISL_622958, EPI_ISL_622970, EPI_ISL_622971, EPI_ISL_622972, EPI_ISL_622973, EPI_ISL_622974, EPI_ISL_622976, EPI_ISL_622977, EPI_ISL_622979, EPI_ISL_622980, EPI_ISL_622981, EPI_ISL_622982, EPI_ISL_622984, EPI_ISL_622987, EPI_ISL_622991, EPI_ISL_622992, EPI_ISL_622995, EPI_ISL_622996, EPI_ISL_622997, EPI_ISL_622998, EPI_ISL_622999, EPI_ISL_623000, EPI_ISL_623001, EPI_ISL_623002, EPI_ISL_623003, EPI_ISL_623004, EPI_ISL_623005, EPI_ISL_623006, EPI_ISL_623007, EPI_ISL_623008, EPI_ISL_623009, EPI_ISL_623010, EPI_ISL_623011, EPI_ISL_623012, EPI_ISL_623013, EPI_ISL_623014, EPI_ISL_623015, EPI_ISL_623016, EPI_ISL_623017, EPI_ISL_623018, EPI_ISL_623019, EPI_ISL_623020, EPI_ISL_623021, EPI_ISL_623022, EPI_ISL_623023, EPI_ISL_623024, EPI_ISL_623026, EPI_ISL_623027, EPI_ISL_623029, EPI_ISL_623030, EPI_ISL_623031, EPI_ISL_623032, EPI_ISL_623033, EPI_ISL_623034, EPI_ISL_623035, EPI_ISL_623036, EPI_ISL_623037, EPI_ISL_623038, EPI_ISL_623039, EPI_ISL_623040, EPI_ISL_623041, EPI_ISL_623042, EPI_ISL_623043, EPI_ISL_623044, EPI_ISL_623045, EPI_ISL_623046, EPI_ISL_623047, EPI_ISL_623048, EPI_ISL_623049, EPI_ISL_623050, EPI_ISL_623051, EPI_ISL_623052, EPI_ISL_623053, EPI_ISL_623054, EPI_ISL_623055, EPI_ISL_623056, EPI_ISL_623057, EPI_ISL_623058, EPI_ISL_623059, EPI_ISL_623060, EPI_ISL_623061, EPI_ISL_623062, EPI_ISL_623065, EPI_ISL_623066, EPI_ISL_623067, EPI_ISL_623069, EPI_ISL_623070, EPI_ISL_623071, EPI_ISL_623072			
see above	National Health Laboratory Service	National Institute for Communicable Diseases of the National Health Laboratory Service	Allam M; Bhiman JN; Ismail A; Khumalo Z; Kwenda S; Mnyameni F; Mohale T; Mtshali P; Subramoney K
EPI_ISL_634978, EPI_ISL_634979, EPI_ISL_634980, EPI_ISL_634981, EPI_ISL_634982, EPI_ISL_634983, EPI_ISL_634984, EPI_ISL_634985, EPI_ISL_634986, EPI_ISL_634987, EPI_ISL_634988, EPI_ISL_634989, EPI_ISL_634990, EPI_ISL_634991, EPI_ISL_634992			
see above	National Health Laboratory Service - Inkosi Albert Luthuli Central Hospital (NHLS-IALCH)	KRISP, KZN Research Innovation and Sequencing Platform	Giandhari J; Khan S; Lessells R; Mdlalose K; Pillay S; Tegally H; Wilkinson E; York D; de Oliveira T
EPI_ISL_1363146, EPI_ISL_1363147, EPI_ISL_1363148, EPI_ISL_1363149, EPI_ISL_1363150, EPI_ISL_1372563, EPI_ISL_1373592, EPI_ISL_1373596			
see above	National Health Laboratory Services	National Health Laboratory Services	Arshad Ismail; Florette Treurnicht; Kathleen Subramoney; Mushal Ali; Thabo Mohale
EPI_ISL_4078219, EPI_ISL_4078230, EPI_ISL_4078244, EPI_ISL_4078245	National Health Laboratory Services, Virology	National Health Laboratory Services, Virology	Florette Treurnicht; Kathleen Subramoney
EPI_ISL_490271, EPI_ISL_490272, EPI_ISL_490273, EPI_ISL_490274, EPI_ISL_490275, EPI_ISL_490276, EPI_ISL_490277, EPI_ISL_490278, EPI_ISL_490279, EPI_ISL_490280, EPI_ISL_490281, EPI_ISL_490282, EPI_ISL_490283, EPI_ISL_490284, EPI_ISL_490285, EPI_ISL_490286, EPI_ISL_490287, EPI_ISL_490288, EPI_ISL_490289, EPI_ISL_490290, EPI_ISL_490291, EPI_ISL_490292, EPI_ISL_490293, EPI_ISL_490294, EPI_ISL_490295, EPI_ISL_490296, EPI_ISL_490297, EPI_ISL_490298, EPI_ISL_490299, EPI_ISL_490300, EPI_ISL_490301, EPI_ISL_490302, EPI_ISL_490303, EPI_ISL_490304, EPI_ISL_490305, EPI_ISL_490306, EPI_ISL_490307, EPI_ISL_490308, EPI_ISL_490309, EPI_ISL_490310, EPI_ISL_490311, EPI_ISL_490312, EPI_ISL_490313, EPI_ISL_490314, EPI_ISL_504186, EPI_ISL_504187, EPI_ISL_504188, EPI_ISL_504189, EPI_ISL_504190, EPI_ISL_504191, EPI_ISL_504192, EPI_ISL_504193, EPI_ISL_504194, EPI_ISL_504195, EPI_ISL_504196, EPI_ISL_504197, EPI_ISL_504198, EPI_ISL_504199, EPI_ISL_504200, EPI_ISL_504201, EPI_ISL_504202, EPI_ISL_504203, EPI_ISL_504204, EPI_ISL_504205, EPI_ISL_504206, EPI_ISL_504207, EPI_ISL_504208, EPI_ISL_504209, EPI_ISL_504210, EPI_ISL_504211, EPI_ISL_504212, EPI_ISL_504213, EPI_ISL_504214, EPI_ISL_504215, EPI_ISL_504216, EPI_ISL_504217, EPI_ISL_504218, EPI_ISL_504219, EPI_ISL_504220, EPI_ISL_504221, EPI_ISL_504222, EPI_ISL_504223, EPI_ISL_504224, EPI_ISL_504225, EPI_ISL_504226, EPI_ISL_504227, EPI_ISL_504228, EPI_ISL_504229, EPI_ISL_504230, EPI_ISL_504231, EPI_ISL_504232, EPI_ISL_504233, EPI_ISL_504234, EPI_ISL_504235, EPI_ISL_504236, EPI_ISL_504237, EPI_ISL_504238, EPI_ISL_504239, EPI_ISL_504240, EPI_ISL_504241, EPI_ISL_504242, EPI_ISL_504243, EPI_ISL_504244, EPI_ISL_514355, EPI_ISL_514356, EPI_ISL_514357, EPI_ISL_514358, EPI_ISL_514359, EPI_ISL_514360, EPI_ISL_514361, EPI_ISL_514362, EPI_ISL_514363, EPI_ISL_514364, EPI_ISL_514365, EPI_ISL_514366, EPI_ISL_514367, EPI_ISL_514368, EPI_ISL_514369, EPI_ISL_514370, EPI_ISL_514371, EPI_ISL_514372, EPI_ISL_514373, EPI_ISL_514374, EPI_ISL_514375, EPI_ISL_514376, EPI_ISL_514377, EPI_ISL_514378, EPI_ISL_514379, EPI_ISL_514380, EPI_ISL_514381, EPI_ISL_514382, EPI_ISL_514383, EPI_ISL_514384, EPI_ISL_514385, EPI_ISL_514386, EPI_ISL_514387, EPI_ISL_514388, EPI_ISL_514389, EPI_ISL_514390, EPI_ISL_514391, EPI_ISL_514392, EPI_ISL_514393, EPI_ISL_514394, EPI_ISL_514395, EPI_ISL_514396, EPI_ISL_514397, EPI_ISL_514398, EPI_ISL_514399, EPI_ISL_514400, EPI_ISL_514401, EPI_ISL_514402, EPI_ISL_514403, EPI_ISL_514404, EPI_ISL_514405, EPI_ISL_514406, EPI_ISL_514407, EPI_ISL_514408, EPI_ISL_514409, EPI_ISL_514410, EPI_ISL_514411, EPI_ISL_514412, EPI_ISL_514413, EPI_ISL_514414, EPI_ISL_514415, EPI_ISL_514416, EPI_ISL_514417, EPI_ISL_514418, EPI_ISL_514419, EPI_ISL_514420, EPI_ISL_514421, EPI_ISL_514422, EPI_ISL_514423, EPI_ISL_514424, EPI_ISL_515133, EPI_ISL_515134, EPI_ISL_515135, EPI_ISL_515136, EPI_ISL_515137, EPI_ISL_515138, EPI_ISL_515139, EPI_ISL_515140, EPI_ISL_515141, EPI_ISL_515142, EPI_ISL_515143, EPI_ISL_515144, EPI_ISL_515145, EPI_ISL_515146, EPI_ISL_515147, EPI_ISL_515148, EPI_ISL_515149, EPI_ISL_515150, EPI_ISL_515151, EPI_ISL_515152, EPI_ISL_515153, EPI_ISL_515154, EPI_ISL_515155, EPI_ISL_515156, EPI_ISL_515157, EPI_ISL_515158, EPI_ISL_515159, EPI_ISL_515160, EPI_ISL_515161, EPI_ISL_515162, EPI_ISL_515163, EPI_ISL_515164, EPI_ISL_515165, EPI_ISL_515166, EPI_ISL_515167, EPI_ISL_515168, EPI_ISL_515169, EPI_ISL_515170, EPI_ISL_515171, EPI_ISL_515172, EPI_ISL_515173, EPI_ISL_515174, EPI_ISL_515175, EPI_ISL_515176, EPI_ISL_515177, EPI_ISL_515178, EPI_ISL_515179, EPI_ISL_515180, EPI_ISL_622894, EPI_ISL_622895, EPI_ISL_622896, EPI_ISL_622897, EPI_ISL_622898, EPI_ISL_622899, EPI_ISL_622900, EPI_ISL_622901, EPI_ISL_622902, EPI_ISL_622903, EPI_ISL_622904, EPI_ISL_622905, EPI_ISL_622906, EPI_ISL_622907, EPI_ISL_622908, EPI_ISL_622909, EPI_ISL_622910, EPI_ISL_622911, EPI_ISL_622912, EPI_ISL_622913, EPI_ISL_622914, EPI_ISL_622915, EPI_ISL_622916, EPI_ISL_622917, EPI_ISL_622918, EPI_ISL_622919, EPI_ISL_622920, EPI_ISL_622921, EPI_ISL_622922, EPI_ISL_622923, EPI_ISL_622924, EPI_ISL_622925, EPI_ISL_622926, EPI_ISL_622927, EPI_ISL_622928, EPI_ISL_622929, EPI_ISL_622930, EPI_ISL_622931, EPI_ISL_622932, EPI_ISL_622933, EPI_ISL_622934, EPI_ISL_622935, EPI_ISL_622936, EPI_ISL_622937, EPI_ISL_622938, EPI_ISL_622939, EPI_ISL_622940, EPI_ISL_622941, EPI_ISL_2433739, EPI_ISL_2433743, EPI_ISL_2433744, EPI_ISL_2433745, EPI_ISL_2433746, EPI_ISL_2433747, EPI_ISL_2433748, EPI_ISL_2433749, EPI_ISL_2433750, EPI_ISL_2433751, EPI_ISL_2433752, EPI_ISL_2433753, EPI_ISL_2433754, EPI_ISL_2433755, EPI_ISL_2433756, EPI_ISL_2433757, EPI_ISL_2433758, EPI_ISL_2433759, EPI_ISL_2433760, EPI_ISL_2433761, EPI_ISL_2433762, EPI_ISL_2433763, EPI_ISL_2433764, EPI_ISL_2433765, EPI_ISL_2433766, EPI_ISL_2433767, EPI_ISL_2433768, EPI_ISL_2433769, EPI_ISL_2433770, EPI_ISL_2433771, EPI_ISL_2433772, EPI_ISL_2433773, EPI_ISL_2433774, EPI_ISL_2433775, EPI_ISL_2433776, EPI_ISL_2433796, EPI_ISL_2433797, EPI_ISL_2433798, EPI_ISL_2433799, EPI_ISL_2433800, EPI_ISL_2433801, EPI_ISL_2433802, EPI_ISL_2433803, EPI_ISL_2433804, EPI_ISL_2433805, EPI_ISL_2433806, EPI_ISL_2433807, EPI_ISL_2433808, EPI_ISL_2433809, EPI_ISL_2433810, EPI_ISL_2433811, EPI_ISL_2433812, EPI_ISL_2433813, EPI_ISL_2433814, EPI_ISL_2433815, EPI_ISL_2433816, EPI_ISL_2433817, EPI_ISL_2433818, EPI_ISL_2433819, EPI_ISL_2433820, EPI_ISL_2433821, EPI_ISL_2433822, EPI_ISL_2433823, EPI_ISL_2433824, EPI_ISL_2433825, EPI_ISL_2433826, EPI_ISL_2433827, EPI_ISL_2433828, EPI_ISL_2433829, EPI_ISL_2433830, EPI_ISL_2433831, EPI_ISL_2433832, EPI_ISL_2433833, EPI_ISL_2433834, EPI_ISL_2433835, EPI_ISL_2433836, EPI_ISL_2433837, EPI_ISL_2433838, EPI_ISL_2433839, EPI_ISL_2433840, EPI_ISL_2433841, EPI_ISL_2433842, EPI_ISL_2433843, EPI_ISL_2433844, EPI_ISL_2433845, EPI_ISL_2433846, EPI_ISL_2433847, EPI_ISL_2433848, EPI_ISL_2433849, EPI_ISL_2444004, EPI_ISL_2444016, EPI_ISL_2444021, EPI_ISL_2444024, EPI_ISL_2444027, EPI_ISL_2444028, EPI_ISL_2444029, EPI_ISL_2444030, EPI_ISL_2444031, EPI_ISL_2444032, EPI_ISL_2444033, EPI_ISL_2444034, EPI_ISL_2444035, EPI_ISL_2444036, EPI_ISL_2444037, EPI_ISL_2444038, EPI_ISL_2444039, EPI_ISL_2444040, EPI_ISL_2444041, EPI_ISL_2444042, EPI_ISL_2444043, EPI_ISL_2444044, EPI_ISL_2444045, EPI_ISL_2444046, EPI_ISL_2444047, EPI_ISL_2444048, EPI_ISL_2444049, EPI_ISL_2444050, EPI_ISL_2444051, EPI_ISL_2444052, EPI_ISL_2444053, EPI_ISL_2444054, EPI_ISL_2444055, EPI_ISL_2444056, EPI_ISL_2444057, EPI_ISL_2444058, EPI_ISL_2444059, EPI_ISL_2444060, EPI_ISL_2444061, EPI_ISL_2444062, EPI_ISL_2444063, EPI_ISL_2444064, EPI_ISL_2444065, EPI_ISL_2444066, EPI_ISL_2444067, EPI_ISL_2444068, EPI_ISL_2444069, EPI_ISL_2444070, EPI_ISL_2444071, EPI_ISL_2444072, EPI_ISL_2444073, EPI_ISL_2444074, EPI_ISL_2444075, EPI_ISL_2444076, EPI_ISL_2444078, EPI_ISL_2444079, EPI_ISL_2444080, EPI_ISL_2444081, EPI_ISL_2444082, EPI_ISL_2444083, EPI_ISL_2444084, EPI_ISL_2444085, EPI_ISL_2444086, EPI_ISL_2444087, EPI_ISL_2444088, EPI_ISL_2444089, EPI_ISL_2444090, EPI_ISL_2444091, EPI_ISL_2444092, EPI_ISL_2444093			

see above	National Institute for Communicable Diseases of the National Health Laboratory Service	National Institute for Communicable Diseases of the National Health Laboratory Service	Allam M; Amoako DG; Bhiman JN; Ismail A; Khumalo Z; Kwenda S; Mahlangu B; Mnyameni F; Mohale T; Mtshali P; Ntuli N; Scheepers C; Subramoney K
EPI_ISL_2107094, EPI_ISL_2107095, EPI_ISL_2107099, EPI_ISL_2308255	National Institute of Health (NIH) - Federal Government of Somalia	African Centre of Excellence for Genomics of Infectious Diseases (ACEGID), Redeemer's University	A.T.; Abechi; Ajogbasile; Akano; C.A.; C.T.; Eromon; F.V.; Folarin, O.; Happi; I.B.; J.N.; J.U.; K.O.; Kayode; Nosamiefan, I.; Oguzie; Olawoye; Olumade; Oluniyi; P.E.; P.S.; T.J.; Ugwu; Uwanibe
EPI_ISL_644739, EPI_ISL_644741, EPI_ISL_644742, EPI_ISL_644745, EPI_ISL_644746, EPI_ISL_644747, EPI_ISL_644748, EPI_ISL_644749, EPI_ISL_644754, EPI_ISL_644755, EPI_ISL_644759, EPI_ISL_644760, EPI_ISL_644761, EPI_ISL_644767, EPI_ISL_644768, EPI_ISL_644769, EPI_ISL_644771, EPI_ISL_644775, EPI_ISL_644776, EPI_ISL_644777, EPI_ISL_644778, EPI_ISL_644779, EPI_ISL_644782, EPI_ISL_644812, EPI_ISL_644813, EPI_ISL_644814, EPI_ISL_644815, EPI_ISL_644816, EPI_ISL_644817, EPI_ISL_644818, EPI_ISL_644819, EPI_ISL_644820, EPI_ISL_644821, EPI_ISL_647970, EPI_ISL_647971, EPI_ISL_647972, EPI_ISL_647973, EPI_ISL_647974, EPI_ISL_647975, EPI_ISL_647983, EPI_ISL_647984, EPI_ISL_1191782, EPI_ISL_1191783, EPI_ISL_1191784, EPI_ISL_1191785, EPI_ISL_1191786, EPI_ISL_1191787, EPI_ISL_1191788, EPI_ISL_1191789, EPI_ISL_1191790, EPI_ISL_1191791, EPI_ISL_1191792, EPI_ISL_1191793, EPI_ISL_1191794, EPI_ISL_1191795, EPI_ISL_1191796, EPI_ISL_1191797, EPI_ISL_1191798, EPI_ISL_1191799, EPI_ISL_1191800, EPI_ISL_1191801, EPI_ISL_1191802, EPI_ISL_1191803, EPI_ISL_1191804, EPI_ISL_1191805, EPI_ISL_1191806, EPI_ISL_1191807, EPI_ISL_1191808, EPI_ISL_1191809, EPI_ISL_1191810, EPI_ISL_1191811, EPI_ISL_1191812, EPI_ISL_1191813, EPI_ISL_1191814, EPI_ISL_1191815, EPI_ISL_1191816, EPI_ISL_1191817, EPI_ISL_1191818, EPI_ISL_1191819, EPI_ISL_1191820, EPI_ISL_1191821, EPI_ISL_1191822, EPI_ISL_1191823, EPI_ISL_1195298, EPI_ISL_1195300			
see above	National Microbiology Reference Laboratory	Quadram Institute Bioscience	Agnes Juru; Alexander Goredema; Ana-Victoria Gutierrez; Andrew J. Page; Andrew Tarupiwa; Barbra Murwira; Beuty Makamure; Charles Nyagupe; David Baker; Faustinos T Takawira; Gaetan Thilliez; Gemma Kay; Gibson Mhlanga; Hlanai Gumbo; Isaac Phiri; Justin O'Grady; Kenneth K Maeka; Leonardo de Oliveira Martins; Muchaneta Mugabe; Portia Manangazira; Raiva Simbi; Robert Kingsley; Sekesai Zinyowera; Tapfumanei Mashe; Tatenda Takawira; Thanh Le Viet
EPI_ISL_2492577	National Microbiology Reference Laboratory, Ministry of Health, Harare, Zimbabwe	CERI, Centre for Epidemic Response and Innvoation, Stellenbosch University and KRISP, KZN Research Innovation and Sequencing Platform, UKZN.	Agnes Juru; Air Comodor Dr J. Chimedza; Charles Nyagupe; Dr Raiva Simbi; Emmanuel SJ; Giandhari J; Hlanai Gumbo; Kenneth Maeka; Naidoo Y; Pillay S; Tapfumanei Mashe; Tatenda Takawira; Tegally H; Wilkinson E; de Oliveira T
EPI_ISL_845546, EPI_ISL_845547, EPI_ISL_845548, EPI_ISL_845549, EPI_ISL_845550, EPI_ISL_845551	National Public Health Laboratory, Cameroon	African Centre of Excellence for Genomics of Infectious Diseases (ACEGID), Redeemer's University	Oluniyi P.E. et al
EPI_ISL_737200, EPI_ISL_737205, EPI_ISL_737207	National Reference Laboratory, Nigeria Centre for Disease Control.	National Reference Laboratory, Nigeria Centre for Disease Control, Gaduwa,Abuja, Nigeria	(JHU); Abdulmajid Musa; Adama Ahmad; Anthony Ahumibe; Bamidele Olorunfemi; Catherine Okoi; Celestina Obiekea; Chimaobi Chukwu; Dr Adesuyi Omoare; Dr Chikwe Ihekweazu; Dr Ndodo Nnaemeka; Dr Sikiru Badaru; Kingsley Madubuike; Michael Popoola; Nneamaka Uba; Nwando Mba; Olusola Akanbi; Peter Van Heusden (SANBI); Shirlee Wohl
EPI_ISL_3845304, EPI_ISL_3869526, EPI_ISL_3869527, EPI_ISL_3869528, EPI_ISL_3869529, EPI_ISL_3869530, EPI_ISL_3869531, EPI_ISL_3869532, EPI_ISL_3869533, EPI_ISL_3869534, EPI_ISL_3869535, EPI_ISL_3869536, EPI_ISL_3869537, EPI_ISL_3920340, EPI_ISL_3920343			
see above	Naval Medical Research Unit No. 3	Naval Medical Research Center Biological Defense Research Directorate	Andrea E. Luquette; Andrew J. Bennett; Catherine E. Arnold; Chaselynn M. Watters; Emily K. Stefanov; Francisco Malagon; Kyle A. Long; Lindsay A. Glang; Logan J. Voegtly; Luis A. Estrella; Michael V. Deschenes; Regina Z. Cer; Robin H. Miller; Stephen M. Eggan; and Kimberly A. Bishop-Lilly
EPI_ISL_825633	Nigeria Centre For Disease Control	National reference Laboratory, NCDC, Gaduwa, Abuja	Anthony Ahumibe; Chimaobi Chukwu; Dr Chikwe Ihekweazu; Dr Ndodo Nnaemeka; Dr Omoare Adesuyi; Esebanmen Grace; Naidoo Dhamari; Nwando Mba; Olusola Anuoluwapo Akanbi
EPI_ISL_872601, EPI_ISL_872602, EPI_ISL_872603, EPI_ISL_872604	Nigeria Centre for Disease Control (NCDC)	African Centre of Excellence for Genomics of Infectious Diseases (ACEGID), Redeemer's University	Oluniyi P.E. et al
EPI_ISL_487106, EPI_ISL_527887, EPI_ISL_527888, EPI_ISL_527889, EPI_ISL_527890, EPI_ISL_527891, EPI_ISL_527892, EPI_ISL_527894, EPI_ISL_527895, EPI_ISL_527896, EPI_ISL_527897, EPI_ISL_527898, EPI_ISL_527899, EPI_ISL_527900, EPI_ISL_527901, EPI_ISL_527902, EPI_ISL_527903, EPI_ISL_527904, EPI_ISL_527905, EPI_ISL_527906, EPI_ISL_527907, EPI_ISL_527908, EPI_ISL_527909, EPI_ISL_527910, EPI_ISL_527911, EPI_ISL_527912, EPI_ISL_527913, EPI_ISL_527914, EPI_ISL_527915, EPI_ISL_729923, EPI_ISL_729924, EPI_ISL_729925, EPI_ISL_729927, EPI_ISL_729928, EPI_ISL_729929, EPI_ISL_729930, EPI_ISL_729931, EPI_ISL_729932, EPI_ISL_729933, EPI_ISL_729934, EPI_ISL_729935, EPI_ISL_729951, EPI_ISL_729952, EPI_ISL_729953, EPI_ISL_729954, EPI_ISL_729955, EPI_ISL_729956, EPI_ISL_729957, EPI_ISL_729958, EPI_ISL_729966, EPI_ISL_729967, EPI_ISL_729968, EPI_ISL_729969, EPI_ISL_729970, EPI_ISL_729971, EPI_ISL_729972, EPI_ISL_729973, EPI_ISL_729974, EPI_ISL_729975, EPI_ISL_729976, EPI_ISL_729977, EPI_ISL_729978, EPI_ISL_729979, EPI_ISL_729980, EPI_ISL_729981, EPI_ISL_729986, EPI_ISL_729987, EPI_ISL_729988, EPI_ISL_729989, EPI_ISL_729990, EPI_ISL_729991, EPI_ISL_729992, EPI_ISL_729993, EPI_ISL_729994, EPI_ISL_730017, EPI_ISL_730018, EPI_ISL_730019, EPI_ISL_730020, EPI_ISL_730021, EPI_ISL_730022, EPI_ISL_730024, EPI_ISL_730025, EPI_ISL_730026, EPI_ISL_730027, EPI_ISL_730042, EPI_ISL_730043, EPI_ISL_730044, EPI_ISL_730045, EPI_ISL_730046, EPI_ISL_730047, EPI_ISL_730048			
see above	Nigeria Centre for Disease Control (NCDC)	African Centre of Excellence for Genomics of Infectious Diseases (ACEGID), Redeemer's University, Ede, Osun State, Nigeria	Ajogbasile F.V.; Folarin O.A.; Happi C.T.; Ihekweazu C.; Kayode A.; Oguzie J.; Olawoye I.; Olumade T.; Oluniyi P.E.; Oluniyi P.E. et al; Uwanibe J.
EPI_ISL_735438	Nuclei Acid Testing - Rwanda National Reference Laboratory	GIGA Medical Genomics	Bouchra Boujemla; Esperence Umumararungu; Jacob Souopgui; Keith Durkin; Léon Mutesa; Maria Artesi; Marie-Pierre Hayette; Patrick Tuyisenge; Robert Rutayisire; Sabin Nsanzimana; Swaibu Gatare; Sébastien Bontems; Vincent Bours; Yvan Butera
EPI_ISL_735436, EPI_ISL_735437	Nucleic Acid Testing - Rwanda National Reference Laboratory	GIGA Medical Genomics	Bouchra Boujemla; Esperence Umumararungu; Jacob Souopgui; Keith Durkin; Léon Mutesa; Maria Artesi; Marie-Pierre Hayette; Patrick Tuyisenge; Robert Rutayisire; Sabin Nsanzimana; Swaibu Gatare; Sébastien Bontems; Vincent Bours; Yvan Butera
EPI_ISL_960227, EPI_ISL_960228, EPI_ISL_960229, EPI_ISL_960230, EPI_ISL_960231, EPI_ISL_960232, EPI_ISL_960233, EPI_ISL_960234, EPI_ISL_960235, EPI_ISL_960238, EPI_ISL_960239, EPI_ISL_960240, EPI_ISL_960241, EPI_ISL_960242, EPI_ISL_960243, EPI_ISL_960244, EPI_ISL_960245, EPI_ISL_960246, EPI_ISL_960247, EPI_ISL_960248, EPI_ISL_960249, EPI_ISL_960250, EPI_ISL_960251, EPI_ISL_960252, EPI_ISL_960253, EPI_ISL_960254, EPI_ISL_960255, EPI_ISL_960256, EPI_ISL_960257, EPI_ISL_960258, EPI_ISL_960259, EPI_ISL_960260, EPI_ISL_960261, EPI_ISL_960262, EPI_ISL_960263, EPI_ISL_960264, EPI_ISL_960265, EPI_ISL_960266, EPI_ISL_960267, EPI_ISL_960268, EPI_ISL_960269, EPI_ISL_960270, EPI_ISL_960271, EPI_ISL_960272, EPI_ISL_960273, EPI_ISL_960274, EPI_ISL_960275, EPI_ISL_960276, EPI_ISL_960277, EPI_ISL_960278, EPI_ISL_960279, EPI_ISL_960280, EPI_ISL_960281, EPI_ISL_960282, EPI_ISL_960283, EPI_ISL_960284, EPI_ISL_960285, EPI_ISL_960286, EPI_ISL_960287, EPI_ISL_960288, EPI_ISL_960289, EPI_ISL_960290, EPI_ISL_960291, EPI_ISL_960292, EPI_ISL_960293, EPI_ISL_960294, EPI_ISL_960295, EPI_ISL_960296, EPI_ISL_960297, EPI_ISL_960298, EPI_ISL_960299, EPI_ISL_960300, EPI_ISL_960301			
see above	Nucleic Acid Testing, National Reference Laboratory	GIGA Medical Genomics	Bouchra Boujemla; Corinne Fasquelle; Esperence Umumararungu; Jacob Souopgui; Keith Durkin; Léon Mutesa; Maria Artesi; Marie-Pierre Hayette; Nathalie Renotte; Patrick Tuyisenge; Robert Rutayisire; Sabin Nsanzimana; Swaibu Gatare; Sébastien Bontems; Vincent Bours; Yvan Butera
EPI_ISL_640017, EPI_ISL_640023, EPI_ISL_640077, EPI_ISL_700417, EPI_ISL_700447, EPI_ISL_700575, EPI_ISL_700594, EPI_ISL_2140665, EPI_ISL_2375943, EPI_ISL_2621083, EPI_ISL_2621089			
see above	Oudtshoorn Hospital wc OUD	NHLS/UCT	Arash Iranzadeh; Bruna Galvao; Carolyn Williamson; Deelan Doolabh; Diana Hardie; Gert Marais; Innocent Mudau; Kruger Marais; Lynn Tyers; Marvin Hsiao; Stephen Korsman
EPI_ISL_2621090	Oudtshoorn Sick Bay (SANDF)	NHLS/UCT	Arash Iranzadeh; Bruna Galvao; Carolyn Williamson; Deelan Doolabh; Diana Hardie; Gert Marais; Innocent Mudau; Lynn Tyers; Marvin Hsiao; Stephen Korsman
EPI_ISL_700586, EPI_ISL_2802109	Pacaltsdorp Clinic wc PAC	NHLS/UCT	Arash Iranzadeh; Bruna Galvao; Carolyn Williamson; Deelan Doolabh; Diana Hardie; Gert Marais; Innocent Mudau; Kruger Marais; Lynn Tyers; Marvin Hsiao; Stephen Korsman
EPI_ISL_700528	Plettenberg Bay Clinic wc PLC	NHLS/UCT	Arash Iranzadeh; Bruna Galvao; Carolyn Williamson; Deelan Doolabh; Diana Hardie; Innocent Mudau; Kruger Marais; Lynn Tyers; Marvin Hsiao; Stephen Korsman
EPI_ISL_2779418, EPI_ISL_2779419, EPI_ISL_2779420, EPI_ISL_2779424, EPI_ISL_2779425, EPI_ISL_2779426, EPI_ISL_2779427, EPI_ISL_2779428, EPI_ISL_2779429, EPI_ISL_2779430			
see above	Port Health Clinic, Busia border point	USAMRD-A, Basic Science Laboratory	Alan Lemtudo; Beth Mutai; Brian Andika; Carol Kifude; Clement Masakwe; Eric Muthanje; Esther Omuseni; Faith Sigei; Gathii Kimita; George Awinda; John Waitumbi; Josphat Nyataya; Rachel Githii; Rehema Liyai; Stephen Ochola
EPI_ISL_2779492, EPI_ISL_2779493, EPI_ISL_2779496, EPI_ISL_2779497, EPI_ISL_2779498, EPI_ISL_2779499, EPI_ISL_2779500, EPI_ISL_2779501, EPI_ISL_2779502, EPI_ISL_2779503, EPI_ISL_2779504, EPI_ISL_2779505, EPI_ISL_2779545			
see above	Port Health Clinic, Malaba border point	USAMRD-A, Basic Science Laboratory	Alan Lemtudo; Beth Mutai; Brian Andika; Carol Kifude; Clement Masakwe; Eric Muthanje; Esther Omuseni; Faith Sigei; Gathii Kimita; George Awinda; John Waitumbi; Josphat Nyataya; Rachel Githii; Rehema Liyai; Stephen Ochola
EPI_ISL_640086, EPI_ISL_640136	Red Cross Children's Hospital wc RXH	NHLS/UCT	Arash Iranzadeh; Bruna Galvao; Carolyn Williamson; Deelan Doolabh; Diana Hardie; Innocent Mudau; Kruger Marais; Lynn Tyers; Marvin Hsiao; Stephen Korsman
EPI_ISL_640035, EPI_ISL_700465	Riversdale Clinic wc RAV	NHLS/UCT	Arash Iranzadeh; Bruna Galvao; Carolyn Williamson; Deelan Doolabh; Diana Hardie; Innocent Mudau; Kruger Marais; Lynn Tyers; Marvin Hsiao; Stephen Korsman
EPI_ISL_2285240, EPI_ISL_2285241, EPI_ISL_2285242, EPI_ISL_2285243, EPI_ISL_2285244	Rob Ferreira	National Institute for Communicable Diseases of the National Health Laboratory Service	Amoako DG; Bhiman JN; Ismail A; Mahlangu B; Mohale T; Ntuli N; Scheepers C
EPI_ISL_615069	Rwanda National Reference Laboratory	Rwanda National Laboratory	Aine O'Toole; Mukantwari Enatha; Stefan Rooke; Umuringa Jeanne d'Arc
EPI_ISL_614763, EPI_ISL_614891, EPI_ISL_614892, EPI_ISL_614980, EPI_ISL_615063, EPI_ISL_615064, EPI_ISL_615066, EPI_ISL_615067, EPI_ISL_615071, EPI_ISL_615074, EPI_ISL_615075, EPI_ISL_707711, EPI_ISL_707712, EPI_ISL_707713, EPI_ISL_707771, EPI_ISL_707772, EPI_ISL_707773, EPI_ISL_707774, EPI_ISL_707776, EPI_ISL_707777, EPI_ISL_707779, EPI_ISL_707780, EPI_ISL_707783, EPI_ISL_707787, EPI_ISL_707788, EPI_ISL_707790			
see above	Rwanda National Reference Laboratory	Rwanda National Reference Laboratory	AIne O'Toole; Aine O'Toole; ENatha MUkantwari; Enatha Mukantwari; Jeanne d'Arc Mukantwari; Jeanne d'Arc UMuringa; Jeanne d'Arc Umuringa; MUkanntwari Eanatha; MUkantwari Enatha; Mukantwari Enatha; Stefan Rook; Stefan Rooke; UMuringa Jeanne d'Arc; Umuringa Jeanne d'Arc
EPI_ISL_640034	Sedgefield Clinic wc SGE	NHLS/UCT	Arash Iranzadeh; Bruna Galvao; Carolyn Williamson; Deelan Doolabh; Diana Hardie; Innocent Mudau; Kruger Marais; Lynn Tyers; Marvin Hsiao; Stephen Korsman
EPI_ISL_4274021	Service des Maladies Infectieuses	Northwestern University - Center for Pathogen Genomics and Microbial Evolution	Egon A. Ozer; Judd F. Hultquist; Lacy M. Simons; Mamadou S. Sow; Mamoudou Maiga; Ramon Lorenzo-Redondo
EPI_ISL_2928025	South Sudan Ministry of Health, WHO South Sudan, MRC/UVRI & LSHTM Uganda Research Unit	MRC/UVRI & LSHTM Uganda Research Unit, South Sudan Ministry of Health, WHO South Sudan	Abe G. Abias; Dan Lule Bugembe; Dennis Kenyi Lodiongo; James Ayei; John Rumunu; Joseph Francis Wamala; Juma John HM; Lul Lojok Deng; Matthew Cotten; My V.T. Phan; Pontiano Kaleebu; Richard Lino Loro Lako; Sudhir Bunga
EPI_ISL_640079	Stellenbosch Hospital wc STB	NHLS/UCT	Arash Iranzadeh; Bruna Galvao; Carolyn Williamson; Deelan Doolabh; Diana Hardie; Innocent Mudau; Kruger Marais; Lynn Tyers; Marvin Hsiao; Stephen Korsman
EPI_ISL_2285275, EPI_ISL_2285276, EPI_ISL_2285277, EPI_ISL_2285278, EPI_ISL_2285279, EPI_ISL_2285281, EPI_ISL_2285282, EPI_ISL_2285283, EPI_ISL_2285284, EPI_ISL_2285285, EPI_ISL_2285286, EPI_ISL_2285287, EPI_ISL_2285288, EPI_ISL_2285289, EPI_ISL_2285290, EPI_ISL_2285291, EPI_ISL_2285292, EPI_ISL_2285293, EPI_ISL_2285294, EPI_ISL_2285295, EPI_ISL_2285296, EPI_ISL_2285297, EPI_ISL_2285298, EPI_ISL_2285299, EPI_ISL_2285300, EPI_ISL_2285301, EPI_ISL_2285302, EPI_ISL_2285303			
see above	TSHWANE ACADEMIC LABORATORY	National Institute for Communicable Diseases of the National Health Laboratory Service	Amoako DG; Bhiman JN; Ismail A; Mahlangu B; Mohale T; Ntuli N; Scheepers C
EPI_ISL_966940	Technical Support Units for Scientific Research (UATRS), National Centre for Scientific and Technical Research (CNRST)	Technical Support Units for Scientific Research (UATRS), National Centre for Scientific and Technical Research (CNRST)	Alaoui; Elalaoui; Elannaz, H.; Elouanass, M.; Ennibi; H. and El Fahime, E.; Hemlali, M.; Lahlou; M.A.; Melloul, M.; Rfaki, A.; S.A.; Touil, N.; a.l.
EPI_ISL_640019, EPI_ISL_640026	Thembalethu CDC wc THC	NHLS/UCT	Arash Iranzadeh; Bruna Galvao; Carolyn Williamson; Deelan Doolabh; Diana Hardie; Innocent Mudau; Kruger Marais; Lynn Tyers; Marvin Hsiao; Stephen Korsman
EPI_ISL_2271958, EPI_ISL_2271959, EPI_ISL_2271960, EPI_ISL_2271961, EPI_ISL_2271962, EPI_ISL_2271963, EPI_ISL_2271969, EPI_ISL_2271975			
see above	Tshepong Hospital	National Institute for Communicable Diseases of the National Health Laboratory Service	Amoako DG; Bhiman JN; Ismail A; Mahlangu B; Mohale T; Ntuli N; Scheepers C
EPI_ISL_2447874	Tshwane Academic Laboratory	National Institute for Communicable Diseases of the National Health Laboratory Service	Amoako DG; Bhiman JN; Ismail A; Mahlangu B; Mohale T; Ntuli N; Scheepers C
EPI_ISL_960152	Tshwaragano Hospital	National Health Laboratory Service/UCT	Arash Iranzadeh; Bruna Galvao; Carolyn Williamson; Deelan Doolabh; Diana Hardie; Innocent Mudau; Kruger Marais; Lynn Tyers; Marvin Hsiao; Stephen Korsman
EPI_ISL_628750, EPI_ISL_628751, EPI_ISL_628752, EPI_ISL_628753, EPI_ISL_628754, EPI_ISL_628755, EPI_ISL_628756, EPI_ISL_628758, EPI_ISL_628759, EPI_ISL_628760, EPI_ISL_628761, EPI_ISL_648124, EPI_ISL_648125, EPI_ISL_648126, EPI_ISL_648127, EPI_ISL_648128			
see above	UHAS COVID-19 Lab	UHAS COVID-19 Lab	John O. Gyapong and the UHAS COVID-19 Lab Team; Jones Gyamfi; Kwabena O. Duedu; Reuben Ayivor-Djanie
EPI_ISL_737931, EPI_ISL_737939, EPI_ISL_737940, EPI_ISL_737941, EPI_ISL_737942, EPI_ISL_737943, EPI_ISL_737944, EPI_ISL_737945, EPI_ISL_737965, EPI_ISL_737971, EPI_ISL_737972, EPI_ISL_737973, EPI_ISL_737974, EPI_ISL_737975, EPI_ISL_737976, EPI_ISL_737977, EPI_ISL_737978, EPI_ISL_737979, EPI_ISL_737980, EPI_ISL_737981, EPI_ISL_737982, EPI_ISL_737983, EPI_ISL_737985, EPI_ISL_737986, EPI_ISL_737987, EPI_ISL_737988, EPI_ISL_737989, EPI_ISL_737990, EPI_ISL_737991, EPI_ISL_737992, EPI_ISL_737993, EPI_ISL_737994, EPI_ISL_737995, EPI_ISL_737997, EPI_ISL_737998, EPI_ISL_737999, EPI_ISL_738000, EPI_ISL_738001, EPI_ISL_738002, EPI_ISL_738003, EPI_ISL_738004, EPI_ISL_738005, EPI_ISL_738006, EPI_ISL_738007, EPI_ISL_738008, EPI_ISL_738009, EPI_ISL_738010, EPI_ISL_738011, EPI_ISL_738012, EPI_ISL_738013, EPI_ISL_738014, EPI_ISL_738015, EPI_ISL_738016, EPI_ISL_738017, EPI_ISL_738018, EPI_ISL_738019, EPI_ISL_738020, EPI_ISL_738021, EPI_ISL_738022, EPI_ISL_738023, EPI_ISL_738025, EPI_ISL_738026, EPI_ISL_738027, EPI_ISL_738028, EPI_ISL_738029, EPI_ISL_738030, EPI_ISL_738031, EPI_ISL_738032, EPI_ISL_738033, EPI_ISL_738034, EPI_ISL_738035, EPI_ISL_738036, EPI_ISL_738037			
see above	Uganda Central Public Health Lab and Uganda Virus Research Institute	MRC/UVRI & LSHTM Uganda Research Unit	Dan Lule Bugembe; Matthew Cotten; My V.T. Phan; Pontiano Kaleebu et al.
EPI_ISL_2243223, EPI_ISL_2243408	University of Maiduguri Teaching Hospital	International Centre for Genetic Engineering and Biotechnology (ICGEB) and ARGO Open Lab for Genome Sequencing	Alessandro Marcello; Bamidele S Oderinde; Danilo Licastro; Emanuele Orsini; Galadima Gadzama; Marycelin M Baba; Monilade Akinola; Simeone Dal Monego; Zara Wuduri

EPI_ISL_2243550	University of Maiduguri Teaching Hospital, P.M.B. 1414	International Centre for Genetic Engineering and Biotechnology (ICGEB) and ARGO Open Lab for Genome Sequencing	Alessandro Marcello; Bamidele S Oderinde; Danilo Licastro; Emanuele Orsini; Galadima Gadzama; Marycelin M Baba; Monilade Akinola; Simeone Dal Monego; Zara Wuduri
EPI_ISL_1208967, EPI_ISL_1208968	University of Namibia (UNAM LAB)	National Institute for Communicable Diseases of the National Health Laboratory Service	Amoako DG; Bhiman JN; Ismail A; Konstantinus I; Mahlangu B; Mohale T; Ntuli N; Scheepers C; Van Rooyen G
EPI_ISL_977251, EPI_ISL_977252, EPI_ISL_977253, EPI_ISL_977254, EPI_ISL_977255, EPI_ISL_977256, EPI_ISL_977260, EPI_ISL_977261, EPI_ISL_977262, EPI_ISL_977263, EPI_ISL_977264, EPI_ISL_977265, EPI_ISL_977266, EPI_ISL_977267, EPI_ISL_977268, EPI_ISL_977269, EPI_ISL_977271, EPI_ISL_977272, EPI_ISL_977273, EPI_ISL_977275, EPI_ISL_977277, EPI_ISL_977279, EPI_ISL_977280, EPI_ISL_977281, EPI_ISL_977282, EPI_ISL_977283, EPI_ISL_977284, EPI_ISL_977285, EPI_ISL_977293, EPI_ISL_977295, EPI_ISL_977296, EPI_ISL_977297, EPI_ISL_977299, EPI_ISL_977300, EPI_ISL_977301, EPI_ISL_977302, EPI_ISL_977303, EPI_ISL_977304, EPI_ISL_977305, EPI_ISL_977306, EPI_ISL_977307, EPI_ISL_977309, EPI_ISL_977310, EPI_ISL_977311, EPI_ISL_977312, EPI_ISL_977313, EPI_ISL_977314, EPI_ISL_977315, EPI_ISL_977316, EPI_ISL_977317, EPI_ISL_977318, EPI_ISL_977319, EPI_ISL_977320, EPI_ISL_977321, EPI_ISL_977322, EPI_ISL_977323, EPI_ISL_977324, EPI_ISL_977325, EPI_ISL_977326, EPI_ISL_977327, EPI_ISL_977328, EPI_ISL_977329, EPI_ISL_977330, EPI_ISL_977331, EPI_ISL_977332, EPI_ISL_977333, EPI_ISL_977335, EPI_ISL_977355, EPI_ISL_977359, EPI_ISL_977360, EPI_ISL_977361, EPI_ISL_977362, EPI_ISL_977363, EPI_ISL_977365, EPI_ISL_977368, EPI_ISL_977369, EPI_ISL_977370, EPI_ISL_977371, EPI_ISL_977372, EPI_ISL_977373, EPI_ISL_977374, EPI_ISL_977375, EPI_ISL_977377, EPI_ISL_977378, EPI_ISL_977379, EPI_ISL_977380, EPI_ISL_977381, EPI_ISL_977382, EPI_ISL_977383, EPI_ISL_977384, EPI_ISL_977385, EPI_ISL_977386, EPI_ISL_977387, EPI_ISL_977388, EPI_ISL_977389, EPI_ISL_977390, EPI_ISL_977391, EPI_ISL_977392, EPI_ISL_977393, EPI_ISL_977394, EPI_ISL_977395, EPI_ISL_977396, EPI_ISL_977397, EPI_ISL_977398, EPI_ISL_977399, EPI_ISL_977400, EPI_ISL_977401, EPI_ISL_977402, EPI_ISL_977403, EPI_ISL_977404, EPI_ISL_977405, EPI_ISL_977406, EPI_ISL_977407, EPI_ISL_977408, EPI_ISL_977409, EPI_ISL_977410, EPI_ISL_977412, EPI_ISL_977413, EPI_ISL_977414, EPI_ISL_977415, EPI_ISL_977416, EPI_ISL_977418, EPI_ISL_977419, EPI_ISL_977420, EPI_ISL_977423, EPI_ISL_977424, EPI_ISL_977425, EPI_ISL_977426, EPI_ISL_977427, EPI_ISL_977428, EPI_ISL_977429, EPI_ISL_977430, EPI_ISL_977431, EPI_ISL_977432, EPI_ISL_977433, EPI_ISL_977434, EPI_ISL_977435, EPI_ISL_977436, EPI_ISL_977437, EPI_ISL_977438, EPI_ISL_977439, EPI_ISL_977440, EPI_ISL_977441, EPI_ISL_977442, EPI_ISL_977443, EPI_ISL_977444, EPI_ISL_977445, EPI_ISL_977446, EPI_ISL_977447, EPI_ISL_977448, EPI_ISL_977449, EPI_ISL_977450, EPI_ISL_977451, EPI_ISL_977452, EPI_ISL_977453, EPI_ISL_977454, EPI_ISL_977455, EPI_ISL_977456, EPI_ISL_977457, EPI_ISL_977458, EPI_ISL_977460, EPI_ISL_977461, EPI_ISL_977462, EPI_ISL_977463, EPI_ISL_977464, EPI_ISL_977465, EPI_ISL_977466, EPI_ISL_2803318, EPI_ISL_2803319, EPI_ISL_2803320, EPI_ISL_2803321, EPI_ISL_2803322, EPI_ISL_2803323, EPI_ISL_2803324, EPI_ISL_2803325, EPI_ISL_2803326, EPI_ISL_2803327, EPI_ISL_2803328, EPI_ISL_2803329, EPI_ISL_2803330, EPI_ISL_2803331, EPI_ISL_2803332, EPI_ISL_2803333, EPI_ISL_2803334, EPI_ISL_2803335, EPI_ISL_2803336, EPI_ISL_2803337, EPI_ISL_2803338, EPI_ISL_2803339, EPI_ISL_2803340, EPI_ISL_2803341, EPI_ISL_2803342, EPI_ISL_2803343, EPI_ISL_2803344, EPI_ISL_2803345, EPI_ISL_2803346, EPI_ISL_2803366, EPI_ISL_2803367, EPI_ISL_2803368, EPI_ISL_2803369, EPI_ISL_2803370, EPI_ISL_2803371, EPI_ISL_2803372, EPI_ISL_2803373, EPI_ISL_2803374, EPI_ISL_2803375, EPI_ISL_2803376, EPI_ISL_2803377, EPI_ISL_2803378, EPI_ISL_2803379, EPI_ISL_2803380, EPI_ISL_2803381, EPI_ISL_2803382, EPI_ISL_2803383, EPI_ISL_2803384, EPI_ISL_2803385, EPI_ISL_2803386, EPI_ISL_2803387, EPI_ISL_2803388, EPI_ISL_2803389, EPI_ISL_2803390, EPI_ISL_2803391, EPI_ISL_2803392, EPI_ISL_2803393, EPI_ISL_2803394, EPI_ISL_2803395, EPI_ISL_2803396, EPI_ISL_2803397, EPI_ISL_2803398, EPI_ISL_2803399, EPI_ISL_2803400, EPI_ISL_2803401, EPI_ISL_2803402, EPI_ISL_2803403, EPI_ISL_2803404, EPI_ISL_2803405, EPI_ISL_2803406, EPI_ISL_2803407, EPI_ISL_2803408, EPI_ISL_2803409, EPI_ISL_2803410, EPI_ISL_2803411, EPI_ISL_2803412, EPI_ISL_2803413, EPI_ISL_2803414, EPI_ISL_2803415, EPI_ISL_2803416, EPI_ISL_2803417, EPI_ISL_2803418, EPI_ISL_2803419, EPI_ISL_2803420, EPI_ISL_2803421, EPI_ISL_2803422, EPI_ISL_2803423, EPI_ISL_2803427, EPI_ISL_2803428, EPI_ISL_2803429, EPI_ISL_2803430, EPI_ISL_2803431, EPI_ISL_2803432, EPI_ISL_2803433, EPI_ISL_2803434, EPI_ISL_2803435, EPI_ISL_2803436, EPI_ISL_2803438, EPI_ISL_2803439, EPI_ISL_2803440, EPI_ISL_2803441, EPI_ISL_2803442, EPI_ISL_2803443, EPI_ISL_2803444, EPI_ISL_2803445, EPI_ISL_2803446, EPI_ISL_2803447, EPI_ISL_2803452, EPI_ISL_2803455, EPI_ISL_2803456, EPI_ISL_2803457, EPI_ISL_2803458, EPI_ISL_2803459, EPI_ISL_2803460, EPI_ISL_2803461, EPI_ISL_2803462, EPI_ISL_2803463, EPI_ISL_2803464, EPI_ISL_2803465, EPI_ISL_2803466, EPI_ISL_2803467, EPI_ISL_2803468, EPI_ISL_2803469, EPI_ISL_2803470, EPI_ISL_2803471, EPI_ISL_2803472, EPI_ISL_2803473, EPI_ISL_2803474, EPI_ISL_2803475, EPI_ISL_2803476, EPI_ISL_2803477, EPI_ISL_2803478, EPI_ISL_2803479, EPI_ISL_2803480, EPI_ISL_2803481, EPI_ISL_2803482, EPI_ISL_2803483, EPI_ISL_2803484, EPI_ISL_2803485, EPI_ISL_2803486, EPI_ISL_2803487, EPI_ISL_2803488, EPI_ISL_2803489, EPI_ISL_2803490, EPI_ISL_2803491, EPI_ISL_2803492, EPI_ISL_2803493, EPI_ISL_2803494, EPI_ISL_2803495, EPI_ISL_2803496, EPI_ISL_2803497, EPI_ISL_2803498, EPI_ISL_2803499, EPI_ISL_2803500, EPI_ISL_2803501, EPI_ISL_2803502, EPI_ISL_2803503, EPI_ISL_2803504, EPI_ISL_2803505, EPI_ISL_2803506, EPI_ISL_2803507, EPI_ISL_2803508, EPI_ISL_2803509, EPI_ISL_2803510, EPI_ISL_2803511, EPI_ISL_2803512, EPI_ISL_2803513, EPI_ISL_2803525, EPI_ISL_2803526, EPI_ISL_2803527, EPI_ISL_2803528, EPI_ISL_2803529, EPI_ISL_2803530, EPI_ISL_2803532, EPI_ISL_2803534, EPI_ISL_2803535, EPI_ISL_2803536, EPI_ISL_2803537, EPI_ISL_2803538, EPI_ISL_2803539, EPI_ISL_2803540, EPI_ISL_2803541, EPI_ISL_2803542, EPI_ISL_2803543, EPI_ISL_2803544, EPI_ISL_2803545, EPI_ISL_2803546, EPI_ISL_2803547, EPI_ISL_2803548, EPI_ISL_2803549, EPI_ISL_2803550, EPI_ISL_2803551, EPI_ISL_2803552, EPI_ISL_2803553, EPI_ISL_2803554, EPI_ISL_2803555, EPI_ISL_2803556, EPI_ISL_2803557, EPI_ISL_2803560, EPI_ISL_2803561, EPI_ISL_2803562, EPI_ISL_2803580, EPI_ISL_2803611, EPI_ISL_2803612, EPI_ISL_2803613, EPI_ISL_2803614, EPI_ISL_2803615, EPI_ISL_2803618, EPI_ISL_2803619, EPI_ISL_2803620, EPI_ISL_2803621, EPI_ISL_2803622, EPI_ISL_2803623, EPI_ISL_2803624, EPI_ISL_2803625, EPI_ISL_2803626, EPI_ISL_2803627, EPI_ISL_2803628, EPI_ISL_2803633, EPI_ISL_2803634, EPI_ISL_2803640, EPI_ISL_2803641, EPI_ISL_2803642, EPI_ISL_2803643, EPI_ISL_2803644, EPI_ISL_2803649, EPI_ISL_2803650			
see above	University of Zambia, School of Veterinary Medicine	UNZAVET and PATH	Daniel Bridges; Mulenga Mwenda-Chimfwembe; Ngonda Saasa; ZNPHI and ZGSC
EPI_ISL_2494710, EPI_ISL_2494711, EPI_ISL_2494715, EPI_ISL_2494717, EPI_ISL_2494719, EPI_ISL_2494728, EPI_ISL_2494732, EPI_ISL_2494733, EPI_ISL_2494735, EPI_ISL_2494736, EPI_ISL_2494737, EPI_ISL_2494740, EPI_ISL_2494742, EPI_ISL_2494746, EPI_ISL_2494747, EPI_ISL_2494748, EPI_ISL_2494749, EPI_ISL_2494750, EPI_ISL_2494751, EPI_ISL_2494754, EPI_ISL_2494757, EPI_ISL_2494758, EPI_ISL_2494760, EPI_ISL_2494761, EPI_ISL_2494765, EPI_ISL_2494768, EPI_ISL_2494776, EPI_ISL_2494788, EPI_ISL_2494859, EPI_ISL_2494919, EPI_ISL_2494933, EPI_ISL_2494947, EPI_ISL_2494962, EPI_ISL_2494968, EPI_ISL_2494970, EPI_ISL_2494973, EPI_ISL_2494976, EPI_ISL_2494980, EPI_ISL_2494981, EPI_ISL_2494983, EPI_ISL_2494985, EPI_ISL_2494987, EPI_ISL_2494988, EPI_ISL_2494991, EPI_ISL_2494995, EPI_ISL_2495000, EPI_ISL_2495001, EPI_ISL_2495002, EPI_ISL_2495004, EPI_ISL_2495005, EPI_ISL_2495006, EPI_ISL_2495007, EPI_ISL_2495008, EPI_ISL_2495011, EPI_ISL_2495012, EPI_ISL_2495015, EPI_ISL_2495018, EPI_ISL_2495020, EPI_ISL_2495021, EPI_ISL_2495022, EPI_ISL_2495024, EPI_ISL_2495025, EPI_ISL_2495026, EPI_ISL_2495027, EPI_ISL_2495029, EPI_ISL_2495030, EPI_ISL_2495031, EPI_ISL_2495033, EPI_ISL_2495056			
see above	Vaccines and Infectious Diseases Analytics Research Unit (VIDA)	KRISP, KZN Research Innovation and Sequencing Platform	Baillie Vicky; Giandhari Jennifer; Madhi Shabir; Naidoo Yeshnee; Pillay Sureshnee; San James; Tegally Houriiyah; Wilkinson Eduan; de Oliveira Tulio; du Plessis Jeanine
EPI_ISL_2360399, EPI_ISL_2360402, EPI_ISL_2360403, EPI_ISL_2360404, EPI_ISL_2360974, EPI_ISL_2360975, EPI_ISL_2360976, EPI_ISL_2360979, EPI_ISL_2360980, EPI_ISL_2360986, EPI_ISL_2360987, EPI_ISL_2360988, EPI_ISL_2360989, EPI_ISL_2360990, EPI_ISL_2360991, EPI_ISL_2360992, EPI_ISL_2360993, EPI_ISL_2360994, EPI_ISL_2360995, EPI_ISL_2360996, EPI_ISL_2360997, EPI_ISL_2360998, EPI_ISL_2360999, EPI_ISL_2361000, EPI_ISL_2361001, EPI_ISL_2361002, EPI_ISL_2361004, EPI_ISL_2361021, EPI_ISL_2361022, EPI_ISL_2494371, EPI_ISL_2494373, EPI_ISL_2494378, EPI_ISL_2494379, EPI_ISL_2494380, EPI_ISL_2494381, EPI_ISL_2494388, EPI_ISL_2494389, EPI_ISL_2494391, EPI_ISL_2494392, EPI_ISL_2494393, EPI_ISL_2494396, EPI_ISL_2494398, EPI_ISL_2494406, EPI_ISL_2494418, EPI_ISL_2494425, EPI_ISL_2494426, EPI_ISL_2494434, EPI_ISL_2494439, EPI_ISL_2494456, EPI_ISL_2494466, EPI_ISL_2494468, EPI_ISL_2494479, EPI_ISL_2494501, EPI_ISL_2494512, EPI_ISL_2494553, EPI_ISL_2494562, EPI_ISL_2494564, EPI_ISL_2494567, EPI_ISL_2494577, EPI_ISL_2494580, EPI_ISL_2494588, EPI_ISL_2494589, EPI_ISL_2494591, EPI_ISL_2494593, EPI_ISL_2494594, EPI_ISL_2494598, EPI_ISL_2494601, EPI_ISL_2494603, EPI_ISL_2494616			
see above	Vaccines and Infectious Diseases Analytics Research Unit (VIDA)	KRISP, KZn Research Innovation and Sequencing Platform	Baillie Vicky; Giandhari Jennifer; Madhi Shabir; Naidoo Yeshnee; Pillay Sureshnee; San James; Tegally Houriiyah; Tshabuila Derek; Wilkinson Eduan; de Oliveira Tulio; du Plessis Jeanine
EPI_ISL_940851	Vaccines and Infectious Diseases Analytics Research Unit (VIDA)	KRISP, KZN Research Innovation and Sequencing Platform	Baillie Vicky; Giandhari Jennifer; Madhi Shabir; Naidoo Yeshnee; Pillay Sureshnee; Tegally Houriiyah; de Oliveira Tulio; du Plessis Jeanine
EPI_ISL_640037, EPI_ISL_640046, EPI_ISL_640114	Valkenberg Hospital wc VBH	NHLS/UCT	Arash Iranzadeh; Bruna Galvao; Carolyn Williamson; Deelan Doolabh; Diana Hardie; Innocent Mudau; Kruger Marais; Lynn Tyers; Marvin Hsiao; Stephen Korsman
EPI_ISL_640072	Vanguard CHC wc VGC	NHLS/UCT	Arash Iranzadeh; Bruna Galvao; Carolyn Williamson; Deelan Doolabh; Diana Hardie; Innocent Mudau; Kruger Marais; Lynn Tyers; Marvin Hsiao; Stephen Korsman
EPI_ISL_960159	Vanguard CHC wc VGC	National Health Laboratory Service/UCT	Arash Iranzadeh; Bruna Galvao; Carolyn Williamson; Deelan Doolabh; Diana Hardie; Innocent Mudau; Kruger Marais; Lynn Tyers; Marvin Hsiao; Stephen Korsman
EPI_ISL_640021, EPI_ISL_640032, EPI_ISL_640085, EPI_ISL_640108, EPI_ISL_640109, EPI_ISL_640110, EPI_ISL_640113, EPI_ISL_640126, EPI_ISL_700421, EPI_ISL_700546, EPI_ISL_700596, EPI_ISL_1040814			
see above	Victoria Hospital wc VHW	NHLS/UCT	Arash Iranzadeh; Bruna Galvao; Carolyn Williamson; Deelan Doolabh; Diana Hardie; Innocent Mudau; Kruger Marais; Lynn Tyers; Marvin Hsiao; Stephen Korsman
EPI_ISL_960150	Victoria Hospital wc VHW	National Health Laboratory Service/UCT	Arash Iranzadeh; Bruna Galvao; Carolyn Williamson; Deelan Doolabh; Diana Hardie; Innocent Mudau; Kruger Marais; Lynn Tyers; Marvin Hsiao; Stephen Korsman
EPI_ISL_591083, EPI_ISL_591084, EPI_ISL_591085, EPI_ISL_591086, EPI_ISL_2982621, EPI_ISL_2982622, EPI_ISL_2982623			
see above	Viral Respiratory Lab, National Institute for Biomedical Research (INRB)	Pathogen Sequencing Lab, National Institute for Biomedical Research (INRB)	Allison Black; Amuri Aziza; Andrew Rambaut; Catherine Pratt; Eddy Kinganda-Lusamaki; Edith Nkwembe; Emmanuel Lokilo Lofiko; Francisca Muyembe Mawete; Ian Goodfellow; James Hadfield; Jean Claude Makangara; Jean-Jacques Muyembe Tamfum; Josh Quick; Kristian Andersen; Matthias Pauthner; Michael Wiley; Nick Loman; Placide Mbala-Kingebeni; Steve Ahuka-Mundeke; Trevor Bedford
EPI_ISL_528386	Viral vaccines, VSVRI- Veterinary serum and vaccine research institute	Viral vaccines, VSVRI- Veterinary serum and vaccine research institute	A.A.; M.A.; Saad; Saleh
EPI_ISL_636987	Virology Lab, National Institute for Biomedical Research (INRB)	Project group Epidemology of Highly Pathogenic Microorganisms, Robert Koch-Institute	Eddy Kinganda-Lusamaki; Essia Belarbi; Fabian Leendertz; Gabriel Mbunsu; Grit Schubert; Jasmin Schlotterbeck; Jean-Jacques Muyembe Tamfum; Sheila Makiala; Steve Ahuka-Mundeke
EPI_ISL_677634, EPI_ISL_1660291	Virology Unit, Institut Pasteur de Madagascar	Virology Unit, Institut Pasteur de Madagascar	Cara E. Brook; Christian Ranaivoson; Cristina M. Tato; Helisoa Razafimanjato; Jean-Michel Heraud; Joseph L. DeRisi; Michelle Tan; Norosoa Razanajatovo; Philippe Dussart; Soa Fy Andriamandimby; Tsiry Randriambolamanantsoa; Vida Ahyong; Vololoniaina Raharinosy
EPI_ISL_2422499, EPI_ISL_2422500, EPI_ISL_2422514, EPI_ISL_2422520, EPI_ISL_2422521, EPI_ISL_2422522, EPI_ISL_2422523, EPI_ISL_2422524, EPI_ISL_2422525, EPI_ISL_2422526, EPI_ISL_2422527, EPI_ISL_2422528, EPI_ISL_2422529, EPI_ISL_2422530, EPI_ISL_2422531, EPI_ISL_2422532			
see above	West African Centre for Cell Biology of Infectious Pathogen, University of Ghana, Legon	WACCBIP, University of Ghana, Volta Road, Legon, Accra	Collins M. Morang'a; Dominic S. Y. Amuzu; Edward Danso Fenteng; Emmanuel Kudjo; Evelyn B. Quansah; Frederick Tei-Maya; Joe K. Mutungi; Joyce M. Ngoi; Lucas N. Amenga-Etego and Gordon A. Awandare; Nicaise T. Ndam; Patrick Tetteh Ababio; Peter K. Quashie; Philip M. Soglo; Samirah Saiid; Theophilus Odoom; Vincent Appiah; Violette M’cormack; William K. Ampofo; Yaw Bediako
EPI_ISL_1255106, EPI_ISL_1255107, EPI_ISL_1255108, EPI_ISL_1255109, EPI_ISL_1255110, EPI_ISL_1255111, EPI_ISL_1255112, EPI_ISL_1255113, EPI_ISL_1255114, EPI_ISL_1255115, EPI_ISL_1255116, EPI_ISL_1255117, EPI_ISL_1255118, EPI_ISL_1255119, EPI_ISL_1255142, EPI_ISL_1255143, EPI_ISL_1255144, EPI_ISL_1255145, EPI_ISL_1255146, EPI_ISL_1255147, EPI_ISL_1255148, EPI_ISL_1255149, EPI_ISL_1255150, EPI_ISL_1255151, EPI_ISL_1255152, EPI_ISL_1255153, EPI_ISL_1255154, EPI_ISL_1255155, EPI_ISL_1255156, EPI_ISL_1255186, EPI_ISL_1255228, EPI_ISL_1255242, EPI_ISL_1255243, EPI_ISL_1255244, EPI_ISL_1255245, EPI_ISL_1255246, EPI_ISL_1255247, EPI_ISL_1255248, EPI_ISL_1255249, EPI_ISL_1255250			
see above	West African Centre for Cell Biology of Infectious Pathogens (WACCBIP), University of Ghana, Accra, Ghana	West African Centre for Cell Biology of Infectious Pathogens (WACCBIP), University of Ghana, Volta Road, Legon-Accra, Ghana	; Abdoulaye B Diallo; Abdul-Karim Abass; Aisha Mohammed; Benjamin Demah Nuertey; Collins M. Morang'a; Dam Kenneth Mibut; Dominic S.Y. Amuzu; Emmanuella Amoako4; Evelyn B. Quansah; Frederick Kumi-Ansah; Frederick Tei-Maya; Gordon A Awandare; Joyce M. Ngoi; Kesego Tapela; Lucas N. Amenga-Etego; Nelson Kibinge; Oliver D Boakye5; Peter K Quashie; Philip M. Soglo; Samirah Saiid; Samuel Kaba Akoriyea; Theophilus Odoom; Vanessa Magnussen; Vincent Appiah; Yaw Bediako
EPI_ISL_1255105	West African Centre for Cell Biology of Infectious Pathogens (WACCBIP), University of Ghana, Volta Road, Legon-Accra, Ghana	West African Centre for Cell Biology of Infectious Pathogens (WACCBIP), University of Ghana, Volta Road, Legon-Accra, Ghana	; Abdoulaye B Diallo; Abdul-Karim Abass; Aisha Mohammed; Benjamin Demah Nuertey; Collins M. Morang'a; Dam Kenneth Mibut; Dominic S.Y. Amuzu; Emmanuella Amoako4; Evelyn B. Quansah; Frederick Kumi-Ansah; Frederick Tei-Maya; Gordon A Awandare; Joyce M. Ngoi; Kesego Tapela; Lucas N. Amenga-Etego; Nelson Kibinge; Oliver D Boakye5; Peter K Quashie; Philip M. Soglo; Samirah Saiid; Samuel Kaba Akoriyea; Theophilus Odoom; Vanessa Magnussen; Vincent Appiah; Yaw Bediako
EPI_ISL_700416	Zoar Clinic wc ZOA	NHLS/UCT	Arash Iranzadeh; Bruna Galvao; Carolyn Williamson; Deelan Doolabh; Diana Hardie; Innocent Mudau; Kruger Marais; Lynn Tyers; Marvin Hsiao; Stephen Korsman
