# Supplementary material for: Genomic Snapshot of SARS-CoV-2 in Migrants Entering Through Mediterranean Sea Routes
Source: Front Public Health. 2022 Mar 3;10:846115. doi: 10.3389/fpubh.2022.846115 (PMC8927662; doi:10.3389/fpubh.2022.846115)
Supplement: Supplementary file 2 [file Data_Sheet_2.PDF]

We gratefully acknowledge the following Authors from the Originating laboratories responsible for obtaining the specimens, as well as the Submitting laboratories where the genome data were generated and shared via GISAID, on which this research is based.

All Submitters of data may be contacted directly via www.gisaid.org

Authors are sorted alphabetically.

Accession ID	Originating Laboratory	Submitting Laboratory	Authors
EPI_ISL_457825	Army Medical Research Center - Scientific Department	Army Medical and Veterinary Research Center	Anna Anselmo; Antonella Fortunato; Florigio Lista; Francesco Giordani; Giovanni Faggioni; Nino D'Amore; Riccardo De Sanctis; Silvia Fillo; Vanessa Vera Fain
EPI_ISL_414599	Department of Surgical Sciences, University of Cagliari	Universita' di Cagliari	Coghe; DelRio; F. and Orru, G.; Fais, S.; Loddo, M.; N.M.C.; Palmieri, G.; Scano, A.; Scioscia, R.
EPI_ISL_965179	Laboratorio Biologia Molecolare SARS-COV2- UOC Laboratorio Analisi- Servizio Medicina di Laboratorio, Ospedale San Francesco - ATS-ASSLNUoro	LABORATORIO SPECIALISTICO UOC EMATOLOGIA- Ospedale San Francesco - ATS-ASSLNUoro	ASPRONI ROSANNA; FANCELLO TATIANA; FIAMMA MAURA; FLORE ANNA RITA. PALMAS ANGELO DOMENICO; IANA LO MAGLIO; MAMELI GIUSEPPE; MONNE MARIA ITRIA; PIRAS GIOVANNA; SANNA FILOMENA; SULIS VINCENZO; TOJA ALESSANDRO
EPI_ISL_458085	Laboratorio Biologia Molecolare Sars Cov2 - UOC Laboratorio Analisi - Servizio Medicina di Laboratorio , Ospedale "San Francesco" - ATS- ASSL Nuoro	Laboratorio specialistico UOC Ematologia - Ospedale "San Francesco" - ATS-ASSL Nuoro	Asproni Rosanna; Casu Gavino; Fancello Tatiana; Fiamma Maura; Floris Anna Rita; Lo Maglio Iana; Mameli Giuseppe.; Monne Maria Itria; Palmas Angelo Domenico; Piras Giovanna; Sanna Filomena; Sulis Vincenzo; Toja Alessandro
EPI_ISL_1085013	Laboratorio Biologia Molecolare Sars Cov2 - UOC Laboratorio Analisi - Servizio Medicina di Laboratorio, Ospedale "San Francesco" - ATS-ASSL Nuoro	Laboratorio Biologia Molecolare Sars Cov2 - UOC Laboratorio Analisi - Servizio Medicina di Laboratorio, Ospedale "San Francesco" - ATS-ASSL Nuoro	; Asproni Rosanna; Fiamma Maura; Lo Maglio Iana; Mameli Giuseppe; Monne Maria Itria; Palmas Angelo Domenico; Piras Giovanna
EPI_ISL_3770738	Laboratorio Biologia Molecolare Sars Cov2 - UOC Laboratorio Analisi - Servizio Medicina di Laboratorio, Ospedale "San Francesco" - ATS-ASSL Nuoro	Laboratorio Biologia Molecolare Sars Cov2 - UOC Laboratorio Analisi - Servizio Medicina di Laboratorio, Ospedale "San Francesco" - ATS-ASSL Nuoro and Laboratorio specialistico UOC Ematologia - Ospedale "San Francesco" - ATS-ASSL Nuoro	Asproni Rosanna; Carta Franco; Fancello Patrizia; Fiamma Maura; Garau Maria Cristina; Mameli Giuseppe; Palmas Angelo Domenico; Pira Giovanna; Piras Giovanna; Rosu Valentina
EPI_ISL_3664341, EPI_ISL_3664343, EPI_ISL_3664379	Laboratorio Biologia Molecolare Sars Cov2 - UOC Laboratorio Analisi - Servizio Medicina di Laboratorio, Ospedale "San Francesco" - ATS-ASSL Nuoro	Laboratorio Biologia Molecolare Sars Cov2 - UOC Laboratorio Analisi - Servizio Medicina di Laboratorio, Ospedale "San Francesco" - ATS-ASSL Nuoro and Laboratorio specialistico UOC Ematologia - Ospedale "San Francesco" - ATS-ASSL Nuoro	Asproni Rosanna; Carta Franco; Fancello Patrizia; Fiamma Maura; Garau Maria Cristina; Malune Paolo; Mameli Giuseppe; Palmas Angelo Domenico; Pira Giovanna; Piras Giovanna; Rosu Valentina
EPI_ISL_1173202	Laboratorio Biologia Molecolare Sars Cov2 - UOC Laboratorio Analisi - Servizio Medicina di Laboratorio, Ospedale "San Francesco" - ATS-ASSL Nuoro	Laboratorio Specialistico Ematologia, UOC Ematologia, Ospedale "San Francesco" - ATS-ASSL Nuoro	Asproni Rosanna; Carta Franco; Fancello Patrizia; Fiamma Maura; Lo Maglio Iana; Malune Paolo; Mameli Giuseppe; Monne Maria Itria; Palmas Angelo Domenico; Pira Giovanna; Piras Giovanna; Rosu Valentina; Serra Silvia
EPI_ISL_3527831	Laboratorio Biologia Molecolare Sars Cov2 - UOC Laboratorio Analisi - Servizio Medicina di Laboratorio, Ospedale "San Francesco" - ATS-ASSL Nuoro	Laboratorio Specialistico UOC Ematologia Ospedale "San Francesco" - ATS-ASSL Nuoro	Asproni Rosanna; Mameli Giuseppe; Monne Maria Itria; Palmas Angelo Domenico; Piras Giovanna
EPI_ISL_3527832	Laboratorio Biologia Molecolare Sars Cov2 - UOC Laboratorio Analisi - Servizio Medicina di Laboratorio, Ospedale "San Francesco" - ATS-ASSL Nuoro	Laboratorio Specialistico UOC Ematologia, Ospedale "San Francesco" - ATS-ASSL Nuoro	Asproni Rosanna; Fiamma Maura; Mameli Giuseppe; Monne Maria Itria; Palmas Angelo Domenico; Piras Giovanna
EPI_ISL_458084	Laboratorio Biologia Molecolare Sars Cov2 - UOC Laboratorio Analisi - Servizio Medicina di Laboratorio, Ospedale "San Francesco" - ATS-ASSL Nuoro	Laboratorio specialistico UOC Ematologia - Ospedale "San Francesco" - ATS-ASSL Nuoro	Asproni Rosanna; Casu Gavino; Fancello Tatiana; Fiamma Maura; Floris Anna Rita; Lo Maglio Iana; Mameli Giuseppe.; Monne Maria Itria; Palmas Angelo Domenico; Piras Giovanna; Sanna Filomena; Sulis Vincenzo; Toja Alessandro
EPI_ISL_613706, EPI_ISL_613710, EPI_ISL_613953, EPI_ISL_613955, EPI_ISL_614396, EPI_ISL_614397, EPI_ISL_614398, EPI_ISL_614889, EPI_ISL_637107, EPI_ISL_637108, EPI_ISL_637109, EPI_ISL_1180250, EPI_ISL_1180251, EPI_ISL_1180252, EPI_ISL_1180253, EPI_ISL_1191141, EPI_ISL_1191736, EPI_ISL_1191737, EPI_ISL_1191738, EPI_ISL_1191739, EPI_ISL_1229144, EPI_ISL_1229145, EPI_ISL_1229146, EPI_ISL_1229147, EPI_ISL_1229148, EPI_ISL_1229149, EPI_ISL_1311863, EPI_ISL_1380060, EPI_ISL_1380061, EPI_ISL_1380062, EPI_ISL_1380063, EPI_ISL_1380064, EPI_ISL_1380065, EPI_ISL_2098948, EPI_ISL_2240801, EPI_ISL_2258200			
see above	Laboratorio Biologia Molecolare Sars Cov2 - UOC Laboratorio Analisi - Servizio Medicina di Laboratorio, Ospedale "San Francesco" - ATS-ASSL Nuoro	Laboratorio specialistico UOC Ematologia - Ospedale "San Francesco" - ATS-ASSL Nuoro	; Angelo Domenico Palmas; Asproni Rosanna; Casu Gavino; Fancello Tatiana; Fiamma Maura; Floris Anna Rita; Garau Maria Cristina; Giovanna Piras; Giuseppe Mameli; Iana Lo Maglio; Lo Maglio Iana; Malune Paolo; Mameli Giuseppe; Mameli Giuseppe.; Maria Itria Monne; Maura Fiamma; Monne Maria Itria; Palmas Angelo Domenico; Paolo Malune; Piras Giovanna; Rosanna Asproni; Sanna Filomena; Sulis Vincenzo; Toja Alessandro
EPI_ISL_613560	Laboratorio Biologia Molecolare Sars Cov2 - UOC Laboratorio Analisi - Servizio Medicina di Laboratorio, Ospedale "San Francesco" - ATS-ASSL Nuoro Via Mannironi 1, 08100 Nuoro	Laboratorio specialistico UOC Ematologia - Ospedale "San Francesco" - ATS-ASSL Nuoro Nuoro	Asproni Rosanna; Casu Gavino; Fancello Tatiana; Fiamma Maura; Floris Anna Rita; Lo Maglio Iana; Mameli Giuseppe; Monne Maria Itria; Palmas Angelo Domenico; Piras Giovanna; Sanna Filomena; Sulis Vincenzo; Toja Alessandro
EPI_ISL_547965	Laboratorio Biologia Molecolare SarsCov2 UOC LAboratorio Analisi SErvizio Medicina di Laboratorio Ospedale San Francesco ATS-ASSL Nuoro	Laboratorio Specialistico UOC Ematologia Ospedale San Francesco - ATS ASSL NUORO	Asproni Rosanna; Casu Gavino; Fancello Tatiana; Fiamma Maura; Floris Anna Rita; Lo Maglio Iana; Mameli Giuseppe.; Monne Maria Itria; Palmas Angelo Domenico; Piras Giovanna; Sanna Filomena; Sulis Vincenzo; Toja Alessandro
EPI_ISL_2138923, EPI_ISL_2138924, EPI_ISL_2138925, EPI_ISL_2138926	Laboratorio HUB -Azienda Ospedaliero Universitaria - AOU – Cagliari	Laboratorio SPOKE Biologia Molecolare -Azienda Ospedaliero Universitaria - AOU – Cagliari	Alessandra Scano; Ferdinando Coghe; Germano Orrù; Miriam Loddo; Riccardo Cappai; Sara Fais; Valentina Medda
EPI_ISL_710503	Laboratorio specialistico UOC Ematologia - Ospedale "San Francesco" - ATS-ASSL Nuoro	Laboratorio specialistico UOC Ematologia - Ospedale "San Francesco" - ATS-ASSL Nuoro	Giovanna Piras
EPI_ISL_1789775	SC Microbiologia e Virologia AOUSS	AMES Centro Polidiagnostico Strumentale S.r.l.	Anna Puggioni; Bianca Paglietti; Caterina Serra; Claudia Piu; Elena Rimini; Erica Mura; Flavia Angioj; Gabriele Ibba; Giulia Rocca; Laura Firino; Rosalba Govoni; Salvatore Rubino; Sergio Uzzau.; Vincenzo Lai
