# Supplementary material for: Genomic Snapshot of SARS-CoV-2 in Migrants Entering Through Mediterranean Sea Routes
Source: Front Public Health. 2022 Mar 3;10:846115. doi: 10.3389/fpubh.2022.846115 (PMC8927662; doi:10.3389/fpubh.2022.846115)
Supplement: Supplementary file 3 [file Table_1.DOCX]

**Supplementary Table 1.** Nationality of the 133 migrants that were embarked at the port of Olbia in Eastern Sardinia (Italy) on September 25th.

| **Nationality** | **N of people** |
| --- | --- |
| Nigeria | 3 |
| Guinea | 34 |
| Ivory Coast | 9 |
| Mali | 28 |
| Sierra Leone | 4 |
| Burkina Faso | 2 |
| Cameroon | 2 |
| Niger | 1 |
| Senegal | 3 |
| Togo | 1 |
| Gambia | 1 |
| Bangladesh | 2 |
| Egypt | 16 |
| Yemen | 1 |
| Sudan | 4 |
| Morocco | 4 |
| Libya | 18 |
