# Supplementary material for: Genomic Snapshot of SARS-CoV-2 in Migrants Entering Through Mediterranean Sea Routes
Source: Front Public Health. 2022 Mar 3;10:846115. doi: 10.3389/fpubh.2022.846115 (PMC8927662; doi:10.3389/fpubh.2022.846115)
Supplement: Supplementary file 4 [file Table_2.docx]

**Supplementary Table 2.** Information about the 10 migrants that tested positive for SARS-CoV-2 during the initial screening.

| Case n° | Nationality | Age | Sex | Mean CT value | Comorbidities | Sequence  (Accession number) |
| --- | --- | --- | --- | --- | --- | --- |
| 1 | Guinea | 17 | M | 37 | No comorbidities | - |
| 2 | Guinea | 18 | M | 26 | No comorbidities. Seasickness. Pain in the right shoulder, no injuries | 310 ([NC_OL944327](https://www.ncbi.nlm.nih.gov/nuccore/OL944327.1/)) |
| 3 | Sudan | 26 | M | 37 | No comorbidities | - |
| 4 | Sudan | 38 | M | 32 | No comorbidities | - |
| 5 | Sudan | 23 | M | 36 | No comorbidities | - |
| 6 | Guinea | 16 | M | 37 | No comorbidities | - |
| 7 | Guinea | 17 | M | 37 | No comorbidities. Old scar on the back due to an accident | - |
| 8 | Togo | 17 | M | 36 | No comorbidities. Headache. Painful urination from 5 days. Confirmed Malaria from 8 days. No medication. | - |
| 9 | Gambia | 17 | M | 39 | No comorbidities | - |
| 10 | Burkina Faso | 22 | F | 35 | Diabetes, wound on left palmar (5 mm) from entering the rubber boat 18^th^ September 4 pm. | - |
